# Safety, pharmacokinetics and exploratory pro-cognitive effects of HTL0018318, a selective M_1_ receptor agonist, in healthy younger adult and elderly subjects: a multiple ascending dose study

**DOI:** 10.1186/s13195-021-00816-5

**Published:** 2021-04-21

**Authors:** Charlotte Bakker, Tim Tasker, Jan Liptrot, Ellen P. Hart, Erica S. Klaassen, Robert Jan Doll, Giles A. Brown, Alastair Brown, Miles Congreve, Malcolm Weir, Fiona H. Marshall, David M. Cross, Geert Jan Groeneveld, Pradeep J. Nathan

**Affiliations:** 1 Centre for Human Drug Research (CDHR), Zernikedreef 8, 2333 CL Leiden, The Netherlands; 2 Sosei Heptares, Steinmetz Building, Granta Park, Great Abington, Cambridge, CB21 6DG UK; 3 OMass Therapeutics Ltd, Oxford, UK; 4 Merck Sharp & Dohme Limited, London, UK; 5 Cross Pharma Consulting Limited, Cambridge, UK; 6 Leiden University Medical Centre, Leiden, The Netherlands; 7 Department of Psychiatry, University of Cambridge, Cambridge, UK; 8 School of Psychological Sciences, Monash University, Melbourne, Australia

**Keywords:** Muscarinic M_1_, M_1_ receptor, Cholinergic, Dementia, Safety, Pharmacokinetics, Cognition, Memory, Healthy subjects

## Abstract

**Background:**

The cholinergic system and M_1_ receptor remain an important target for symptomatic treatment of cognitive dysfunction. The selective M_1_ receptor partial agonist HTL0018318 is under development for the symptomatic treatment of Dementia’s including Alzheimer’s disease (AD) and dementia with Lewy bodies (DLB). We investigated the safety, tolerability, pharmacokinetics and exploratory pharmacodynamics of multiple doses of HTL0018318 in healthy younger adults and elderly subjects.

**Methods:**

This randomised, double blind, placebo-controlled study was performed, investigating oral doses of 15–35 mg/day HTL0018318 or placebo in 7 cohorts of healthy younger adult (*n* = 36; 3 cohorts) and elderly (*n* = 50; 4 cohorts) subjects. Safety, tolerability and pharmacokinetic measurements were performed. Pharmacodynamics were assessed using a battery of neurocognitive tasks and electrophysiological biomarkers of synaptic and cognitive functions.

**Results:**

HTL0018318 was generally well-tolerated in multiple doses up to 35 mg/day and were associated with mild or moderate cholinergic adverse events. There were modest increases in blood pressure and pulse rate when compared to placebo-treated subjects, with tendency for the blood pressure increase to attenuate with repeated dosing. There were no clinically significant observations or changes in blood and urine laboratory measures of safety or abnormalities in the ECGs and 24-h Holter assessments. HTL0018318 plasma exposure was dose-proportional over the range 15–35 mg. Maximum plasma concentrations were achieved after 1–2 h. The apparent terminal half-life of HTL0018318 was 16.1 h (± 4.61) in younger adult subjects and 14.3 h (± 2.78) in elderly subjects at steady state. HTL0018318 over the 10 days of treatment had significant effects on tests of short-term (working) memory (*n*-back) and learning (Milner maze) with moderate to large effect sizes.

**Conclusion:**

Multiple doses of HTL0018138 showed well-characterised pharmacokinetics and were safe and generally well-tolerated in the dose range studied. Pro-cognitive effects on short-term memory and learning were demonstrated across the dose range. These data provide encouraging data in support of the development of HTL0018138 for cognitive dysfunction in AD and DLB.

**Trial registration:**

Netherlands Trial Register identifier NTR5781. Registered on 22 March 2016.

**Supplementary Information:**

The online version contains supplementary material available at 10.1186/s13195-021-00816-5.

## Background

Alzheimer’s disease (AD) and dementia with Lewy bodies (DLB) are progressive neurodegenerative disorders caused by complex pathophysiological processes [1], leading to degeneration of the cholinergic neurons in the basal forebrain and their projections to the cortex and the hippocampus [2]. These cholinergic deficits play a key role in the underlying cognitive impairments as well as some of the behavioural and psychiatric symptoms including visual hallucinations [3–7].

The complex pathology of AD and DLB has hampered progress toward a curative treatment. Currently, the only available treatment is symptomatic and consists mostly of acetylcholinesterase inhibitors. Acetylcholinesterase inhibitors (ChEIs) inhibit the breakdown of the neurotransmitter acetylcholine (ACh) and subsequently prolong the availability of ACh at the synapse. This leads to activation of cholinergic muscarinic and nicotinic receptors in the neocortex and hippocampus, which are involved in cognitive function. Despite their ability to improve cognition, ChEIs demonstrate only modest clinical efficacy, likely due to ongoing neurodegeneration of cholinergic neurons in dementia including AD and associated decrease of ACh synthesis, and by a limited dosing range of ChEIs because of side effects including gastrointestinal side effects linked to indirect stimulation of peripheral muscarinic M_2_ and M_3_ receptors [8–11]. Therefore, despite efforts to develop disease-modifying treatments for AD, there is a need for improved symptomatic treatments for AD and other dementia’s targeting not only cognitive symptoms but behavioural and psychiatric symptoms. Optimization of the current treatment options can be achieved by targeting post synaptic muscarinic receptors, in particular M_1_ receptors involved in cognitive function relative to other muscarinic receptors (i.e. M_2_ and M_3_ receptors) associated with peripheral side effects. Such selectivity might allow for higher dosing, which could contribute to improved efficacy for certain cognitive and/or behavioural symptoms. Hence selective M_1_ receptor agonists may be promising drugs for the treatment of cognitive, behavioural and psychological symptoms in psychiatric and neurological disorders (for a review, see Erskine et al. [12]).

The M_1_ muscarinic acetylcholine receptor (mAChR) is the predominant mAChR in the central nervous system and is highly expressed in the neocortex and hippocampus [13, 14]. Pre-clinical studies suggest M_1_ agonists can improve cognitive function including learning and memory [15–19]. Consistent with this evidence, muscarinic receptor agonists including the M_1_/M_4_ agonist Xanomeline and the M_1_ agonist GSK1034702 have shown promising early clinical effects [16, 20]. Xanomeline showed improvement in global cognitive function (i.e. ADAS-Cog), general clinical status (i.e. CIBIC+), and behavioural symptoms such as delusions, hallucinations, and agitation [20]. Similarly, the M_1_ agonist GSK1034702 was shown to improve episodic memory (international shopping list task of the Cogstate battery) in a nicotine abstinence model of cognitive dysfunction [16].

HTL0018318 (ethyl (3-endo)-3-(3-oxo-2,8-diazaspiro [4.5] dec-8-yl)-8-azabicyclo [3.2.1] octane-8-carboxylate hydrochloride), is a partial M_1_ mAChR agonist that has been developed for the symptomatic treatment of cognitive, behavioural and psychiatric symptoms in dementias including AD and DLB. In pre-clinical studies, HTL0018318 was found to be highly selective for the M_1_ receptor with an EC_50_ of approximately 100 nM and with twofold selectivity for the M_1_ over M_4_ receptors with no detectable functional agonist activity at human M_2_ and M_3_ receptors. Pre-clinical studies have shown HTL0018318 to reverse scopolamine-induced deficits in passive avoidance learning in rats consistent with pro-cognitive effects reported with other M_1_ agonists on tests of learning and memory. The single ascending dose (SAD) study with HTL0018318 has shown that single doses of HTL0018318 up to 35 mg were relatively well-tolerated in healthy younger adult and elderly subjects [21]. HTL0018318 was absorbed rapidly, peak plasma concentration was typically reached 1–2 h post-dose and the average elimination half-life was 12–16 h. Approximately 30% of the plasma unbound concentration entered the cerebral spinal fluid. No consistent significant effects on exploratory pharmacodynamic (PD) tests were observed. The SAD study was followed by the current multiple-dose escalation study, in which we aimed to investigate the safety, tolerability, pharmacokinetics (PK) and exploratory PD of HTL0018318 in healthy subjects.

## Methods

### Design

**Fig. 1:**
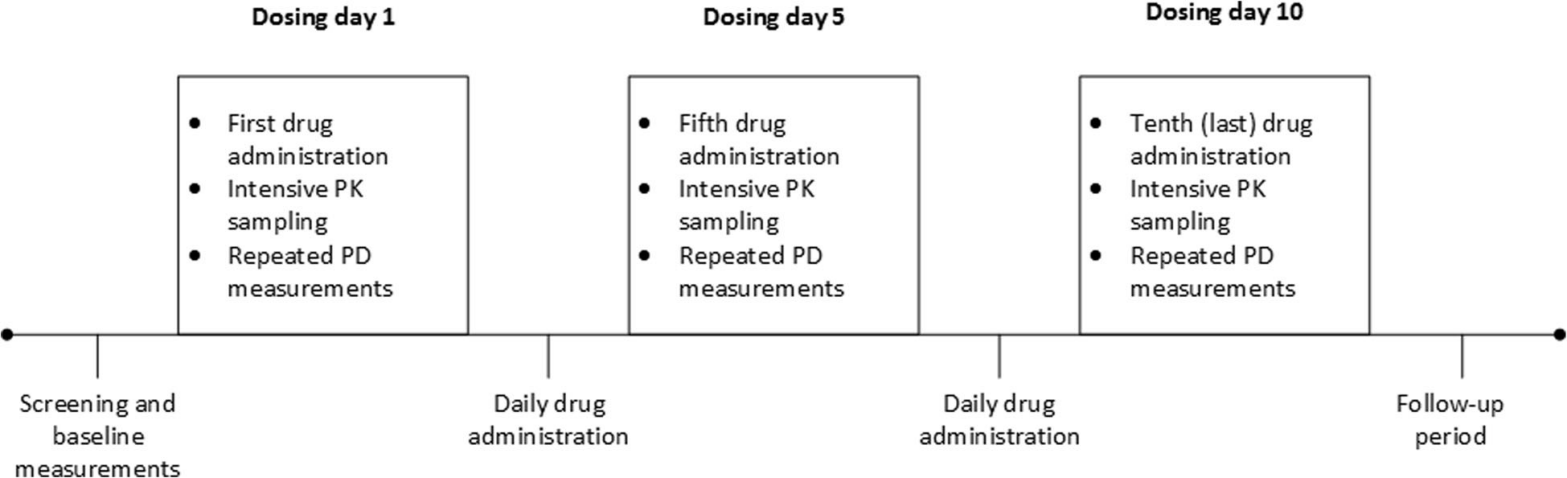
Study design

This was a double-blind, randomised, placebo-controlled, parallel group study that consisted of 7 cohorts in total: 3 cohorts of 12 younger adult healthy subjects and 4 cohorts of 12 healthy elderly subjects. The study design is shown in Fig. 1. The study was approved by the medical ethics review board of the foundation Beoordeling Ethiek Biomedisch Onderzoek (BEBO, Assen, The Netherlands) and conducted according to the principles of the Declaration of Helsinki and the ICH GCP guidelines [22].

### Participants

Healthy younger adult subjects aged 18–55 years and healthy elderly subjects aged 65 years and over, both male and female, were enrolled to participate in the study. Subjects were eligible if they were non-smokers, in good health, with a resting systolic blood pressure between 90 and 140 mmHg, diastolic blood pressure between 50 and 90 mmHg and a heart rate between 45 and 100 bpm at screening. Main exclusion criteria were current or past history of any physical, neurological or psychiatric illness and currently on any medication including antihypertensive drugs.

### Investigational product

HTL0018318, in this study administered as the HCl salt, was administered as a 100 ml oral solution. Water was used as placebo. To mask the difference in taste between HTL0018318 and placebo, a peppermint strip (Listerine) was administered 1 min before and after the administration of HTL0018318. Subjects were not asked if they could guess whether they received HTL0018318 or placebo as the study was a parallel group design with taste related unmasking having minimal impact on unblinding.

Dose levels of 15 mg, 20 mg, 25 mg or placebo were administered in a ratio 9:3 (active:placebo) once a day for 10 consecutive days in both younger adult and elderly subjects. The dose level of 35 mg was only studied in a cohort with elderly subjects (8 subjects on HTL0018318 and 4 subjects on placebo) and administered to 3 subjects (including one replacement); however, lack of tolerability led to a titration regimen of 20 mg HTL0018318 once a day for 5 consecutive days followed by 35 mg HTL0018318 once a day for 10 consecutive days (7 subjects on HTL0018318 and 3 subjects on placebo). Before implementing this new dosing regimen, the protocol was amended and approved.

HTL0018318 dosages were based on the tolerated dose range of 1–35 mg in the SAD study.

### Safety and tolerability assessments

The investigated safety end points were adverse events (AEs) collected and recorded on the first dosing day, continuing until the follow-up visit, safety laboratory sampled at regular intervals, vital signs and electrocardiogram (ECG) conducted daily pre-dose and 1 h post-dose, and with a higher frequency on the 1st, 5th and 10th dosing day, 24-h Holter registration performed at the 1st and 10th dosing day and pulmonary function tests (PFT). Cholinergically mediated AEs (i.e. AEs with a (possible) relationship to increased cholinergic stimulation) were identified. AEs in this category included; hyperhidrosis, salivary hypersecretion, hypertension, nausea, diarrhoea, vomiting, constipation, insomnia, somnolence, dizziness, muscle spasms, hot flush, cold sweat and piloerection.

### Pharmacokinetics assessments

Venous blood samples for PK analysis were obtained pre-dose, 15 min, 30 min, 1 h, 1.5 h, 2 h, 3 h, 4 h, 6 h, 8 h, 9 h and 12 h post-dose on dosing days 1 and 10. On dosing day 5, blood PK samples were obtained pre-dose and at 1 h, 3 h, 6 h and 9 h post-dose. On the remaining days, only trough samples were taken. Urine for PK analyses was collected up to 24 h after the first drug administration and up to 72 h after the last drug administration. In subjects who were administered 35 mg HTL0018318 according to the up-titration schedule, extra PK samples were collected pre-dose on each day and 1, 3, 6 and 9 h after the 5th administration of 20 mg. Plasma and urine concentrations of HTL0018318 were determined using validated bioanalytical methods involving protein precipitation and liquid chromatography coupled with tandem mass spectrometry. The analytical range of the assay was 0.5–1000 ng/ml.

PK parameters included in the analysis were the maximum observed plasma concentration (*C*_max_); time to *C*_max_ (*t*_max_); area under the plasma-concentration-time curve (AUC) zero to the last measurement (AUC_last_), from zero to the end of the dose interval (AUC_0-tau_) and from zero to infinity (AUC_0-inf_); time of the minimum concentration (*t*_last_); the minimum concentration within the dosing interval (*C*_min_); apparent elimination half-life (*t*_1/2_); apparent oral clearance (CL/F); apparent volume of distribution (Vz/F); renal clearance (CLr); percentage of dose excreted renally as unchanged drug (Ae%); and coefficient of variation (%CV). Non-compartmental analyses were performed on the PK data using Phoenix 64 build 6.4.0.768 using WinNonlin 6.4. Statistical analysis was performed in R version 3.3.1 (2016-06-21).

### Exploratory pharmacodynamic assessments

To assess the acute effects of HTL0018318 on central nervous system (CNS) functioning, exploratory PD tests were performed with use of the NeuroCart, a test battery assessing a wide range of CNS domains, developed to examine the acute PD effects of CNS-active drugs and previously shown to be sensitive to cholinergic modulation [23–27]. A customised set of tasks to detect PD effects to be expected from a cholinergic drug was performed pre-dose, 1 h, 3 h, 5 h and 9 h post-dose on dosing days 1, 5 and 10. On dosing day 1, the pre-dose measurements were performed twice.

The following NeuroCart tests were performed: the adaptive tracking test measured attention and visuomotor coordination; the Milner maze test (MMT) was used to evaluate spatial working memory, learning and executive function; the *n*-back task was used to assess (short-term) working memory; pupil size was measured to monitor any drug effects on the sympathetic nervous system; synaptic/network activity was assessed using electrophysiology and included resting electroencephalogram (EEG) (power in delta, theta, alpha, beta and gamma bands) and event-related potentials (ERP) (P300 and Mismatch negativity (MMN)); a visual analogue scale (VAS) according to Bond and Lader was used to subjectively assess alertness, mood and calmness; and a VAS nausea was used to evaluate subjective nausea. The Leeds Sleep Evaluation Questionnaire (LSEQ) was used to assess changes in sleep quality. Detailed task descriptions are provided in the supplement methods section.

Saliva production was assessed by measuring the change in weight of three Salivette® dental rolls put into the oral cavity for 3 min. Pulmonary function was measured using the Spirostik (distributed by Accuramed), a PC-based open spirometry system. Vital signs were also analysed.

### Statistics

No formal hypothesis testing was conducted. The sample size was chosen based on a compromise between minimising the exposure of human subjects to a new chemical entity and the need to provide sufficient data. Hence, the study was not powered to detect any significant treatment effects of small to moderate effect sizes.

The repeatedly measured PD endpoints on dosing days 1, 5 and 10 were analysed with a mixed model analysis of covariance (ANCOVA) with treatment, time, treatment by time, group, treatment by group, group by time and treatment by group by time as fixed factors and subject as random factor and the average baseline measurement as covariate. Least-square means (LSMs) and 95% confidence intervals (CIs) were estimated from the ANCOVA models. The repeatedly measured PD parameters of the subjects dosed 35 mg according to an up-titration schedule were analysed together with the placebo subjects of the other elderly cohorts to increase the power. The data after the up-titration was analysed with a mixed model ANCOVA with treatment, time and treatment by time as fixed factors, subject as random factor and the average baseline measurement before the up-titration as covariate. All subjects who received at least one dose of study treatment were included in the safety and PD analysis set. PD data of the two subjects who were administered 35 mg HTL0018318 not according to an up-titration schedule were not analysed. The following contrasts were calculated for dosing days 1, 5 and 10 separately for every dose level: HTL0018318 (younger adults + elderly subjects) vs placebo (all placebo subjects pooled together); HTL0018318 (younger adult subjects) vs placebo (younger adult placebo subjects pooled together); and HTL0018318 (elderly subjects) vs placebo (elderly placebo subjects pooled together).

For all outcome parameters, the mean, standard deviation, 95% CI and effect sizes were calculated. All calculations were performed using SAS for windows V9.4 (SAS Institute, Inc., Cary, NC, USA). MMN data were excluded from statistical analysis due to limited data quality and technical issues with stimuli timing and recording. Hence, only resting state EEG power data and P300 data were reported here.

## Results

### Subjects

**Fig. 2:**
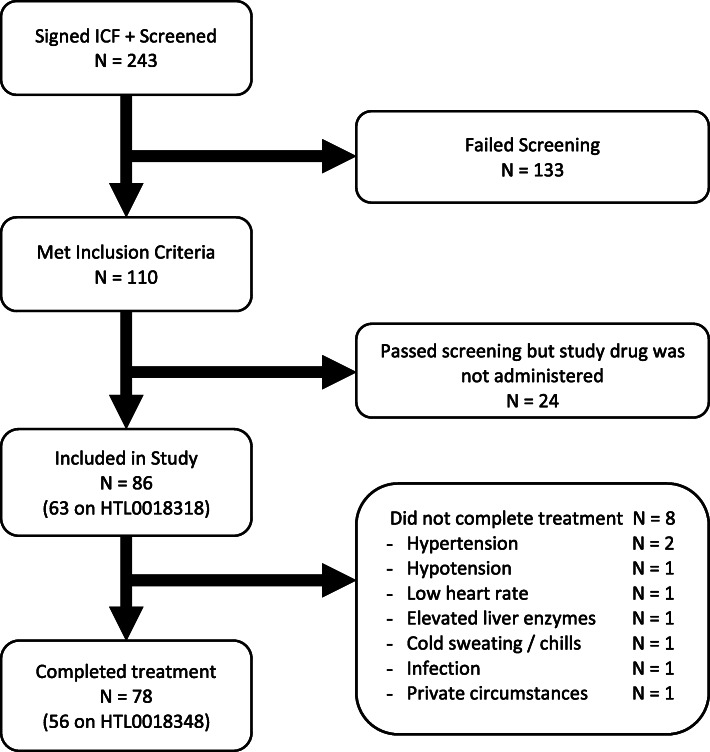
Subject disposition flow chart

A total of 36 healthy younger adult subjects with a mean age of 30 years (range 18–53) and with a mean body mass index (BMI) of 23.7 kg/m^2^ (range 18–31) were enrolled. A total of 50 healthy elderly subjects, with a mean age of 69.9 years (range 65–83) and with a mean BMI of 25.9 kg/m^2^ (range 20.5–32.5) were included. See Fig. 2 for subject disposition flow chart. The AEs leading to withdrawal of six subjects are described below. In one elderly subject the third dose of 25 mg HTL0018318 was not administered due to infection-like symptoms and elevated c-reactive protein. The subject subsequently recovered spontaneously and dosing was resumed.

### Safety and tolerability

Multiple doses of 15, 20 and 25 mg HTL0018318 were generally well-tolerated by healthy younger adult and elderly subjects. The dose level of 35 mg without up-titration period was not tolerated by elderly subjects. The 2 subjects dosed with 35 mg without up-titration period were withdrawn from the study due to AEs (hypertension and cold sweat) after the 1st or 2nd administration of HTL0018318. Consequently, it was decided to stop dosing 35 mg without an up-titration period in the remaining subjects of this cohort and to add an up-titration period preceding the 35 mg doses. This was relatively well-tolerated with only mild AEs and no withdrawn subjects. Overall, more subjects dosed with HTL0018318 reported AEs compared to subjects dosed with placebo (Tables 1 and 2). In younger adults, the number of subjects reporting AEs in general and the number of cholinergically mediated AEs appeared to be treatment-related. However, no clear dose-response relationship was observed. In elderly, the number of subjects reporting AEs in general and the number of cholinergically mediated AEs appeared to be treatment- and dose-related.

**Table 1:** Overview of (potentially cholinergically mediated) AEs reported by younger adult subjects

MedDRA-preferred term	Placebo *n*=9	15 mg *n*=9	20 mg *n*=9	25 mg *n*=9	All HTL0018318 *n*=27
All AEs	5 (55.6)	8 (88.9)	6 (66.7)	6 (66.7)	20 (74.1)
All cholinergic AEs	1 (11.1)	5 (55.6)	5 (55.6)	6 (66.7)	16 (59.3)
Hypertension	0	0	0	0	0
Nausea	0	3 (33.3)	2 (22.2)	4 (44.4)	9 (33.3)
Diarrhoea	0	1 (11.1)	1 (11.1)	0	2 (7.4)
Vomiting	0	0	0	1 (11.1)	1 (3.7)
Hypersalivation	0	0	0	1 (11.1)	1 (3.7)
Hyperhidrosis	0	1 (11.1)	4 (44.4)	3 (33.3)	8 (29.6)
Constipation	0	0	0	0	0
Chills	0	1 (11.1)	0	0	1 (3.7)
Cold sweat	0	0	0	0	0
Feeling cold	0	0	1 (11.1)	0	1 (3.7)
Feeling hot	0	0	1 (11.1)	0	1 (3.7)
Feeling of body temperature change	0	0	0	0	0
Hot flush	0	0	1 (11.1)	2 (22.2)	3 (11.1)
Piloerection	0	0	2 (22.2)	0	2 (7.4)
Peripheral coldness	0	0	0	0	0
Insomnia	1 (11.1)	0	0	0	0
Dizziness	0	0	1 (11.1)	1 (11.1)	2 (7.4)
Muscle spasm	0	1 (11.1)	0	0	1 (3.7)

Data are shown as number (percentage) of subjects reporting AEs

**Table 2:** Overview of (potentially cholinergically mediated) AEs reported by elderly subjects

MedDRA-preferred term	Placebo *n*=14	15 mg *n*=9	20 mg *n*=9	25 mg *n*=9	35 mg *n*=2	35 mg + up-titration *n*=7	All HTL0018318 *n*=36
All AEs	8 (57.1)	7 (77.8)	7 (77.8)	9 (100.0)	2 (100.0)	7 (100.0)	32 (88.9)
All cholinergic AEs	2 (14.3)	1 (11.1)	5 (55.6)	7 (77.8)	2 (100)	7 (100)	22 (61.1)
Hypertension	0	0	0	1	1	1	3 (8.3)
Nausea	0	1 (11.1)	1 (11.1)	2 (22.2)	0	0	4 (11.1)
Diarrhoea	0	0	0	1 (11.1)	0	0	1 (2.8)
Vomiting	0	0	0	0	0	0	0
Constipation	1 (7.1)	0	0	0	0	0	0
Hypersalivation	0	0	0	0	0	0	0
Hyperhidrosis	0	0	3 (33.3)	5 (55.6)	0	2 (28.6)	10 (27.8)
Chills	0	0	0	1 (11.1)	0	4 (57.1)	5 (13.9)
Cold sweat	0	0	0	0	1 (50.0)	2 (28.6)	3 (8.3)
Feeling cold	1 (7.1)	0	1 (11.1)	2 (22.2)	1 (50.0)	2 (28.6)	6 (16.7)
Feeling hot	0	0	0	2 (22.2)	0	0	2 (5.6)
Feeling of body temperature change	0	0	0	1 (11.1)	0	0	1 (2.8)
Hot flush	0	0	2 (22.2)	1 (11.1)	0	2 (28.6)	5 (13.9)
Peripheral coldness	0	0	0	0	1 (50.0)	0	1 (2.8)
Piloerection	0	0	0	0	0	0	0
Insomnia	0	0	1	0	0	0	1 (2.8)
Dizziness	1	0	0	3	0	0	3 (8.3)
Muscle spasm	0	0	1	0	0	0	1 (2.8)

Data are shown as number of subjects reporting adverse events (percentage of subjects)

The most frequently occurring cholinergically mediated AEs were nausea, hyperhidrosis, chills, cold sweat, somnolence and feeling cold (see Tables 1 and 2).

In total, 6 subjects were withdrawn from the study because of AEs. One elderly subject experienced severe cold sweats and chills after 1 administration of 35 mg HTL0018318 without up-titration period. One elderly subject was withdrawn from the study after 2 administrations of 35 mg HTL0018318 without up-titration period due to hypertension (supine systolic blood pressure of 168/84 mmHg, increase of > 40% from baseline). One elderly subject was withdrawn after 1 administration of 20 mg during the period preceding the 35 mg dose due to a 40% increase of supine blood pressure to 196/99 mg Hg compared to baseline (140/62 mmHg). One subject was withdrawn from further participation after 6 administrations of 25 mg HTL0018318 due to elevated liver enzymes AST (76 U/L) and ALT (127 U/L). The AEs of these 4 subjects were considered to be related to the study drug. One younger adult subject was withdrawn after 4 administrations of 25 mg HTL0018318 because of episodes of bradycardia down to 38 bpm in combination with nausea and fatigue. These episodes of bradycardia were also observed on the 24-h Holter monitoring which was part of the screening and were therefore not considered to be drug-related. One elderly subject was withdrawn because of orthostatic hypotension 24 h after the first 25 mg administration. The supine blood pressure decreased from 106/48 mmHg to 66/37 mmHg. As there was no clear relation to peak plasma HTL0018318 concentration, this AE was not considered to be related to the study drug.

No consistent clinically relevant abnormalities in haematology blood results, urinalysis, ECGs and 24-h Holter monitoring were observed in both younger adult and elderly subjects. No serious AEs or deaths occurred. There were no chemistry blood results that showed an apparent trend toward increased incidence with ascending dose levels of HTL0018318 during the study.

### Pharmacokinetic parameters

**Fig. 3:**
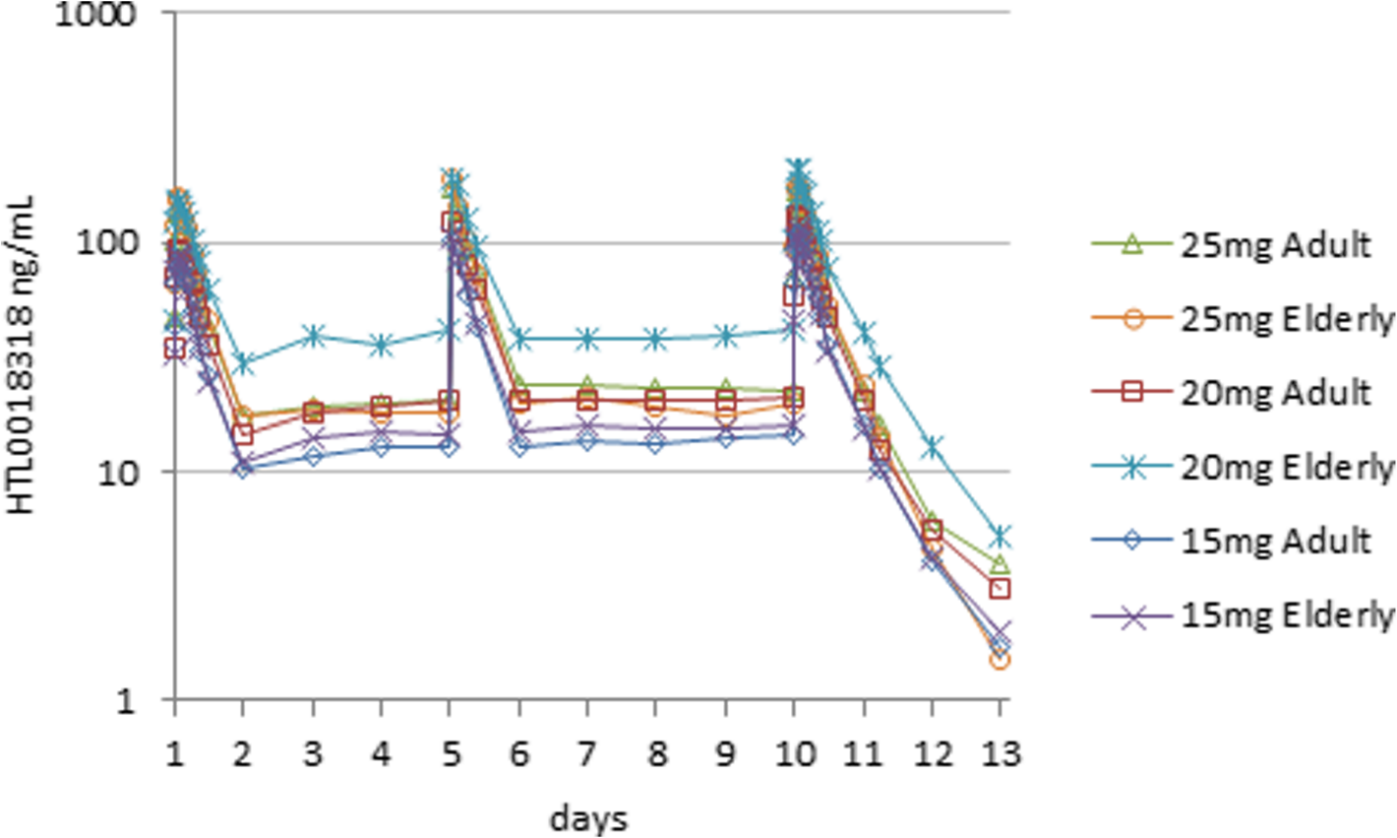
HTL0018318 mean plasma concentration after 15, 20 or 25 mg for younger adult subjects

**Fig. 4:**
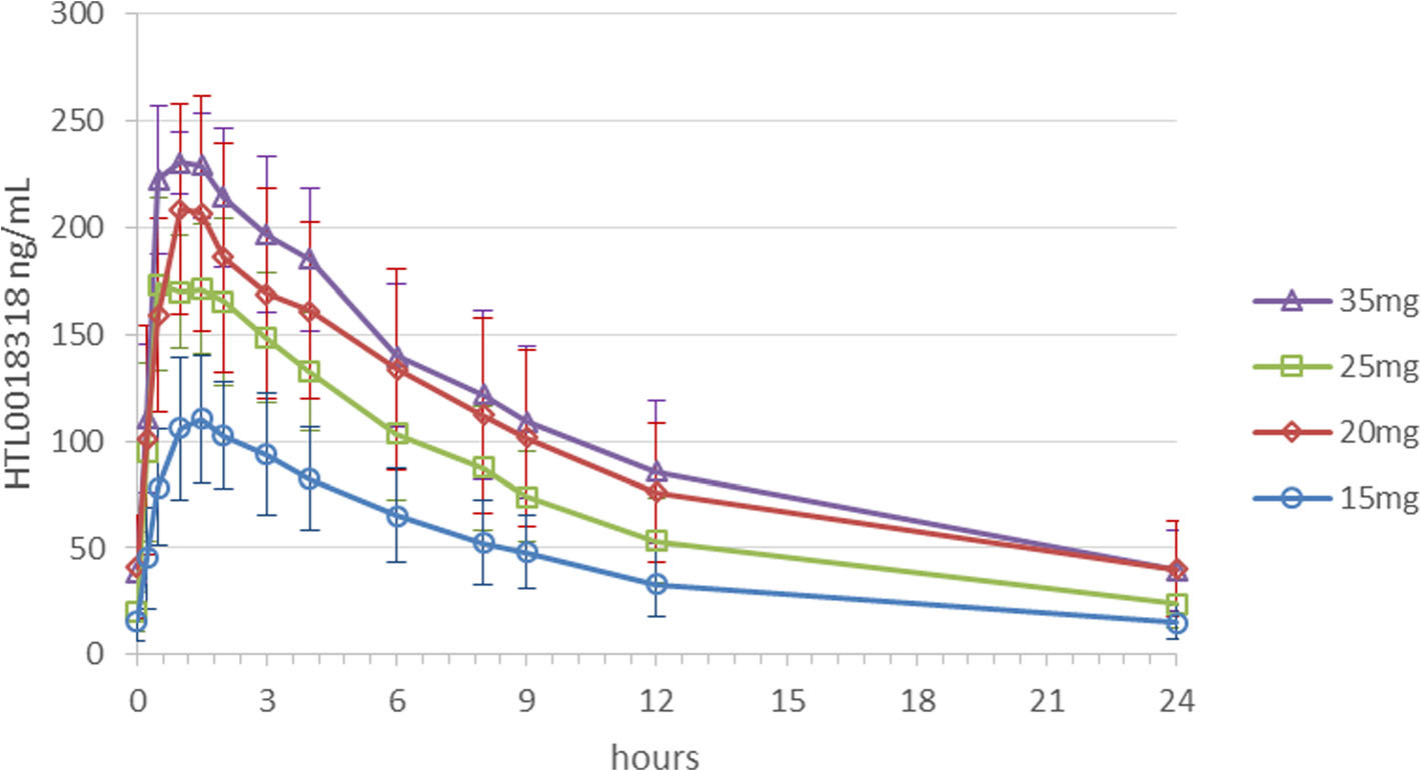
HTL0018318 mean plasma concentration after 15, 20, 25 or 35 mg for elderly subjects

The PK parameters and mean concentration-time profiles of HTL0018318 are shown in Figs. 3 and 4. These figures show HTL0018318 arithmetic mean plasma concentration against time of 10 daily oral doses of 15, 20 or 25 mg for younger adult subjects (Fig. 3) and additionally mean (± SD) plasma concentrations of 35 mg in elderly subjects (Fig. 4). The absorption was rapid with a median *T*_max_ of 1 h post-dose (range 0.5 to 4 h). The oral PK profile was biphasic after *C*_max_. The mean (SD) apparent terminal half-life in healthy subjects was 16.1 h (± 4.61) in younger adult subjects and 14.3 h (± 2.78 h) in elderly subjects determined up to 72 h post-dose. A mean accumulation index of 1.32 was seen in younger adult and 1.29 in elderly subjects following the 10th dose, in terms of AUC_0–24_. Steady state was reached in approximately two or three doses. The ratio between the mean multiple dose AUC_0-tau_ to the AUC_0-inf_ after the 1st dose was 1.06 following the 5th and 1.14 following the 10th dose in younger adults and 1.03 following the 5th and 1.07 following the 10th dose in elderly subjects. On average, elderly subjects appeared to have lower oral clearance than the younger adults (mean (SD) CLss/F 18.3 (± 6.68) L/h in younger adult and 16.0 (± 6.79) L/h in elderly subjects). The inter-individual variability in *C*_max_, AUC and *t*_½_ was moderate, with a %CV typically < 30 and not larger than 66 for any variable.

Exposure to HTL0018318 was dose-proportional over the range 15 to 35 mg. Exposure in elderly subjects given 20 mg was higher than expected; however, dose-exposure proportionality did not deviate from linearity assessed using the power model [28]. The reason for higher exposure at 20 mg in elderly subjects could not be determined.

Renal elimination was a major route of clearance. The mean percentage recovery of HTL0018318 over the 24-h dose interval following the 10th dose was 58.1% in younger adults (range 22.3 to 95.3) and 50.4% in elderly subjects (range 25.8 to 95.7%), which represents the minimum absolute oral bioavailability. The mean renal clearance was 8.62–8.84 L/h in younger adults and 6.03–6.23 L/h in elderly subjects.

#### Central pharmacodynamic biomarkers

HTL0018318 daily dosing for 10 days showed no consistent effects on EEG/ERP, saliva production, LSEQ, pupil size or VAS scores compared to placebo (table in result supplement). Although the study was not powered to detect small to moderate pro-cognitive effects of HTL0018318, some significant differences compared to placebo were observed on a number of cognitive tests including adaptive tracking (a measure of psychomotor function and attention), the *n*-back test (a measure of working memory) and the Milner Maze Test (a measure of learning and memory).

#### Adaptive tracking test

**Fig. 5:**
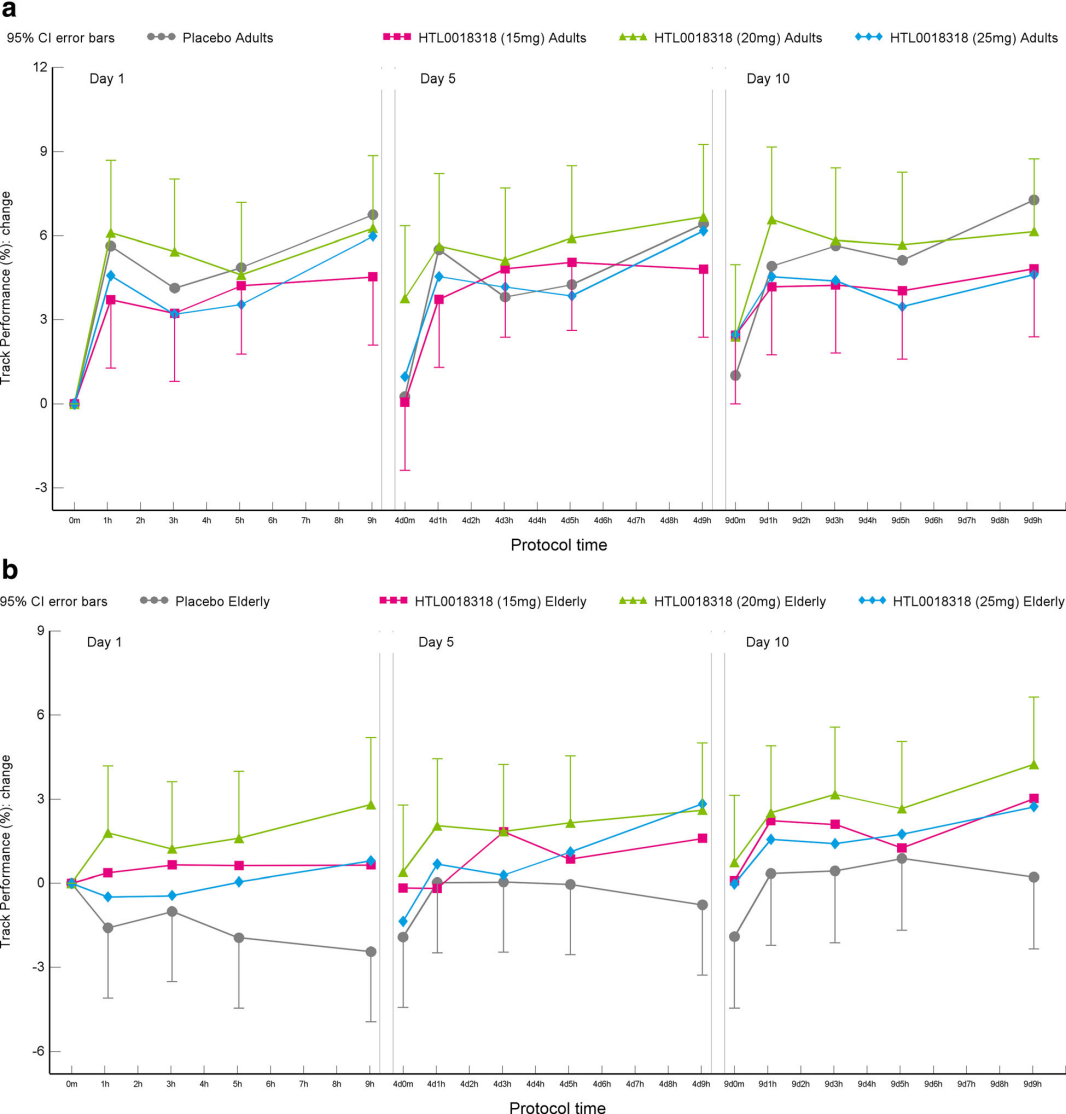
Effects on adaptive tracking (% correctly tracked) in younger adults (**a**) and elderly subjects (**b**)

Overall, HTL0018318 had no significant effects on the adaptive tracking test in young and elderly subjects across doses and testing days. However, after administration of 20 mg HTL0018318, the time correctly tracked was improved by 3.605%-point (95% CI [0.672–6.539], *p* = 0.0167) compared with placebo, on dosing day 1 in the elderly subjects (see Fig. 5, data shown as estimate of the change from baseline performance.).

#### *N*-back test

Overall, an improvement in performance in both younger adult and elderly subjects on the *n*-back test (0-, 1- and 2-back conditions) was observed following administration of all dose levels of HTL0018318 compared with placebo. The effect on the performance on the most relevant 2-back working memory “accuracy” measure is reported here. In all observations, a higher (number correct-number incorrect)/total was observed indicating better performance. See supplement results section for data on the 0-back and 1-back (accuracy and reaction time) and 2-back (reaction time) conditions.

**Fig. 6:**
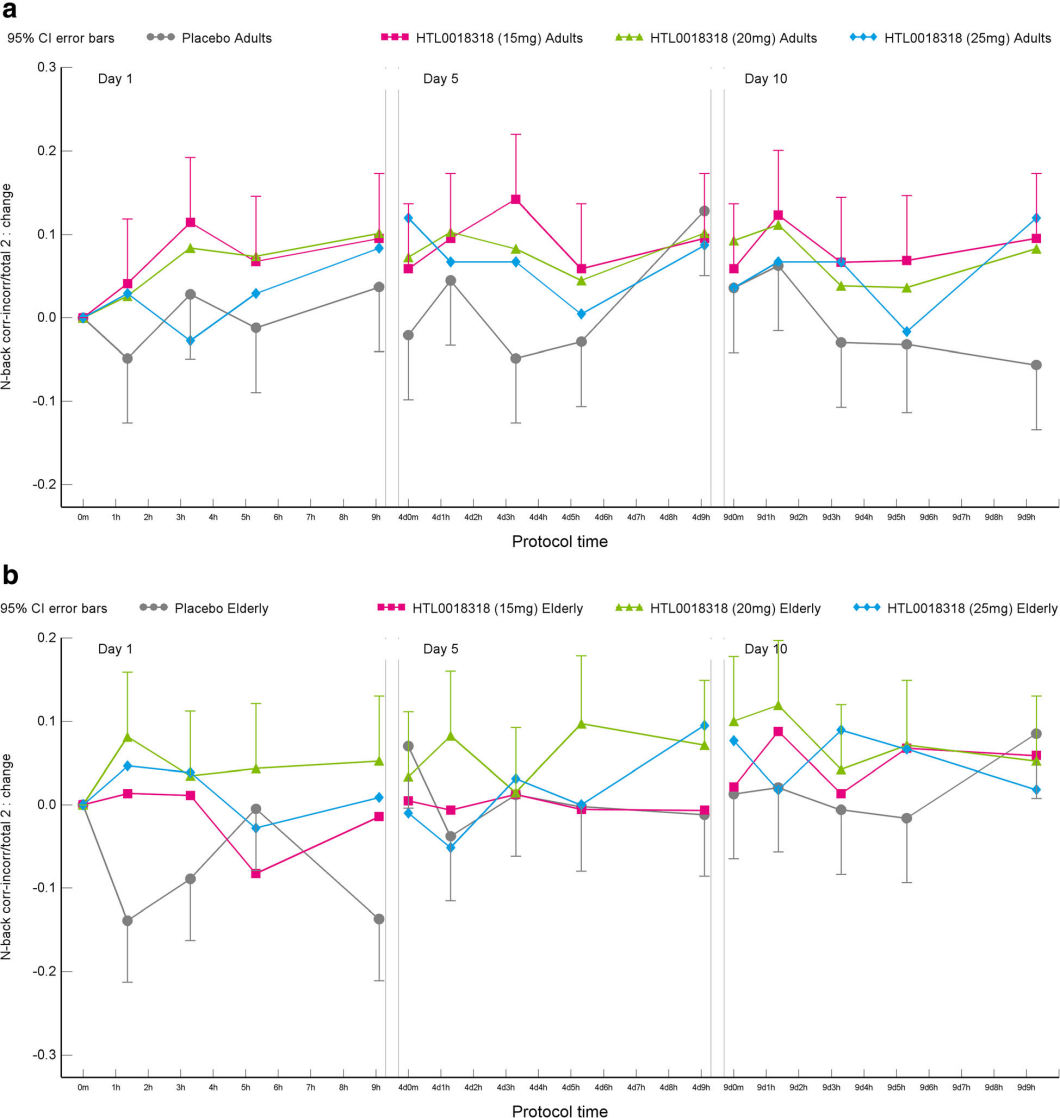
Effects on the 2-back accuracy in younger adults (**a**) and elderly subjects (**b**)

After administration of 15 mg HTL0018318, the 2-back accuracy score was 0.076 higher on dosing day 1 (95% CI [0.028–0.125], *p* = 0.0022) and 0.069 higher on dosing day 10 (95% CI [0.020–0.118], *p* = 0.0058) compared with placebo. Following administration of 20 mg HTL0018318, the 2-back accuracy score was 0.108 higher on dosing day 1 (95% CI [0.059–0.157], *p* = <.0001), 0.068 higher on dosing day 5 (95% CI [0.019–0.117], *p* = 0.0073) and 0.066 higher on dosing day 10 (95% CI [0.016–0.115], *p* = 0.0095), compared with placebo. After administration of 25 mg HTL0018318, the 2-back accuracy score was 0.068 higher on dosing day 1 (95% CI [0.020–0.117], *p* = 0.0063) compared with placebo. These data were analysed separately for younger adult and elderly subjects. This is presented in Table 3 and Fig. 6.

**Table 3:** Effects on the accuracy of the 2-back performance compared with placebo

	Younger adults			Elderly		
Parameter	Day 1	Day 5	Day 10	Day 1	Day 5	Day 10
**15 mg HTL0018318**						
*N*-back corr-incorr/total 2	0.079 (0.009, 0.148) ***p*** **= 0.0265** ES = 1.06	0.074 (0.005, 0.143) ***p*** **= 0.0361** ES = 1.00	0.102 (0.033, 0.172) ***p*** **= 0.0041** ES = 1.38	0.074 (0.007, 0.142) ***p*** **= 0.0318** ES = 1.00	0.008 (− 0.060, 0.076) *p* = 0.8070 ES = 0.11	0.036 (− 0.033, 0.105) *p* = 0.3016 ES = 0.49
**20 mg HTL0018318**						
N-back corr-incorr/total 2	0.070 (0.001, 0.140) ***p*** **= 0.0476** ES = 0.95	0.059 (− 0.010, 0.129) *p* = 0.0947 ES = 0.80	0.081 (0.011, 0.151) ***p*** **= 0.0237** ES = 1.09	0.145 (0.077, 0.213) **p = <.0001** ES = 1.96	0.076 (0.008, 0.145) ***p*** **= 0.0299** ES = 1.03	0.051 (− 0.019, 0.120) *p* = 0.1503 ES = 0.68
**25 mg HTL0018318**						
*N*-back corr-incorr/total 2	0.027 (− 0.042, 0.097) *p* = 0.4337 ES = 0.37	0.033 (− 0.038, 0.103) *p* = 0.3640 ES = 0.44	0.073 (0.002, 0.144) ***p*** **= 0.0436** ES = 0.99	0.109 (0.041, 0.177) ***p*** **= 0.0020** ES = 1.47	0.029 (− 0.042, 0.099) *p* = 0.4243 ES = 0.38	0.027 (− 0.047, 0.101) *p* = 0.4690 ES = 0.37
**(20 + 35 mg) HTL0018318**						
*N*-back corr-incorr/total 2				0.061 (− 0.030, 0.152) *p* = 0.1846 ES = 0.67	0.030 (− 0.062, 0.122) *p* = 0.5144 ES = 0.33	0.009 (− 0.083, 0.101) *p* = 0.8429 ES = 0.10

Mean estimated difference (95% CI), *p*-value, effect size

#### Milner maze test

Overall, administration of HTL0018318 in both younger adult and elderly subjects was associated with a reduction in total exploratory errors and total moves (on immediate, reversed and delayed conditions) on the Milner maze test (MMT). There was an overall significant treatment effect of HTL0018318 (including dose level 15 mg, 20 mg, 25 mg and 35 mg) on the performance of the MMT immediate and reversed condition (MMT immediate exploratory error; MMT immediate total moves; MMT reversed exploratory error; MMT reversed total moves). The data from exploratory errors are reported here. See supplement results section for data on the total moves outcome measure which was generally consistent with the data on exploratory errors for immediate and delayed conditions. Overall, HTL0018318 had no significant or consistent effects on exploratory errors in the Milner Maze delayed condition across doses and days in young and elderly subjects. However, selective effects were observed (see supplementary results).

#### MMT immediate: exploratory errors

HTL0018318 had some significant and consistent effects on exploratory errors in the Milner maze test at the 15 mg 20 mg, 25 mg and 35 mg doses across days, particularly in the elderly subjects. In all observations, a lower number of errors were observed indicating better performance.

**Fig. 7:**
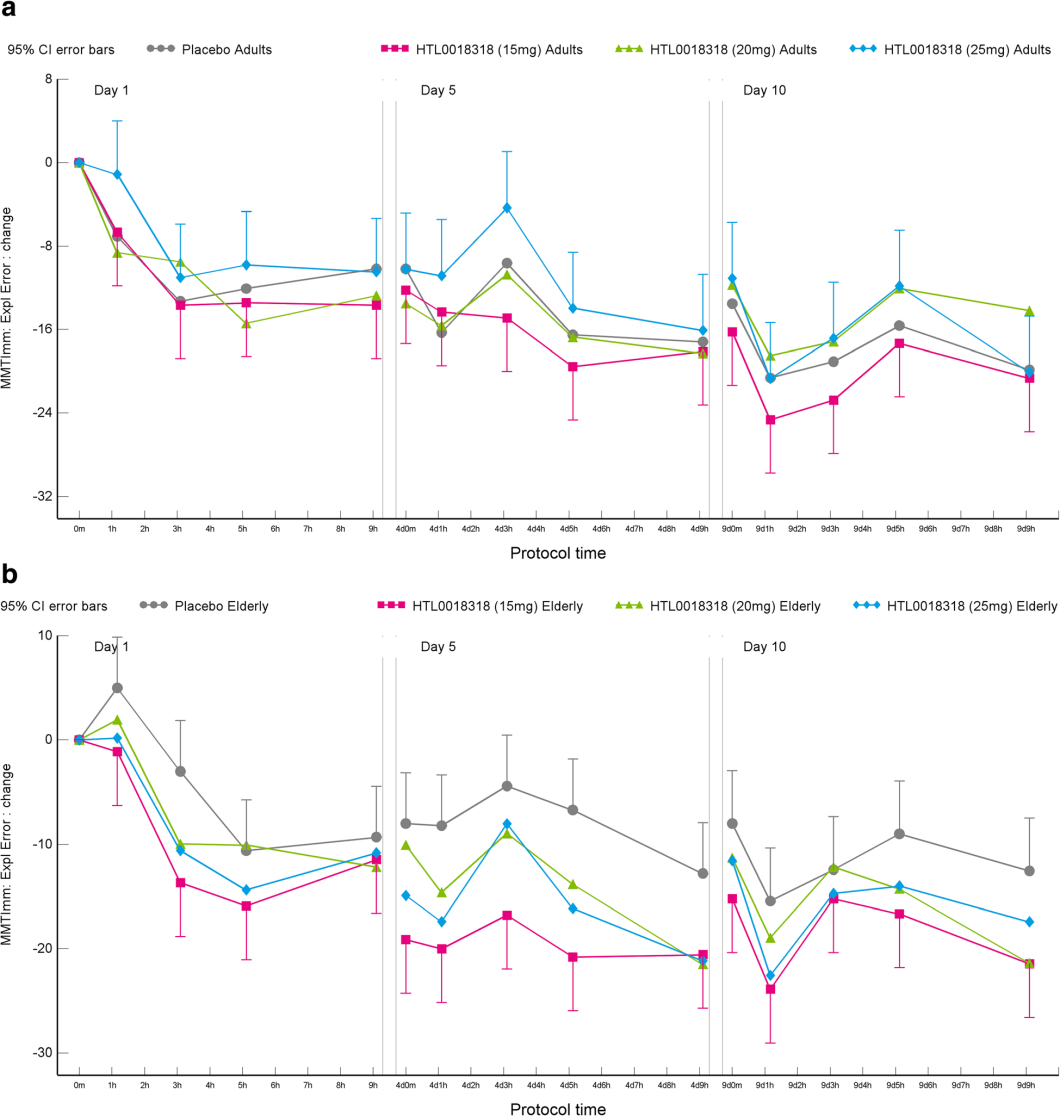
Effects on the MMT immediate condition performance in younger adult (**a**) and elderly subjects (**b**)

Administration of 15 mg HTL0018318 was associated with 3.6 fewer exploratory errors on dosing day 1 (95% CI [− 7.1 to − 0.2], *p* = 0.0380), 6.7 fewer exploratory errors on dosing day 5 (95% CI [− 10.1 to − 3.2], *p* = 0.0002) and 4.8 fewer exploratory errors on dosing day 10 (95% CI [− 8.2 to − 1.3], *p* = 0.0073), compared with placebo. Following 20 mg HTL0018318, 3.6 fewer exploratory errors were observed on dosing day 5 (95% CI [− 7.1 to − 0.1], *p* = 0.0460), compared with placebo. No significant effect was observed after administration of 25 mg HTL0018318 on the number of exploratory errors compared with placebo. Data were analysed separately for younger adult and elderly subjects. These are presented in Table 4 (HTL0018318 compared to placebo, results expressed in exploratory errors) and Fig. 7.

**Table 4:** Effects of HTL0018318 on the performance of the Milner maze test immediate condition

	Younger adults			Elderly		
Parameter	Day 1	Day 5	Day 10	Day 1	Day 5	Day 10
**15 mg HTL0018318**						
MMTImm: Expl Error	− 1.2 (− 6.1, 3.7) *p* = 0.6303 ES = 0.23	− 1.8 (− 6.7, 3.1) *p* = 0.4678 ES = 0.34	− 2.6 (− 7.5, 2.4) *p* = 0.3050 ES = 0.49	− 6.1 (− 10.8, − 1.3) ***p*** **= 0.0133** ES = 1.15	− 11.5 (− 16.3, − 6.7) **p = <.0001** ES = 2.19	− 7.0 (− 11.8, − 2.1) ***p*** **= 0.0052** ES = 1.32
**20 mg HTL0018318**						
MMTImm: Expl Error	− 0.9 (− 5.9, 4.1) *p* = 0.7204 ES = 0.17	− 0.4 (− 5.4, 4.6) *p* = 0.8637 ES = 0.08	3.3 (− 1.7, 8.3) *p* = 0.1930 ES = 0.63	− 3.1 (− 7.9, 1.7) *p* = 0.2061 ES = 0.59	− 6.7 (− 11.5, − 1.9) ***p*** **= 0.0066** ES = 1.28	− 4.3 (− 9.2, 0.5) *p* = 0.0802 ES = 0.83
**25 mg HTL0018318**						
MMTImm: Expl Error	2.6 (− 2.4, 7.5) *p* = 0.3047 ES = 0.49	3.6 (− 1.4, 8.6) *p* = 0.1565 ES = 0.69	1.4 (− 3.6, 6.4) *p* = 0.5720 ES = 0.27	− 4.4 (− 9.3, 0.4) *p* = 0.0718 ES = 0.84	− 7.7 (− 12.6, − 2.7) ***p*** **= 0.0025** ES = 1.46	− 4.8 (− 9.9, 0.3) *p* = 0.0632 ES = 0.92
**(20 + 35 mg) HTL0018318**						
MMTImm: Expl Error				−8.9 (−15.7, − 2.1) ***p*** **= 0.0118** ES = 1.32	− 8.2 (− 15.0, − 1.4) ***p*** **= 0.0196** ES = 1.22	− 1.6 (− 8.4, 5.2) *p* = 0.6317 ES = 0.24

Mean estimated difference (95% CI), *p*-value, effect size

#### MMT reversed: exploratory errors

**Fig. 8:**
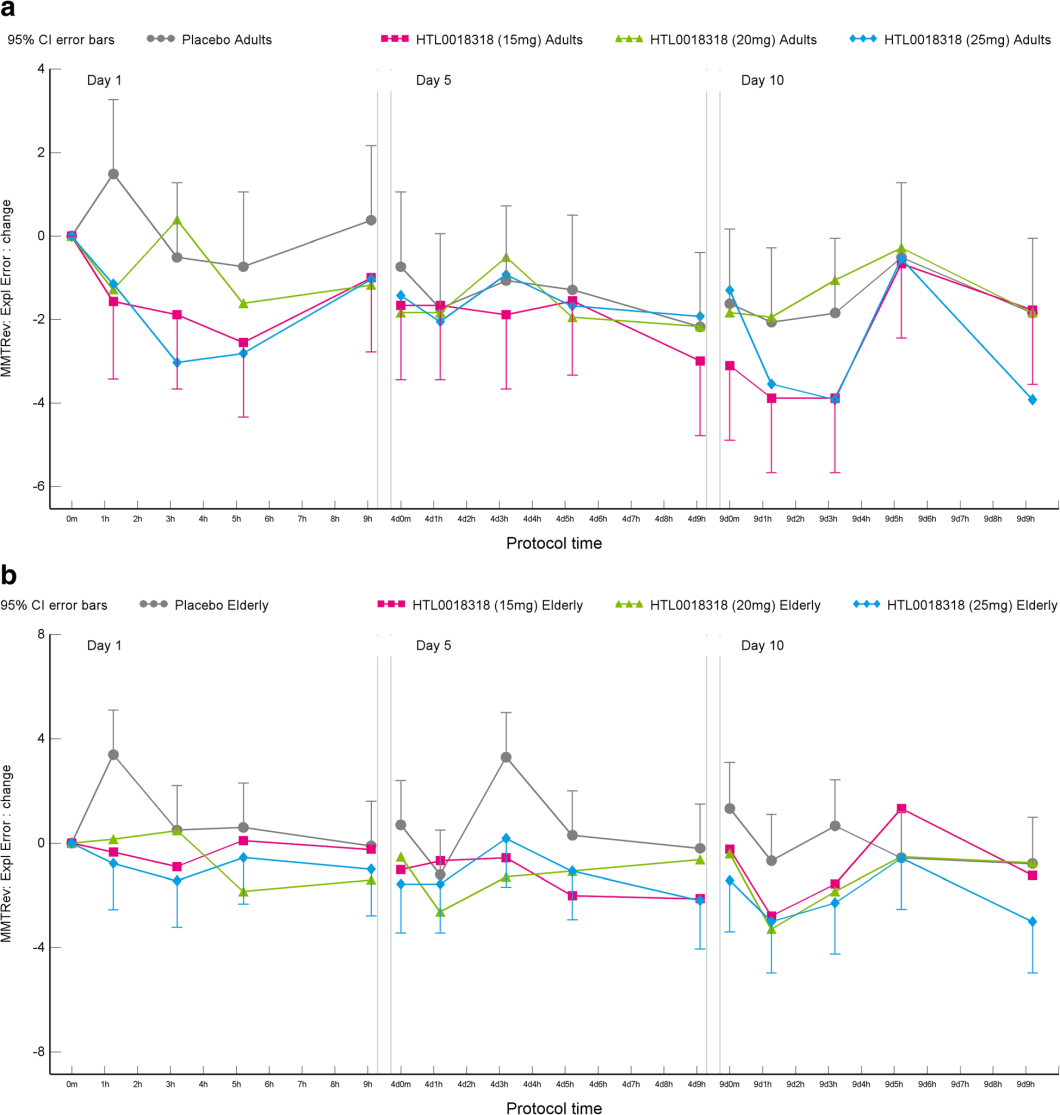
Effects on the MMT reversed condition performance in younger adults (**a**) and elderly subjects (**b**)

Overall, HTL0018318 had some significant and consistent effects on exploratory errors in the Milner maze test at the 15 mg 20 mg, 25 mg and 35 mg doses across days, in younger adult and elderly subjects. In all observations, a lower number of errors were observed indicating better performance. Administration of 15 mg HTL0018318 was associated with 1.7 fewer exploratory errors on dosing day 1 (95% CI [− 2.9 to −0.4], *p* = 0.0086), compared with placebo. After administration of 20 mg HTL0018318, 1.4 fewer exploratory errors were observed on dosing day 1 (95% CI [− 2.7 to −0.2], *p* = 0.0275), compared with placebo. Administration of 25 mg HTL0018318 was associated with 2.1 fewer exploratory errors on dosing day 1 (95% CI [− 3.3 to −0.9], *p* = 0.0011) and 1.7 fewer exploratory errors on dosing day 10 (95% CI [− 2.9 to −0.4], *p* = 0.0119), compared with placebo. The MMT reversed condition data was analysed separately for younger adult and elderly subjects. This is presented in Table 5 (HTL0018318 compared to placebo, results expressed in exploratory errors) and Fig. 8.

**Table 5:** Effects of HTL0018318 on the performance of the Milner maze test reversed condition

	Younger adults			Elderly		
Parameter	Day 1	Day 5	Day 10	Day 1	Day 5	Day 10
**15 mg HTL0018318**						
MMTRev: Expl Error	−1.9 (− 3.7, − 0.1) ***p*** **= 0.0359** ES = 1.00	− 0.5 (− 2.2, 1.3) *p* = 0.6108 ES = 0.24	− 1.0 (− 2.8, 0.8) *p* = 0.2737 ES = 0.52	− 1.4 (− 3.2, 0.3) *p* = 0.1011 ES = 0.76	− 1.9 (− 3.6, − 0.2) ***p*** **= 0.0322** ES = 1.00	− 0.7 (− 2.5, 1.0) *p* = 0.4115 ES = 0.38
**20 mg HTL0018318**						
MMTRev: Expl Error	−1.1 (− 2.9, 0.7) *p* = 0.2347 ES = 0.57	− 0.0 (− 1.8, 1.7) *p* = 0.9592 ES = 0.02	0.3 (− 1.5, 2.1) *p* = 0.7500 ES = 0.15	− 1.8 (− 3.5, − 0.0) ***p*** **= 0.0487** ES = 0.92	− 2.0 (− 3.7, − 0.2) ***p*** **= 0.0284** ES = 1.03	− 1.3 (− 3.0, 0.5) *p* = 0.1593 ES = 0.66
**25 mg HTL0018318**						
MMTRev: Expl Error	−2.2 (− 3.9, − 0.4) ***p*** **= 0.0174** ES = 1.14	− 0.1 (− 1.9, 1.7) *p* = 0.9334 ES = 0.04	− 1.4 (− 3.2, 0.4) *p* = 0.1219 ES = 0.75	− 2.0 (− 3.8, − 0.3) ***p*** **= 0.0217** ES = 1.08	− 1.7 (− 3.5, 0.1) *p* = 0.0577 ES = 0.90	− 1.9 (− 3.7, − 0.1) ***p*** **= 0.0431** ES = 0.99
**(20 + 35 mg) HTL0018318**						
MMTRev: Expl Error				−2.9 (−5.7, − 0.1) ***p*** **= 0.0457** ES = 1.05	− 3.3 (− 6.1, − 0.5) ***p*** **= 0.0230** ES = 1.20	− 2.6 (− 5.4, 0.2) p = 0.0718 ES = 0.93

Mean estimated difference (95% CI), *p*-value, effect size

#### EEG/ERPs

In general, HTL0018318 had no consistent effects on EEG power. For several EEG bands, some statistically significant effects in subjects treated with HTL0018318 compared with placebo were observed; however, these were not consistent across treatment, electrode position or days of treatment. Similarly, there was no consistent effect of HTL0018318 on P300 amplitude or latency, although a significant improvement in P300 amplitude was noted with the 20 mg dose in the elderly on dosing day 1 (mean difference of 3.670 μ V, 95% CI [0.554–6.786], *p* = 0.0222).

#### Other pharmacodynamic biomarkers: blood pressure and pulse rate

Systolic and diastolic blood pressure was not consistently higher or lower in the subjects treated with HTL0018318 than in the placebo subjects. However, some doses of HTL0018318 did demonstrate statistically significant differences versus placebo at some time points. The magnitude and direction of change in blood pressure following HTL008318 treatment was similar for younger adult and elderly subjects. The statistically significant differences in blood pressure on dosing day 1 were relative increases and on dosing day 5 and 10 were relative decreases compared to placebo. This pattern was consistent for supine and standing systolic blood pressure and diastolic blood pressure, but with more significant effects being noted on diastolic blood pressure than on systolic blood pressure.

**Fig. 9:**
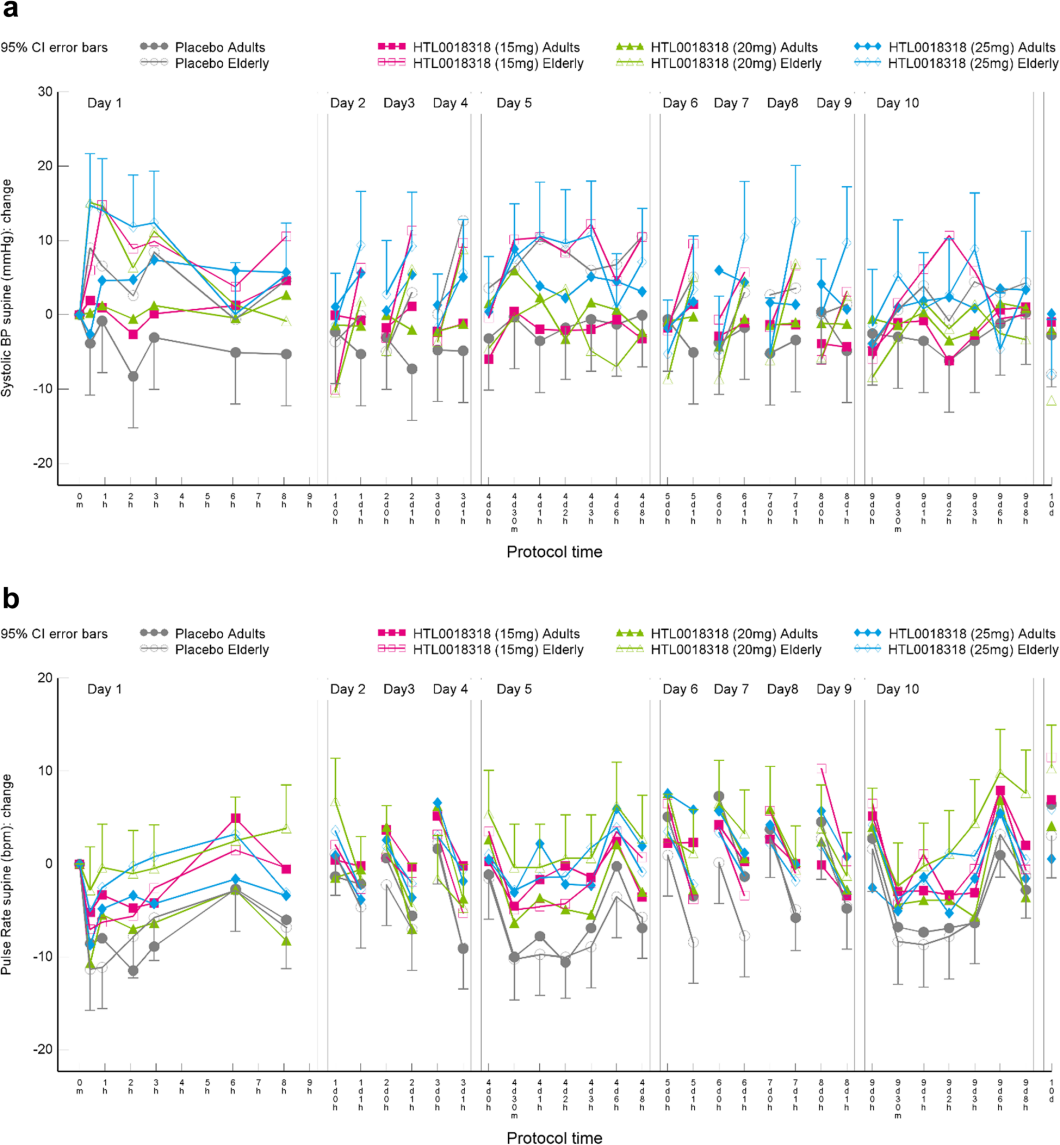
Effects on systolic blood pressure (**a**) and pulse rate (**b**)

Mean systolic blood pressure increased up to 8.7 mmHg (95% CI [1.6, 15.8], *p* = 0.0116, dosing day 1, 25 mg in younger adults) and in mean diastolic blood pressure up to 7.0 mmHg (95% CI [2.4, 11.7], *p* = 0.0036, dosing day 1, 15 mg in elderly subjects) (Fig. 9a and b, results shown as estimate of the change from baseline.). No evidence of a dose response was observed.

There was a statistically significantly higher pulse rate for the majority of treatment groups versus placebo across duration of dosing, particularly in supine pulse rate and in elderly subjects. The maximum increase in mean supine pulse rate was 10 bpm (95% CI [4.7–15.3], *p* = 0.0007, dosing day 5 of 35 mg regimen by up-titration). Similar results were observed in standing pulse rate. See supplement results section for data on systolic and diastolic blood pressure and pulse rate.

## Discussion

We previously reported the safety and tolerability of HTL0018318 following ascending doses in healthy subjects [21]. In this study, we report the safety and tolerability of HTL0018318 following multiple ascending dosing over 10 days in healthy younger adult and elderly subjects. We also report exploratory PD effects on biomarkers of cognitive function. Overall, HTL0018318 was generally well tolerated at the doses tested and there was some evidence for pro-cognitive effects, particularly on tests of short term (working) memory and learning.

Systemic exposure of HTL0018318 showed dose-proportional increases and reproducible PK in the 15–35 mg dose range. The plasma concentrations of HTL0018318 reached a maximum typically 1–2 h post-dose and the apparent half-life was approximately 16 h in younger adult subjects and 14 h in elderly subjects. Elimination of unchanged drug in urine was a major pathway with renal clearance being similar to the age-adjusted glomerular filtration rate. PK characteristics were expected based on the results of the SAD study [21]. Overall, these data suggest that HTL0018318 has a PK profile consistent with a once daily regimen with no clear PK differences between healthy younger adult and elderly subjects.

Multiple doses of HTL0018318 up to 25 mg were well-tolerated and associated with mild and moderate treatment-related cholinergically mediated AEs (reported subjectively) in healthy younger adult and elderly subjects. The highest dose level of 35 mg tested in 2 participants without an up-titration period was not tolerated by elderly subjects; however, this dose was generally well tolerated with the dose titration regimen. In the SAD study, the severity of the AEs was lower, although in the SAD study 3 of the 9 elderly subjects dosed with 35 mg had an increase in blood pressure and more cholinergically mediated AEs compared with other dose levels, suggesting a lower tolerability at this high dose.

Clinically relevant hypertension (an increase of > 40% compared to the baseline measurement or a blood pressure > 180/115 mmHg) occurred in 3 elderly subject following 20 mg, 25 mg or 35 mg without up-titration, which is comparable to the 5 (out of 57) elderly subjects presenting with increased blood pressure in the SAD study [21]. The observed increases in systolic blood pressure of up to 4.6 mmHg in the elderly and up to 8.7 mmHg in the younger adult subjects in the current study and increases up to 11.9 mmHg in the SAD study were both modest increases and showed no dose-dependency. The mean supine pulse rate was significantly higher in the majority of treatment groups in the current study (up to 9.6 bpm in the elderly and up to 7.5 bpm in the younger adult subjects) and some evidence for dose dependency. However, it should be noted that there was a reduction in pulse rate post-dose in the placebo treated participants and therefore the higher pulse rate in the HTL0018318 groups demonstrated less of a reduction (relative to placebo) rather than an increase in pulse rate from baseline with HTL0018318 treatment. While the exact mechanisms associated with the blood pressure and pulse rate changes are not known, it is possible that this may be related to M_1_-mediated modulation of postganglionic sympathetic neurons that innervate the heart [29]. Additionally, there appeared to be a decrease of the cholinergic side effects following repeated dosing of HTL0018318. For example, the increases in blood pressure seen on dosing day 1 attenuated over time (see Fig. 9), suggesting that there may be tolerance to the blood pressure increases with repeat dosing. This phenomenon probably contributes to the better tolerance of 35 mg HTL0018318 preceded by the up-titration period compared to 35 mg HTL0018318 without up-titration period.

Central PD effects were assessed with a range of cognitive tasks probing psychomotor function/attention, working memory and learning as well as electrophysiological biomarkers including P300, a marker of attention and working memory updating. In general, there were no consistent effects on the electrophysiological biomarkers, although this is likely to have been due to poor quality of data and contamination of the P300 data due to a voltage from the trigger pulses leading to high variability. Furthermore, many data sets had to be partially or fully removed due to the artefact (or missing data). This reduced the usable sample considerably reducing the statistical power of the analysis. Hence, these data should be interpreted with caution when interpreting central PD effects measured with EEG/ERP biomarkers including P300.

HTL0018318 over 10 days of treatment was associated with improvements in number of cognitive tests including adaptive tracking in elderly subjects (a measure of psychomotor function and sustained attention), the *n*-back test in both younger adults and elderly subjects (a measure of working memory) and the MMT in elderly subjects (a measure of learning and memory). Overall, HTL0018318 had more consistent effects across cognitive domains in the elderly compared to younger adults, although the study was not adequately powered to investigate differential effects between the two groups. The magnitude of effects on adaptive tracking (i.e. 3.6%-point improvement) was comparable to that previously reported with Donepezil (10 mg) in healthy subjects [23] but were only observed at the 20 mg dose and only on dosing day 1 in elderly subjects suggesting that effects on psychomotor speed and sustained attention were not robust and consistently modulated by M_1_ receptor modulation with HTL0018318. This is consistent with the lack of effects we previously reported with the M_1_ agonist HTL0009936 on adaptive tracking performance . It is possible that cholinergic and M_1_ receptor modulation of attentional processing may depend on “attentional effort” or activation of attentional systems by motivation, particularly in the face of challenges such as distractors where a high level of attentional control is needed [30]. In this context, the adaptive tracking task may have not been challenging enough to require sufficient attentional effort for M_1_ activation to modulate performance. The effects on tests of memory (*n*-back and MMT) were however more consistent in younger adult and elderly subjects across doses and over the 10 days of treatment with clinically relevant effects of moderate to large effect sizes. These effects in healthy normal subjects (presumably with minimal cholinergic dysfunction) are encouraging and may suggest M_1_ receptor modulation may have significant effects on learning and memory in disorders of cholinergic dysfunction such as AD and other dementias. The *n*-back test is a working memory test associated with prefrontal function [31, 32], while the MMT is a learning and memory test associated with hippocampal function [33]. Both the prefrontal cortex and hippocampus are areas rich in muscarinic M_1_ receptors [13, 34]. The sensitivity of these tests to muscarinic (and M_1_) receptor modulation is supported by previous studies with the non-selective muscarinic antagonist scopolamine and the M_1_ antagonist biperiden which have been shown to impair performance on tests comparable to the *n*-back test (45, 46) and MMT (47). The findings of the current study demonstrating positive effects of HTL0018318 on tests of short-term memory and learning are also consistent with the pre-clinical [35, 36] and clinical studies [16, 37] that have similarly shown improvements tests of learning and memory with selective M_1_ receptor agonists. These findings, while preliminary, provide encouraging data in support of the development of HTL0018318 for cognitive dysfunction in AD and other dementias.

The effects of HTL0018318 was also examined on other PD markers including saliva production, LSEQ, pupil size or VAS scores, but overall no significant changes were observed (table in result supplement). While our data showed no effects on saliva production, hypersalivation was observed in other studies investigating other less selective M_1_ mAChR agonists [16, 38–40] and could be explained by their relatively small effects on the M_3_ receptors [40]. The observation in the current study confirms the selectivity of HTL0018318 for muscarinic M_1_ receptors.

### Limitations

There are some limitations of the study that warrant discussion. This study was primarily a safety and tolerability study and the PD measurements were exploratory. As such, these data need to be interpreted with caution, given the small sample size and the lack of power in the study to detect pro-cognitive effects of small to moderate magnitude. While effect sizes were calculated to nuance the PD results calculated by the statistic model (table in result supplement), it is possible that the small sample size could over- or underestimate the pro-cognitive effects of HTL0018318. As discussed above, the EEG/ERP were of poor data quality driven by a voltage noise from the trigger pulses leading to high variability and significant loss of data. Hence, no definite conclusions can be made with regard to the absence of effects of HTL0018318 on the EEG and ERP biomarkers of cognitive function.

## Conclusions

In conclusion, HTL0018318 was generally well-tolerated in multiple doses up to 25 mg/day and dosed up to 10 days (in adult and elderly subjects) or up to 15 days according to a titration regimen of 20 mg/day for 5 days followed by 35 mg/day for 10 days in elderly subjects. The multiple dose PK of HTL0018318 were well-characterised. Treatment-related AEs including cholinergically mediated AEs were mild and transient. Modest changes in blood pressure were observed after the first dose administration, which returned to normal after multiple doses. Consistent and pro-cognitive effects of moderate to large magnitude on short-term memory and learning were demonstrated across the dose range over the 10 days of treatment providing encouraging data in support of the development of HTL0018318 for cognitive dysfunction in dementias.

## Supplementary Information

**Additional file 1.**

## Data Availability

Availability of data and materials: The datasets generated during and/or analysed during the current study are filed in EudraCT and are not publicly available [in accordance with the regulations for Phase 1 data]. Further information is available from the corresponding author on reasonable request. Code availability: The model code is available upon request.

## Abbreviations

%CV: Coefficient of variation
ACh: Acetylcholine
AD: Alzheimer’s disease
Ae%: Percentage of dose excreted renally as unchanged drug
AEs: Adverse events
ANCOVA: Analysis of covariance
AUC: Area under the plasma-concentration-time curve
AUC_0-inf_: AUC from zero to infinity
AUC_0-tau_: AUC from zero to the end of the dose interval
AUC_last_: AUC from zero to the last measurement
BMI: Body mass index
ChEIs: Acetylcholinesterase inhibitors
CIs: Confidence intervals
CL/F: Apparent oral clearance
CLr: Renal clearance
C_max_: Maximum observed plasma concentration
C_min_: The minimum concentration
CNS: Central nervous system
DLB: Dementia with Lewy bodies
ECG: Electrocardiogram
EEG: Electroencephalogram
ERP: Event-related potentials
LSEQ: Leeds Sleep Evaluation Questionnaire
LSMs: Least-square means
mAChR: Muscarinic acetylcholine receptor
MMN: Mismatch negativity
MMT: Milner maze test
PD: Pharmacodynamics
PFT: Pulmonary function tests
PK: Pharmacokinetics
SAD: Single ascending dose
t_1/2_: Apparent elimination half-life
t_last_: Time of the last quantifiable concentration
t_max_: Time to C_max_
VAS: Visual analogue scale
Vz/F: Apparent volume of distribution
