# Supplementary material for: Safety, pharmacokinetics and exploratory pro-cognitive effects of HTL0018318, a selective M1 receptor agonist, in healthy younger adult and elderly subjects: a multiple ascending dose study
Source: Alzheimers Res Ther. 2021 Apr 21;13:87. doi: 10.1186/s13195-021-00816-5 (PMC8061066; doi:10.1186/s13195-021-00816-5)
Supplement: Supplementary file 1 — Additional file 1. [file 13195_2021_816_MOESM1_ESM.docx]

1. Methods supplement

**Description pharmacodynamic measurements**

Adaptive tracking

A circle of known dimensions moves randomly about a screen. The subject had to try to keep a dot inside the moving circle by operating a joystick. If this effort was successful, the speed of the moving circle increased. Conversely, the velocity decreased if the subject could not maintain the dot inside the circle. Before study participation, subjects performed three training sessions. The average performance and the standard deviation of scores over a 3.5-minute period were used for analysis. This 3.5-minute period is including a run in time of 0.5 minute, in this run in time the data was not recorded. The adaptive tracking test used was developed by Hobbs & Strutt, according to specifications of Borland and Nicholson (1-3). The performance was expressed in percentage of time that the circle was correctly tracked. This test was used to measure sustained attention.

Milner Maze test

The Milner Maze Test (MMT) is a computerised version of the Milner Maze (1965). This is a spatial working memory test which was developed Milner and based on an early hidden maze task developed by Barker (1931) and extended by Milner (1965). Subjects had to complete a maze by using trial and error learning to locate a 28-step pathway that was hidden beneath a 10×10 grid of tiles. Subjects attempted to find the same pathway on successive trials. There are three types of trials in the MMT: Immediate for imprinting (five times the same path version) and Delayed (the same path once) and Reversed (the same path once in reversed direction) for memory function. Spatial working memory function can be inferred from the accuracy (exploratory errors, total moves) and speed (exploratory time) of decisions across these trials (4-6).

N-back task

Letters were presented consecutively on the screen with a speed of 30 letters per minute. In the 0-back condition subjects had to indicate whether the letter on the screen was an ‘x’. In the 1-back condition, subjects indicated whether the letter seen was identical to the previous letter. In the 2-back condition, subjects were asked to indicate whether the letter was identical to two letters before the letter seen. Performance was expressed as ‘correct-incorrect/total answers’ representing accuracy and as reaction time (7-9).

Pupillometry

A sharp picture of the eyes was taken using a digital camera (Canon EOS 1100D) and a flash. The diameters of the pupil and the iris were determined in the number of pixels used horizontally. For each eye the pupil / iris ratio was calculated as a measure of pupil size (10, 11).

Resting EEG

An 8-minute resting EEG was performed. During this measurement, subjects were resting alternating with their eyes closed and their eyes opened for four minutes on each condition. The procedure used was the 10-20 system of the International Federation of Societies for ElectroEncephaloGraphy and Clinical Neurophysiology (IFSECN), which divided the head into segments of 10% or 20% to determine the sites for electrode placement. The EEG was recorded using the Trackit™ EEG (Lifelines Ltd., UK) to record while the test was being executed and acquired using the Trackit™ Software (Lifelines Ltd., UK). All EEGs were done with the subject being in supine position. Data collection and analysis were performed using customized CED and Spike2 for Windows software (Cambridge Electronics Design, Cambridge, UK) (12). The analyzed leads were Fz-Cz, Pz-O1 and Pz-O2 for each frequency band (delta, theta, alpha, beta and gamma).

ERP P300

An auditory oddball paradigm was used to elicit the P300 component. Auditory stimuli were presented to subjects via a headphone (70dB SPL). Infrequent tone of 500 Hz were presented randomly among frequent tones of 1000 Hz. The probabilities of the frequent and infrequent tones were 80% and 20%, respectively. In total 400 auditory stimuli were presented. The tone duration was 50 ms and the interstimulus interval was uniformly varied between 800 and 1400 ms. Subjects were instructed to promptly press the spacebar after a target tone (13). The amplitude and latency of the P300 component were assessed.

ERP Mismatch negativity

The MMN component was also elicited using the Oddball paradigm sequence. This stimulus sequence involved the same series of lower (500 Hz) and higher (1000 Hz) pitched pure tones but were presented at a rate of 1 tone per 0.6 sec. The tones were 50 ms in duration, at 70 dB, and semi-randomly interspersed. The lower pitched tone was the target or infrequent event. Across the series of 750 tones, the lower pitched tone occurred at a probability of 0.2, the higher tone occurred at the complementary probability of 0.8. Subjects were instructed to ignore both frequent and infrequent tones while watching a silent neutral movie (14, 15). The amplitude and the latency of the MMN component were assessed.

Visual analogue scale Bond and Lader

The VAS according to Bond and Lader, consists of 3 composite scores derived from the originally VASs described by Norris, assessing alertness, mood and calmness. The VAS consists of a 100-mm line anchored at each end by words descriptive of opposing statements. On this linear scale, the subject indicated his/her mood state at the moment by placing a mark between 2 statements (2, 16).

Visual analogue scale Nausea

A 100‐mm line marked “no nausea” at the left‐hand end and “unbearable nausea” at the right‐hand end, was used to evaluate subjective nausea (5).

Leeds Sleep Evaluation Questionnaire

This questionnaire is a 10-item, subjective, self-report measure, consisting of 100-mm line analogue questions and is designed to assess changes in sleep quality over the course of a psychopharmacological treatment intervention. The scale evaluated four domains: ease of initiating sleep, quality of sleep, ease of waking, and behaviour following wakefulness (17).

1. Results supplement

## Summary PD tables younger adults/elderly 15-20-25 mg HTL001818

|  | | **Contrasts** | | | | | |
| --- | --- | --- | --- | --- | --- | --- | --- |
|  | | **Adults** | | | **Elderly** | | |
| **Parameter** | **Treatment P-value** | **Day 1 HTL0018318 (15mg) Placebo** | **Day 5 HTL0018318 (15mg) Placebo** | **Day 10 HTL0018318 (15mg) Placebo** | **Day 1 HTL0018318 (15mg) Placebo** | **Day 5 HTL0018318 (15mg) Placebo** | **Day 10 HTL0018318 (15mg) Placebo** |
| Saliva (g) | 0.2181 | -0.702 ( -1.788, 0.384)  p=0.2020  ES=0.61 | -0.136 ( -1.388, 1.116)  p=0.8303  ES=0.12 | 0.222 ( -0.864, 1.309)  p=0.6849  ES=0.19 | -0.446 ( -1.500, 0.609)  p=0.4027  ES=0.39 | -0.280 ( -1.496, 0.936)  p=0.6492  ES=0.24 | -0.228 ( -1.286, 0.831)  p=0.6696  ES=0.20 |
| LSEQ: Getting to sleep (mm) | 0.4138 | -0.90 ( -3.56, 1.77)  p=0.5097  ES=0.45 | -0.04 ( -2.71, 2.62)  p=0.9744  ES=0.02 | 0.14 ( -2.53, 2.81)  p=0.9169  ES=0.07 | 2.63 ( -0.01, 5.27)  p=0.0509  ES=1.33 | 1.36 ( -1.28, 4.00)  p=0.3116  ES=0.69 | 0.41 ( -2.28, 3.11)  p=0.7632  ES=0.21 |
| LSEQ: Quality of sleep (mm) | 0.6614 | -4.65 ( -10.80, 1.49)  p=0.1369  ES=0.97 | 4.57 ( -1.57, 10.71)  p=0.1444  ES=0.95 | 5.29 ( -0.85, 11.43)  p=0.0911  ES=1.10 | -1.11 ( -7.12, 4.90)  p=0.7160  ES=0.23 | -0.98 ( -6.99, 5.02)  p=0.7475  ES=0.20 | 3.11 ( -3.01, 9.22)  p=0.3178  ES=0.65 |
| LSEQ: Awake following sleep (mm) | 0.6424 | 0.59 ( -4.97, 6.15)  p=0.8351  ES=0.12 | 0.92 ( -4.64, 6.48)  p=0.7442  ES=0.19 | 0.36 ( -5.20, 5.93)  p=0.8971  ES=0.07 | 0.57 ( -5.08, 6.23)  p=0.8415  ES=0.12 | -1.12 ( -6.77, 4.54)  p=0.6974  ES=0.23 | 0.68 ( -5.04, 6.40)  p=0.8149  ES=0.14 |
| LSEQ: Behaviour after wake (mm) | 0.2364 | -5.77 ( -11.73, 0.20)  p=0.0581  ES=1.15 | 1.09 ( -4.88, 7.05)  p=0.7200  ES=0.22 | 0.09 ( -5.88, 6.05)  p=0.9774  ES=0.02 | 5.47 ( -0.36, 11.30)  p=0.0660  ES=1.09 | 0.24 ( -5.59, 6.07)  p=0.9357  ES=0.05 | -1.37 ( -7.29, 4.55)  p=0.6481  ES=0.27 |
| Systolic BP supine (mmHg) | 0.3170 | 5.4 ( -1.6, 12.5)  p=0.1305  ES=0.69 | -0.3 ( -7.4, 6.8)  p=0.9312  ES=0.04 | 1.3 ( -5.7, 8.4)  p=0.7075  ES=0.17 | 3.9 ( -3.2, 11.0)  p=0.2804  ES=0.49 | 1.3 ( -5.8, 8.4)  p=0.7167  ES=0.16 | 1.4 ( -5.8, 8.5)  p=0.7007  ES=0.18 |
| Diastolic BP supine (mmHg) | 0.0135 | 6.7 ( 2.0, 11.5)  p=0.0063  ES=1.26 | -0.6 ( -5.4, 4.2)  p=0.8065  ES=0.11 | -0.2 ( -5.0, 4.5)  p=0.9208  ES=0.04 | 7.0 ( 2.4, 11.7)  p=0.0036  ES=1.32 | 0.6 ( -4.0, 5.3)  p=0.7837  ES=0.12 | 1.3 ( -3.4, 6.0)  p=0.5867  ES=0.24 |
| Pulse Rate supine (bpm) | 0.0112 | 5.4 ( 0.8, 10.1)  p=0.0219  ES=1.05 | 5.6 ( 1.0, 10.2)  p=0.0182  ES=1.08 | 4.5 ( -0.1, 9.1)  p=0.0570  ES=0.87 | 4.2 ( -0.3, 8.7)  p=0.0655  ES=0.82 | 6.1 ( 1.6, 10.6)  p=0.0085  ES=1.18 | 4.8 ( 0.3, 9.3)  p=0.0385  ES=0.93 |
| Systolic BP standing (mmHg) | 0.6207 | 5.0 ( -2.7, 12.7)  p=0.2038  ES=0.57 | -2.6 ( -10.4, 5.1)  p=0.4978  ES=0.31 | 2.1 ( -5.6, 9.9)  p=0.5815  ES=0.25 | 2.7 ( -4.9, 10.4)  p=0.4801  ES=0.32 | 1.1 ( -6.5, 8.7)  p=0.7758  ES=0.13 | 0.1 ( -7.6, 7.8)  p=0.9765  ES=0.01 |
| Diastolic BP standing (mmHg) | 0.1009 | 7.3 ( 1.7, 12.8)  p=0.0113  ES=1.17 | 0.1 ( -5.5, 5.7)  p=0.9788  ES=0.01 | 0.7 ( -4.9, 6.3)  p=0.8093  ES=0.11 | 7.1 ( 1.6, 12.5)  p=0.0119  ES=1.14 | -0.5 ( -6.0, 4.9)  p=0.8489  ES=0.08 | -0.9 ( -6.4, 4.6)  p=0.7510  ES=0.14 |
| Pulse Rate standing (bpm) | 0.2866 | 0.5 ( -5.1, 6.0)  p=0.8624  ES=0.08 | 2.1 ( -3.4, 7.6)  p=0.4541  ES=0.34 | 0.3 ( -5.2, 5.9)  p=0.9055  ES=0.05 | 4.8 ( -0.6, 10.3)  p=0.0824  ES=0.77 | 5.8 ( 0.4, 11.2)  p=0.0360  ES=0.93 | 3.5 ( -2.0, 8.9)  p=0.2128  ES=0.55 |
| Systolic BP sup-sta (mmHg) | 0.7415 | -0.0 ( -5.5, 5.4)  p=0.9987  ES=0.00 | 1.9 ( -3.6, 7.3)  p=0.4994  ES=0.29 | -1.1 ( -6.5, 4.4)  p=0.6990  ES=0.17 | 0.4 ( -4.9, 5.7)  p=0.8846  ES=0.06 | -0.4 ( -5.7, 4.9)  p=0.8882  ES=0.06 | 0.7 ( -4.7, 6.0)  p=0.8075  ES=0.10 |
| Diastolic BP sup-sta (mmHg) | 0.9738 | -0.6 ( -4.0, 2.9)  p=0.7517  ES=0.14 | -0.7 ( -4.1, 2.7)  p=0.6881  ES=0.17 | -0.9 ( -4.3, 2.5)  p=0.6100  ES=0.22 | 0.2 ( -3.2, 3.5)  p=0.9292  ES=0.04 | 1.5 ( -1.9, 4.8)  p=0.3886  ES=0.36 | 2.4 ( -1.0, 5.8)  p=0.1583  ES=0.60 |
| Pulse Rate sup-sta (bpm) | 0.5454 | 4.2 ( 0.4, 8.1)  p=0.0320  ES=0.94 | 2.7 ( -1.2, 6.6)  p=0.1754  ES=0.59 | 3.5 ( -0.4, 7.4)  p=0.0797  ES=0.77 | -1.0 ( -4.8, 2.8)  p=0.6131  ES=0.22 | 0.0 ( -3.8, 3.8)  p=0.9947  ES=0.00 | 1.1 ( -2.7, 4.9)  p=0.5766  ES=0.24 |
| Track Performance (%) | 0.3407 | -1.418 ( -4.265, 1.428)  p=0.3243  ES=0.47 | -0.392 ( -3.238, 2.454)  p=0.7847  ES=0.13 | -1.416 ( -4.262, 1.430)  p=0.3251  ES=0.47 | 2.322 ( -0.496, 5.139)  p=0.1050  ES=0.77 | 1.214 ( -1.604, 4.031)  p=0.3937  ES=0.40 | 1.684 ( -1.147, 4.515)  p=0.2400  ES=0.56 |
| N-back corr-incorr/total 0 | 0.1986 | 0.014 ( -0.020, 0.047)  p=0.4164  ES=0.40 | 0.038 ( 0.004, 0.072)  p=0.0268  ES=1.10 | 0.004 ( -0.029, 0.038)  p=0.7949  ES=0.13 | 0.014 ( -0.018, 0.046)  p=0.4022  ES=0.39 | 0.020 ( -0.012, 0.052)  p=0.2236  ES=0.57 | 0.001 ( -0.031, 0.034)  p=0.9426  ES=0.03 |
| N-back corr-incorr/total 1 | 0.0605 | 0.053 ( -0.000, 0.106)  p=0.0507  ES=0.95 | 0.060 ( 0.007, 0.113)  p=0.0273  ES=1.07 | 0.033 ( -0.021, 0.086)  p=0.2310  ES=0.59 | 0.052 ( 0.001, 0.103)  p=0.0461  ES=0.93 | -0.018 ( -0.069, 0.033)  p=0.4937  ES=0.32 | -0.005 ( -0.056, 0.047)  p=0.8558  ES=0.09 |
| N-back corr-incorr/total 2 | 0.0024 | 0.079 ( 0.009, 0.148)  p=0.0265  ES=1.06 | 0.074 ( 0.005, 0.143)  p=0.0361  ES=1.00 | 0.102 ( 0.033, 0.172)  p=0.0041  ES=1.38 | 0.074 ( 0.007, 0.142)  p=0.0318  ES=1.00 | 0.008 ( -0.060, 0.076)  p=0.8070  ES=0.11 | 0.036 ( -0.033, 0.105)  p=0.3016  ES=0.49 |
| N-back mean RT 0 back (msec) | 0.7917 | 4.7 ( -38.6, 48.0)  p=0.8289  ES=0.10 | 9.5 ( -33.8, 52.8)  p=0.6624  ES=0.21 | -19.8 ( -63.2, 23.7)  p=0.3691  ES=0.43 | -5.4 ( -48.4, 37.5)  p=0.8026  ES=0.12 | -31.3 ( -74.4, 11.7)  p=0.1515  ES=0.68 | -17.2 ( -60.5, 26.1)  p=0.4313  ES=0.37 |
| N-back mean RT 1 back (msec) | 0.9521 | 10.7 ( -42.1, 63.5)  p=0.6870  ES=0.19 | -9.8 ( -62.6, 43.0)  p=0.7131  ES=0.18 | 14.1 ( -38.9, 67.1)  p=0.5984  ES=0.25 | 5.3 ( -46.2, 56.9)  p=0.8379  ES=0.10 | -22.0 ( -73.6, 29.7)  p=0.4006  ES=0.39 | 23.6 ( -28.4, 75.5)  p=0.3702  ES=0.42 |
| N-back mean RT 2 back (msec) | 0.3380 | -15.9 ( -89.9, 58.0)  p=0.6697  ES=0.20 | -4.8 ( -78.7, 69.2)  p=0.8988  ES=0.06 | -12.9 ( -87.0, 61.3)  p=0.7310  ES=0.16 | -30.3 ( -103.6, 42.9)  p=0.4128  ES=0.38 | -51.7 ( -125.0, 21.6)  p=0.1645  ES=0.65 | -52.4 ( -126.2, 21.4)  p=0.1620  ES=0.66 |
| MMTImm: Expl Error | 0.0082 | -1.2 ( -6.1, 3.7)  p=0.6303  ES=0.23 | -1.8 ( -6.7, 3.1)  p=0.4678  ES=0.34 | -2.6 ( -7.5, 2.4)  p=0.3050  ES=0.49 | -6.1 ( -10.8, -1.3)  p=0.0133  ES=1.15 | -11.5 ( -16.3, -6.7)  p=<.0001  ES=2.19 | -7.0 ( -11.8, -2.1)  p=0.0052  ES=1.32 |
| MMTImm: Total Moves | 0.0192 | -0.9 ( -10.9, 9.2)  p=0.8656  ES=0.08 | -1.8 ( -11.8, 8.2)  p=0.7226  ES=0.17 | -4.0 ( -14.0, 6.0)  p=0.4320  ES=0.37 | -11.4 ( -21.2, -1.7)  p=0.0215  ES=1.07 | -23.4 ( -33.1, -13.7)  p=<.0001  ES=2.19 | -13.5 ( -23.4, -3.6)  p=0.0077  ES=1.26 |
| MMTImm: Expl Time (msec) | 0.9288 | 9470.2 (-7465.2, 26405.6)  p=0.2694  ES=0.53 | 611.7 ( -16324, 17547.1)  p=0.9429  ES=0.03 | 117.5 ( -16818, 17052.9)  p=0.9890  ES=0.01 | -12710 ( -29052, 3633.1)  p=0.1258  ES=0.71 | -3901.7 ( -20244, 12441.0)  p=0.6363  ES=0.22 | 499.5 ( -15954, 16953.3)  p=0.9520  ES=0.03 |
| MMTRev: Expl Error | 0.0366 | -1.9 ( -3.7, -0.1)  p=0.0359  ES=1.00 | -0.5 ( -2.2, 1.3)  p=0.6108  ES=0.24 | -1.0 ( -2.8, 0.8)  p=0.2737  ES=0.52 | -1.4 ( -3.2, 0.3)  p=0.1011  ES=0.76 | -1.9 ( -3.6, -0.2)  p=0.0322  ES=1.00 | -0.7 ( -2.5, 1.0)  p=0.4115  ES=0.38 |
| MMTRev: Total Moves | 0.0251 | -3.7 ( -7.4, -0.1)  p=0.0429  ES=0.97 | -0.9 ( -4.5, 2.7)  p=0.6181  ES=0.24 | -2.0 ( -5.6, 1.6)  p=0.2695  ES=0.52 | -2.9 ( -6.5, 0.6)  p=0.1012  ES=0.76 | -4.5 ( -8.0, -1.0)  p=0.0126  ES=1.17 | -1.7 ( -5.3, 1.9)  p=0.3481  ES=0.44 |
| MMTRev: Expl Time (msec) | 0.2267 | 2879.6 (-1127.0, 6886.3)  p=0.1574  ES=0.67 | 2960.0 (-1033.8, 6953.7)  p=0.1450  ES=0.69 | 887.6 (-3106.1, 4881.3)  p=0.6608  ES=0.21 | -1537.3 (-5460.1, 2385.5)  p=0.4395  ES=0.36 | -487.9 (-4410.7, 3434.9)  p=0.8060  ES=0.11 | 5135.9 ( 1161.4, 9110.5)  p=0.0117  ES=1.20 |
| MMTDel: Expl Error | 0.1938 | -0.2 ( -1.7, 1.2)  p=0.7363  ES=0.16 | -1.8 ( -3.2, -0.3)  p=0.0163  ES=1.15 | -1.2 ( -2.7, 0.2)  p=0.0897  ES=0.81 | -0.3 ( -1.7, 1.2)  p=0.7180  ES=0.17 | -0.5 ( -2.0, 0.9)  p=0.4665  ES=0.35 | -0.1 ( -1.6, 1.3)  p=0.8591  ES=0.09 |
| MMTDel: Total Moves | 0.1404 | -0.5 ( -3.4, 2.4)  p=0.7285  ES=0.16 | -3.4 ( -6.3, -0.5)  p=0.0236  ES=1.08 | -2.4 ( -5.3, 0.5)  p=0.1053  ES=0.77 | -1.1 ( -4.1, 1.9)  p=0.4641  ES=0.35 | -1.4 ( -4.3, 1.6)  p=0.3543  ES=0.44 | -0.3 ( -3.3, 2.6)  p=0.8303  ES=0.10 |
| MMTDel: Expl Time (msec) | 0.6223 | 3511.0 ( -695.4, 7717.3)  p=0.1009  ES=0.78 | -587.8 (-4794.1, 3618.6)  p=0.7822  ES=0.13 | 800.4 (-3406.0, 5006.7)  p=0.7066  ES=0.18 | -2577.6 (-6851.6, 1696.4)  p=0.2344  ES=0.58 | 844.0 (-3402.7, 5090.6)  p=0.6942  ES=0.19 | 3848.4 ( -425.2, 8122.1)  p=0.0771  ES=0.86 |
| Left Pupil/Iris ratio | 0.9442 | -.01188 (-.04553, 0.02176)  p=0.4844  ES=0.35 | -.01435 (-.04800, 0.01930)  p=0.3986  ES=0.42 | -.00881 (-.04246, 0.02484)  p=0.6039  ES=0.26 | 0.01201 (-.01909, 0.04311)  p=0.4448  ES=0.35 | 0.00882 (-.02224, 0.03988)  p=0.5737  ES=0.26 | 0.00616 (-.02512, 0.03744)  p=0.6963  ES=0.18 |
| Right Pupil/Iris ratio | 0.8405 | -.00256 (-.03818, 0.03306)  p=0.8867  ES=0.07 | -.01161 (-.04723, 0.02401)  p=0.5183  ES=0.32 | -.00053 (-.03615, 0.03509)  p=0.9763  ES=0.01 | -.00409 (-.03679, 0.02861)  p=0.8042  ES=0.11 | -.00827 (-.04093, 0.02440)  p=0.6160  ES=0.23 | -.00695 (-.03981, 0.02592)  p=0.6754  ES=0.19 |
| VAS Alertness (mm) | 0.9337 | -1.02 ( -4.38, 2.33)  p=0.5455  ES=0.29 | 1.38 ( -1.97, 4.74)  p=0.4151  ES=0.39 | 0.35 ( -3.01, 3.70)  p=0.8383  ES=0.10 | 1.16 ( -2.12, 4.43)  p=0.4850  ES=0.32 | -0.87 ( -4.15, 2.40)  p=0.5969  ES=0.24 | -2.01 ( -5.30, 1.29)  p=0.2294  ES=0.56 |
| VAS Calmness (mm) | 0.6199 | 2.27 ( -1.72, 6.25)  p=0.2619  ES=0.53 | 1.93 ( -2.05, 5.92)  p=0.3382  ES=0.45 | 2.39 ( -1.60, 6.38)  p=0.2367  ES=0.56 | 0.23 ( -3.67, 4.14)  p=0.9063  ES=0.05 | -1.91 ( -5.82, 1.99)  p=0.3337  ES=0.45 | -3.23 ( -7.17, 0.71)  p=0.1068  ES=0.76 |
| VAS Mood (mm) | 0.6981 | -0.21 ( -3.92, 3.49)  p=0.9092  ES=0.05 | 1.75 ( -1.95, 5.46)  p=0.3490  ES=0.45 | 1.71 ( -1.99, 5.41)  p=0.3613  ES=0.43 | 1.51 ( -2.09, 5.11)  p=0.4059  ES=0.38 | -0.46 ( -4.06, 3.14)  p=0.7987  ES=0.12 | -2.65 ( -6.27, 0.97)  p=0.1494  ES=0.67 |
| VAS Nausea (log(mm)) | 0.3618 | 0.12 ( -0.01, 0.25)  p=0.0606  ES=0.90 | 0.06 ( -0.07, 0.18)  p=0.3808  ES=0.42 | 0.03 ( -0.10, 0.15)  p=0.6589  ES=0.21 | -0.01 ( -0.13, 0.11)  p=0.8749  ES=0.07 | -0.13 ( -0.26, -0.01)  p=0.0337  ES=0.99 | -0.03 ( -0.16, 0.09)  p=0.5865  ES=0.26 |
| EEG Alpha Fz-Cz closed (uV^2/Hz) | 0.8835 | -6.1% (-35.2%, 35.9%)  p=0.7348  ES=0.17 | -0.3% (-31.3%, 44.5%)  p=0.9860  ES=0.01 | 22.2% (-15.7%, 77.2%)  p=0.2863  ES=0.53 | 1.0% (-29.1%, 43.8%)  p=0.9555  ES=0.03 | 5.1% (-26.0%, 49.3%)  p=0.7783  ES=0.13 | -1.6% (-31.1%, 40.5%)  p=0.9295  ES=0.04 |
| EEG Alpha Fz-Cz open (uV^2/Hz) | 0.8005 | -1.8% (-29.6%, 36.9%)  p=0.9117  ES=0.05 | 16.0% (-17.0%, 62.1%)  p=0.3790  ES=0.44 | 32.4% ( -5.4%, 85.2%)  p=0.1003  ES=0.82 | 3.2% (-25.1%, 42.2%)  p=0.8452  ES=0.09 | 16.3% (-15.4%, 60.0%)  p=0.3479  ES=0.44 | 4.7% (-24.2%, 44.7%)  p=0.7779  ES=0.13 |
| EEG Alpha Pz-O1 closed (uV^2/Hz) | 0.6978 | -43.2% (-70.1%, 8.0%)  p=0.0835  ES=0.86 | -25.9% (-61.1%, 41.2%)  p=0.3583  ES=0.46 | -22.0% (-59.1%, 48.6%)  p=0.4448  ES=0.38 | 38.1% (-25.6%, 156.4%)  p=0.3024  ES=0.49 | -25.0% (-59.4%, 38.7%)  p=0.3543  ES=0.44 | -19.1% (-56.6%, 50.7%)  p=0.4988  ES=0.32 |
| EEG Alpha Pz-O1 open (uV^2/Hz) | 0.7428 | -19.6% (-56.0%, 46.9%)  p=0.4743  ES=0.35 | -3.5% (-47.3%, 76.6%)  p=0.9063  ES=0.06 | 0.5% (-45.2%, 84.4%)  p=0.9867  ES=0.01 | 17.8% (-34.4%, 111.5%)  p=0.5795  ES=0.27 | -19.4% (-55.0%, 44.4%)  p=0.4641  ES=0.35 | -31.2% (-61.8%, 24.1%)  p=0.2109  ES=0.61 |
| EEG Alpha Pz-O2 closed (uV^2/Hz) | 0.8168 | -38.4% (-66.9%, 14.8%)  p=0.1255  ES=0.76 | -24.4% (-59.5%, 41.2%)  p=0.3758  ES=0.44 | -31.7% (-63.4%, 27.7%)  p=0.2290  ES=0.60 | 25.7% (-30.8%, 128.6%)  p=0.4479  ES=0.36 | -11.2% (-50.9%, 60.7%)  p=0.6920  ES=0.19 | -5.4% (-48.2%, 72.5%)  p=0.8535  ES=0.09 |
| EEG Alpha Pz-O2 open (uV^2/Hz) | 0.9830 | -9.8% (-50.8%, 65.3%)  p=0.7360  ES=0.17 | 13.7% (-38.1%, 108.8%)  p=0.6746  ES=0.21 | 10.9% (-39.7%, 104.1%)  p=0.7356  ES=0.17 | 15.2% (-35.4%, 105.2%)  p=0.6277  ES=0.23 | -16.1% (-52.8%, 49.0%)  p=0.5443  ES=0.29 | -14.4% (-52.1%, 53.0%)  p=0.5956  ES=0.25 |
| EEG Beta Fz-Cz closed (uV^2/Hz) | 0.8465 | -0.1% (-22.2%, 28.1%)  p=0.9908  ES=0.01 | 9.4% (-14.8%, 40.5%)  p=0.4767  ES=0.35 | 8.1% (-15.9%, 38.8%)  p=0.5395  ES=0.31 | 4.0% (-18.2%, 32.3%)  p=0.7458  ES=0.16 | 11.4% (-12.2%, 41.4%)  p=0.3700  ES=0.43 | 4.7% (-17.8%, 33.3%)  p=0.7078  ES=0.18 |
| EEG Beta Fz-Cz open (uV^2/Hz) | 0.7636 | -8.3% (-28.7%, 18.0%)  p=0.4974  ES=0.34 | 7.6% (-16.5%, 38.6%)  p=0.5685  ES=0.29 | 5.1% (-18.5%, 35.5%)  p=0.7000  ES=0.19 | 6.2% (-16.9%, 35.7%)  p=0.6296  ES=0.23 | 18.8% ( -6.9%, 51.6%)  p=0.1631  ES=0.67 | 11.6% (-12.9%, 43.0%)  p=0.3806  ES=0.43 |
| EEG Beta Pz-O1 closed (uV^2/Hz) | 0.6816 | -26.6% (-51.1%, 10.1%)  p=0.1329  ES=0.76 | -20.3% (-47.0%, 19.9%)  p=0.2727  ES=0.55 | -24.5% (-49.8%, 13.6%)  p=0.1747  ES=0.69 | 41.2% ( -4.3%, 108.4%)  p=0.0818  ES=0.84 | -5.8% (-35.9%, 38.5%)  p=0.7597  ES=0.14 | -7.7% (-37.7%, 36.6%)  p=0.6847  ES=0.20 |
| EEG Beta Pz-O1 open (uV^2/Hz) | 0.5726 | -22.0% (-46.8%, 14.3%)  p=0.1993  ES=0.64 | -25.3% (-49.2%, 9.8%)  p=0.1358  ES=0.75 | -21.1% (-46.4%, 16.2%)  p=0.2279  ES=0.61 | 25.6% (-13.3%, 81.8%)  p=0.2257  ES=0.59 | -0.8% (-31.2%, 43.2%)  p=0.9666  ES=0.02 | -12.6% (-39.9%, 27.2%)  p=0.4781  ES=0.35 |
| EEG Beta Pz-O2 closed (uV^2/Hz) | 0.9537 | -18.5% (-45.8%, 22.4%)  p=0.3198  ES=0.50 | -8.4% (-39.2%, 38.0%)  p=0.6716  ES=0.21 | -14.6% (-43.3%, 28.7%)  p=0.4471  ES=0.38 | 28.1% (-13.2%, 88.9%)  p=0.2090  ES=0.60 | 7.3% (-27.0%, 57.6%)  p=0.7182  ES=0.17 | -0.7% (-32.9%, 47.0%)  p=0.9730  ES=0.02 |
| EEG Beta Pz-O2 open (uV^2/Hz) | 0.9100 | -9.3% (-40.8%, 38.9%)  p=0.6490  ES=0.23 | -8.3% (-40.2%, 40.7%)  p=0.6890  ES=0.20 | 5.2% (-31.5%, 61.7%)  p=0.8133  ES=0.12 | 21.7% (-18.6%, 82.0%)  p=0.3340  ES=0.46 | 6.0% (-28.9%, 58.1%)  p=0.7726  ES=0.14 | 2.1% (-32.0%, 53.1%)  p=0.9207  ES=0.05 |
| EEG Delta Fz-Cz closed (uV^2/Hz) | 0.9730 | -11.1% (-37.2%, 25.8%)  p=0.5026  ES=0.34 | -2.6% (-31.3%, 38.2%)  p=0.8832  ES=0.07 | -14.1% (-39.5%, 21.9%)  p=0.3908  ES=0.44 | 7.8% (-22.3%, 49.6%)  p=0.6495  ES=0.22 | 29.5% ( -6.2%, 78.7%)  p=0.1147  ES=0.74 | 4.2% (-25.2%, 45.1%)  p=0.8072  ES=0.12 |
| EEG Delta Fz-Cz open (uV^2/Hz) | 0.8000 | -8.0% (-32.7%, 25.9%)  p=0.5997  ES=0.27 | 2.5% (-25.2%, 40.6%)  p=0.8756  ES=0.08 | -13.5% (-37.0%, 18.9%)  p=0.3682  ES=0.46 | 21.5% ( -9.4%, 63.0%)  p=0.1921  ES=0.62 | 29.8% ( -2.9%, 73.6%)  p=0.0780  ES=0.84 | 19.9% (-11.0%, 61.5%)  p=0.2305  ES=0.58 |
| EEG Delta Pz-O1 closed (uV^2/Hz) | 0.2120 | -44.1% (-62.0%, -17.8%)  p=0.0035  ES=1.49 | -20.4% (-46.0%, 17.5%)  p=0.2491  ES=0.58 | -18.6% (-44.8%, 20.2%)  p=0.2987  ES=0.52 | 42.5% ( -1.7%, 106.7%)  p=0.0613  ES=0.91 | 7.3% (-25.6%, 54.7%)  p=0.7039  ES=0.18 | -10.6% (-38.6%, 30.1%)  p=0.5544  ES=0.29 |
| EEG Delta Pz-O1 open (uV^2/Hz) | 0.2881 | -28.0% (-48.5%, 0.8%)  p=0.0557  ES=0.96 | -18.4% (-41.9%, 14.6%)  p=0.2384  ES=0.60 | -16.3% (-40.6%, 17.8%)  p=0.3053  ES=0.52 | 8.8% (-21.6%, 50.9%)  p=0.6112  ES=0.25 | 6.0% (-23.2%, 46.4%)  p=0.7199  ES=0.17 | -14.6% (-38.9%, 19.3%)  p=0.3520  ES=0.46 |
| EEG Delta Pz-O2 closed (uV^2/Hz) | 0.3351 | -41.5% (-60.0%, -14.2%)  p=0.0064  ES=1.39 | -11.3% (-39.6%, 30.4%)  p=0.5397  ES=0.31 | -12.7% (-40.6%, 28.2%)  p=0.4841  ES=0.35 | 16.3% (-19.2%, 67.4%)  p=0.4136  ES=0.39 | 5.0% (-26.7%, 50.2%)  p=0.7889  ES=0.13 | -9.6% (-37.5%, 30.7%)  p=0.5898  ES=0.26 |
| EEG Delta Pz-O2 open (uV^2/Hz) | 0.6586 | -27.0% (-48.3%, 3.0%)  p=0.0729  ES=0.91 | -17.0% (-41.4%, 17.6%)  p=0.2919  ES=0.54 | -8.6% (-35.6%, 29.7%)  p=0.6111  ES=0.26 | 12.8% (-18.7%, 56.5%)  p=0.4685  ES=0.35 | 1.6% (-26.4%, 40.4%)  p=0.9212  ES=0.05 | -2.5% (-30.2%, 36.2%)  p=0.8818  ES=0.07 |
| EEG Gamma Fz-Cz closed (uV^2/Hz) | 0.7953 | -5.1% (-23.0%, 16.9%)  p=0.6176  ES=0.25 | -3.0% (-21.3%, 19.6%)  p=0.7718  ES=0.14 | -0.9% (-19.6%, 22.2%)  p=0.9309  ES=0.04 | -1.8% (-19.9%, 20.3%)  p=0.8573  ES=0.09 | 0.8% (-17.6%, 23.4%)  p=0.9354  ES=0.04 | -0.2% (-18.7%, 22.5%)  p=0.9842  ES=0.01 |
| EEG Gamma Fz-Cz open (uV^2/Hz) | 0.2899 | -5.4% (-25.3%, 20.0%)  p=0.6456  ES=0.23 | 8.4% (-14.6%, 37.7%)  p=0.5015  ES=0.34 | 3.6% (-18.5%, 31.6%)  p=0.7732  ES=0.15 | 4.6% (-17.1%, 32.1%)  p=0.7006  ES=0.19 | 5.8% (-16.0%, 33.3%)  p=0.6298  ES=0.23 | -0.1% (-21.1%, 26.5%)  p=0.9919  ES=0.01 |
| EEG Gamma Pz-O1 closed (uV^2/Hz) | 0.9511 | 4.9% (-37.3%, 75.4%)  p=0.8537  ES=0.09 | -5.4% (-43.6%, 58.6%)  p=0.8308  ES=0.11 | -15.5% (-49.6%, 41.7%)  p=0.5198  ES=0.32 | 5.4% (-35.7%, 72.6%)  p=0.8332  ES=0.10 | -6.2% (-42.4%, 52.8%)  p=0.7955  ES=0.12 | -26.4% (-55.3%, 21.2%)  p=0.2256  ES=0.59 |
| EEG Gamma Pz-O1 open (uV^2/Hz) | 0.8633 | -11.5% (-44.3%, 40.4%)  p=0.6001  ES=0.26 | -18.7% (-48.9%, 29.5%)  p=0.3804  ES=0.44 | -19.6% (-49.6%, 28.4%)  p=0.3579  ES=0.46 | 3.8% (-33.5%, 62.0%)  p=0.8684  ES=0.08 | 10.1% (-29.2%, 71.1%)  p=0.6660  ES=0.20 | -31.2% (-56.2%, 8.2%)  p=0.1046  ES=0.79 |
| EEG Gamma Pz-O2 closed (uV^2/Hz) | 0.7989 | 2.6% (-36.3%, 65.3%)  p=0.9137  ES=0.05 | 5.1% (-34.9%, 69.6%)  p=0.8363  ES=0.10 | -16.0% (-47.9%, 35.6%)  p=0.4715  ES=0.36 | 0.8% (-36.2%, 59.2%)  p=0.9724  ES=0.02 | 0.7% (-36.0%, 58.3%)  p=0.9766  ES=0.01 | -12.3% (-44.7%, 39.0%)  p=0.5718  ES=0.27 |
| EEG Gamma Pz-O2 open (uV^2/Hz) | 0.3343 | -2.7% (-41.0%, 60.5%)  p=0.9144  ES=0.05 | -8.3% (-44.5%, 51.5%)  p=0.7321  ES=0.17 | 12.8% (-31.8%, 86.7%)  p=0.6357  ES=0.24 | 12.0% (-30.2%, 79.9%)  p=0.6351  ES=0.22 | 19.7% (-25.2%, 91.6%)  p=0.4494  ES=0.36 | -7.3% (-42.5%, 49.5%)  p=0.7537  ES=0.15 |
| EEG Theta Fz-Cz closed (uV^2/Hz) | 0.7182 | -4.6% (-26.5%, 23.7%)  p=0.7186  ES=0.18 | 8.8% (-16.3%, 41.5%)  p=0.5241  ES=0.32 | 8.6% (-16.5%, 41.1%)  p=0.5366  ES=0.31 | 4.4% (-18.3%, 33.4%)  p=0.7268  ES=0.17 | 15.2% ( -9.6%, 46.6%)  p=0.2494  ES=0.54 | 7.3% (-16.2%, 37.5%)  p=0.5729  ES=0.27 |
| EEG Theta Fz-Cz open (uV^2/Hz) | 0.1814 | -8.2% (-29.6%, 19.6%)  p=0.5218  ES=0.33 | 26.4% ( -3.1%, 65.0%)  p=0.0838  ES=0.89 | 15.5% (-11.6%, 51.0%)  p=0.2872  ES=0.55 | 9.9% (-14.1%, 40.6%)  p=0.4496  ES=0.36 | 40.3% ( 9.9%, 79.0%)  p=0.0071  ES=1.29 | 24.1% ( -3.3%, 59.2%)  p=0.0893  ES=0.82 |
| EEG Theta Pz-O1 closed (uV^2/Hz) | 0.5322 | -33.6% (-59.5%, 9.0%)  p=0.1040  ES=0.82 | -6.0% (-42.8%, 54.6%)  p=0.8052  ES=0.12 | -1.2% (-39.9%, 62.5%)  p=0.9616  ES=0.02 | 24.4% (-22.5%, 99.6%)  p=0.3612  ES=0.44 | -26.2% (-53.8%, 17.8%)  p=0.2000  ES=0.61 | -27.5% (-55.0%, 16.8%)  p=0.1836  ES=0.64 |
| EEG Theta Pz-O1 open (uV^2/Hz) | 0.8667 | -23.7% (-48.7%, 13.5%)  p=0.1793  ES=0.67 | -1.7% (-34.1%, 46.5%)  p=0.9307  ES=0.04 | 1.9% (-31.8%, 52.3%)  p=0.9245  ES=0.05 | 1.5% (-30.7%, 48.7%)  p=0.9373  ES=0.04 | -13.6% (-40.8%, 26.1%)  p=0.4459  ES=0.36 | -14.6% (-42.0%, 25.8%)  p=0.4215  ES=0.39 |
| EEG Theta Pz-O2 closed (uV^2/Hz) | 0.4044 | -29.6% (-55.7%, 11.9%)  p=0.1363  ES=0.75 | 9.6% (-31.2%, 74.6%)  p=0.6961  ES=0.20 | 4.5% (-34.4%, 66.5%)  p=0.8525  ES=0.09 | 8.7% (-29.9%, 68.4%)  p=0.7070  ES=0.18 | -15.2% (-45.0%, 30.8%)  p=0.4524  ES=0.35 | -21.6% (-49.6%, 22.0%)  p=0.2767  ES=0.52 |
| EEG Theta Pz-O2 open (uV^2/Hz) | 0.9005 | -16.1% (-45.1%, 28.1%)  p=0.4113  ES=0.41 | 10.6% (-27.8%, 69.3%)  p=0.6411  ES=0.23 | 15.8% (-24.5%, 77.7%)  p=0.4969  ES=0.34 | 4.1% (-30.2%, 55.3%)  p=0.8422  ES=0.09 | -6.7% (-37.3%, 38.6%)  p=0.7271  ES=0.16 | -4.7% (-36.5%, 43.1%)  p=0.8163  ES=0.11 |

|  | | **Contrasts** | | | | | |
| --- | --- | --- | --- | --- | --- | --- | --- |
|  | | **Adults** | | | **Elderly** | | |
| **Parameter** | **Treatment P-value** | **Day 1 HTL0018318 (20mg) Placebo** | **Day 5 HTL0018318 (20mg) Placebo** | **Day 10 HTL0018318 (20mg) Placebo** | **Day 1 HTL0018318 (20mg) Placebo** | **Day 5 HTL0018318 (20mg) Placebo** | **Day 10 HTL0018318 (20mg) Placebo** |
| Saliva (g) | 0.2181 | -0.150 ( -1.270, 0.971)  p=0.7912  ES=0.13 | 0.905 ( -0.376, 2.186)  p=0.1647  ES=0.79 | 1.347 ( 0.226, 2.468)  p=0.0192  ES=1.17 | 0.206 ( -0.848, 1.259)  p=0.6988  ES=0.18 | 0.132 ( -1.084, 1.347)  p=0.8309  ES=0.11 | 0.407 ( -0.651, 1.466)  p=0.4455  ES=0.35 |
| LSEQ: Getting to sleep (mm) | 0.4138 | 0.01 ( -2.66, 2.68)  p=0.9914  ES=0.01 | 0.38 ( -2.29, 3.06)  p=0.7769  ES=0.19 | 0.72 ( -1.95, 3.39)  p=0.5971  ES=0.36 | 3.99 ( 1.34, 6.64)  p=0.0033  ES=2.01 | 1.16 ( -1.49, 3.81)  p=0.3899  ES=0.58 | -0.08 ( -2.79, 2.62)  p=0.9509  ES=0.04 |
| LSEQ: Quality of sleep (mm) | 0.6614 | 0.84 ( -5.29, 6.98)  p=0.7870  ES=0.18 | 3.18 ( -2.96, 9.31)  p=0.3091  ES=0.66 | 2.73 ( -3.41, 8.87)  p=0.3816  ES=0.57 | -0.40 ( -6.58, 5.77)  p=0.8985  ES=0.08 | 1.39 ( -4.78, 7.57)  p=0.6569  ES=0.29 | 3.82 ( -2.46, 10.10)  p=0.2321  ES=0.80 |
| LSEQ: Awake following sleep (mm) | 0.6424 | 0.89 ( -4.70, 6.49)  p=0.7530  ES=0.18 | 1.34 ( -4.26, 6.93)  p=0.6375  ES=0.27 | 3.45 ( -2.15, 9.04)  p=0.2254  ES=0.70 | 0.71 ( -5.11, 6.52)  p=0.8106  ES=0.14 | -1.04 ( -6.85, 4.77)  p=0.7244  ES=0.21 | 0.20 ( -5.67, 6.07)  p=0.9464  ES=0.04 |
| LSEQ: Behaviour after wake (mm) | 0.2364 | 0.67 ( -5.31, 6.65)  p=0.8256  ES=0.13 | -0.48 ( -6.46, 5.50)  p=0.8745  ES=0.10 | 2.82 ( -3.16, 8.79)  p=0.3538  ES=0.56 | -0.26 ( -6.07, 5.56)  p=0.9305  ES=0.05 | 1.03 ( -4.78, 6.85)  p=0.7269  ES=0.20 | -2.39 ( -8.29, 3.50)  p=0.4245  ES=0.48 |
| Systolic BP supine (mmHg) | 0.3170 | 5.1 ( -2.0, 12.2)  p=0.1551  ES=0.65 | 2.1 ( -5.0, 9.2)  p=0.5590  ES=0.26 | 2.1 ( -5.0, 9.2)  p=0.5520  ES=0.27 | 2.9 ( -4.1, 9.8)  p=0.4135  ES=0.36 | -9.7 ( -16.6, -2.7)  p=0.0068  ES=1.22 | -3.9 ( -10.8, 3.1)  p=0.2738  ES=0.49 |
| Diastolic BP supine (mmHg) | 0.0135 | 2.6 ( -2.2, 7.4)  p=0.2821  ES=0.49 | -5.7 ( -10.5, -0.9)  p=0.0210  ES=1.06 | -4.8 ( -9.6, 0.0)  p=0.0506  ES=0.89 | 0.8 ( -3.9, 5.6)  p=0.7316  ES=0.15 | -9.3 ( -14.0, -4.6)  p=0.0002  ES=1.74 | -6.6 ( -11.4, -1.9)  p=0.0069  ES=1.24 |
| Pulse Rate supine (bpm) | 0.0112 | 0.9 ( -3.8, 5.5)  p=0.7108  ES=0.17 | 3.5 ( -1.1, 8.1)  p=0.1320  ES=0.68 | 2.5 ( -2.2, 7.1)  p=0.2932  ES=0.47 | 7.9 ( 3.4, 12.4)  p=0.0007  ES=1.53 | 9.6 ( 5.1, 14.2)  p=<.0001  ES=1.86 | 8.3 ( 3.7, 12.8)  p=0.0005  ES=1.59 |
| Systolic BP standing (mmHg) | 0.6207 | 8.0 ( 0.3, 15.7)  p=0.0427  ES=0.92 | 1.2 ( -6.5, 8.9)  p=0.7630  ES=0.14 | 3.4 ( -4.3, 11.1)  p=0.3811  ES=0.39 | -2.1 ( -9.7, 5.5)  p=0.5818  ES=0.24 | -12.4 ( -19.9, -4.8)  p=0.0016  ES=1.43 | -6.1 ( -13.7, 1.5)  p=0.1163  ES=0.70 |
| Diastolic BP standing (mmHg) | 0.1009 | 5.5 ( -0.1, 11.1)  p=0.0549  ES=0.88 | -2.8 ( -8.4, 2.8)  p=0.3241  ES=0.45 | -1.9 ( -7.5, 3.7)  p=0.4969  ES=0.31 | 0.2 ( -5.3, 5.6)  p=0.9560  ES=0.02 | -11.3 ( -16.8, -5.8)  p=<.0001  ES=1.82 | -9.2 ( -14.7, -3.7)  p=0.0013  ES=1.48 |
| Pulse Rate standing (bpm) | 0.2866 | -0.2 ( -5.8, 5.4)  p=0.9358  ES=0.04 | 3.3 ( -2.3, 8.9)  p=0.2393  ES=0.53 | -0.7 ( -6.3, 4.9)  p=0.8066  ES=0.11 | 9.2 ( 3.7, 14.6)  p=0.0011  ES=1.47 | 9.7 ( 4.3, 15.1)  p=0.0006  ES=1.56 | 8.3 ( 2.9, 13.8)  p=0.0032  ES=1.33 |
| Systolic BP sup-sta (mmHg) | 0.7415 | -3.0 ( -8.5, 2.4)  p=0.2736  ES=0.48 | 0.8 ( -4.7, 6.2)  p=0.7846  ES=0.12 | -1.5 ( -6.9, 4.0)  p=0.5989  ES=0.23 | 4.5 ( -0.8, 9.9)  p=0.0939  ES=0.72 | 2.4 ( -2.9, 7.8)  p=0.3648  ES=0.38 | 1.9 ( -3.4, 7.3)  p=0.4794  ES=0.30 |
| Diastolic BP sup-sta (mmHg) | 0.9738 | -1.3 ( -4.8, 2.2)  p=0.4661  ES=0.32 | -1.3 ( -4.8, 2.2)  p=0.4597  ES=0.32 | -1.3 ( -4.8, 2.2)  p=0.4661  ES=0.32 | 0.9 ( -2.5, 4.3)  p=0.6006  ES=0.22 | 2.3 ( -1.0, 5.7)  p=0.1701  ES=0.58 | 2.9 ( -0.5, 6.3)  p=0.0935  ES=0.71 |
| Pulse Rate sup-sta (bpm) | 0.5454 | 1.9 ( -2.0, 5.8)  p=0.3370  ES=0.42 | 0.9 ( -3.0, 4.8)  p=0.6555  ES=0.20 | 4.0 ( 0.1, 7.9)  p=0.0471  ES=0.88 | -1.1 ( -4.9, 2.7)  p=0.5575  ES=0.25 | 0.2 ( -3.6, 3.9)  p=0.9274  ES=0.04 | 0.2 ( -3.6, 4.0)  p=0.9147  ES=0.05 |
| Track Performance (%) | 0.3407 | 0.260 ( -2.661, 3.181)  p=0.8598  ES=0.09 | 0.838 ( -2.082, 3.759)  p=0.5694  ES=0.28 | 0.325 ( -2.596, 3.246)  p=0.8254  ES=0.11 | 3.605 ( 0.672, 6.539)  p=0.0167  ES=1.19 | 2.351 ( -0.582, 5.285)  p=0.1146  ES=0.78 | 2.680 ( -0.267, 5.626)  p=0.0740  ES=0.88 |
| N-back corr-incorr/total 0 | 0.1986 | 0.020 ( -0.014, 0.053)  p=0.2491  ES=0.57 | 0.022 ( -0.011, 0.056)  p=0.1940  ES=0.64 | 0.000 ( -0.034, 0.034)  p=0.9956  ES=0.00 | -0.005 ( -0.037, 0.026)  p=0.7420  ES=0.15 | 0.011 ( -0.021, 0.042)  p=0.5018  ES=0.31 | 0.005 ( -0.027, 0.037)  p=0.7383  ES=0.16 |
| N-back corr-incorr/total 1 | 0.0605 | 0.067 ( 0.015, 0.120)  p=0.0127  ES=1.21 | 0.054 ( 0.001, 0.107)  p=0.0449  ES=0.97 | 0.009 ( -0.045, 0.062)  p=0.7440  ES=0.16 | 0.021 ( -0.031, 0.072)  p=0.4284  ES=0.37 | 0.028 ( -0.023, 0.080)  p=0.2831  ES=0.50 | 0.006 ( -0.046, 0.058)  p=0.8259  ES=0.10 |
| N-back corr-incorr/total 2 | 0.0024 | 0.070 ( 0.001, 0.140)  p=0.0476  ES=0.95 | 0.059 ( -0.010, 0.129)  p=0.0947  ES=0.80 | 0.081 ( 0.011, 0.151)  p=0.0237  ES=1.09 | 0.145 ( 0.077, 0.213)  p=<.0001  ES=1.96 | 0.076 ( 0.008, 0.145)  p=0.0299  ES=1.03 | 0.051 ( -0.019, 0.120)  p=0.1503  ES=0.68 |
| N-back mean RT 0 back (msec) | 0.7917 | 31.1 ( -12.2, 74.4)  p=0.1569  ES=0.67 | 11.6 ( -31.7, 54.9)  p=0.5951  ES=0.25 | 10.9 ( -32.7, 54.4)  p=0.6214  ES=0.24 | -14.8 ( -57.2, 27.5)  p=0.4876  ES=0.32 | -32.4 ( -74.7, 9.9)  p=0.1312  ES=0.70 | -31.0 ( -73.6, 11.5)  p=0.1510  ES=0.67 |
| N-back mean RT 1 back (msec) | 0.9521 | 31.1 ( -21.5, 83.7)  p=0.2426  ES=0.56 | 31.1 ( -21.5, 83.7)  p=0.2432  ES=0.56 | 18.2 ( -34.7, 71.1)  p=0.4958  ES=0.33 | -12.8 ( -64.9, 39.3)  p=0.6264  ES=0.23 | -14.5 ( -66.6, 37.6)  p=0.5820  ES=0.26 | -30.5 ( -82.7, 21.8)  p=0.2502  ES=0.54 |
| N-back mean RT 2 back (msec) | 0.3380 | 17.1 ( -57.1, 91.4)  p=0.6479  ES=0.22 | 19.5 ( -54.7, 93.7)  p=0.6029  ES=0.25 | 18.2 ( -56.3, 92.7)  p=0.6286  ES=0.23 | -85.0 ( -157.5, -12.6)  p=0.0220  ES=1.08 | -74.1 ( -146.9, -1.3)  p=0.0462  ES=0.94 | -70.6 ( -143.6, 2.5)  p=0.0581  ES=0.89 |
| MMTImm: Expl Error | 0.0082 | -0.9 ( -5.9, 4.1)  p=0.7204  ES=0.17 | -0.4 ( -5.4, 4.6)  p=0.8637  ES=0.08 | 3.3 ( -1.7, 8.3)  p=0.1930  ES=0.63 | -3.1 ( -7.9, 1.7)  p=0.2061  ES=0.59 | -6.7 ( -11.5, -1.9)  p=0.0066  ES=1.28 | -4.3 ( -9.2, 0.5)  p=0.0802  ES=0.83 |
| MMTImm: Total Moves | 0.0192 | -0.7 ( -10.9, 9.6)  p=0.8991  ES=0.06 | 0.6 ( -9.7, 10.8)  p=0.9129  ES=0.05 | 7.7 ( -2.6, 18.0)  p=0.1394  ES=0.72 | -7.0 ( -16.8, 2.8)  p=0.1590  ES=0.66 | -14.3 ( -24.1, -4.5)  p=0.0045  ES=1.34 | -8.9 ( -18.8, 1.0)  p=0.0790  ES=0.83 |
| MMTImm: Expl Time (msec) | 0.9288 | 689.5 ( -16342, 17721.3)  p=0.9360  ES=0.04 | 10043.3 (-6988.5, 27075.1)  p=0.2443  ES=0.56 | 12043.5 (-5014.0, 29101.1)  p=0.1640  ES=0.67 | -16751 ( -33410, -92.0)  p=0.0488  ES=0.94 | -4707.3 ( -21366, 11951.8)  p=0.5757  ES=0.26 | 4300.7 ( -12462, 21063.0)  p=0.6114  ES=0.24 |
| MMTRev: Expl Error | 0.0366 | -1.1 ( -2.9, 0.7)  p=0.2347  ES=0.57 | -0.0 ( -1.8, 1.7)  p=0.9592  ES=0.02 | 0.3 ( -1.5, 2.1)  p=0.7500  ES=0.15 | -1.8 ( -3.5, -0.0)  p=0.0487  ES=0.92 | -2.0 ( -3.7, -0.2)  p=0.0284  ES=1.03 | -1.3 ( -3.0, 0.5)  p=0.1593  ES=0.66 |
| MMTRev: Total Moves | 0.0251 | -2.1 ( -5.7, 1.5)  p=0.2515  ES=0.55 | -0.1 ( -3.7, 3.5)  p=0.9524  ES=0.03 | 0.6 ( -3.1, 4.2)  p=0.7604  ES=0.15 | -3.4 ( -7.0, 0.1)  p=0.0586  ES=0.89 | -4.5 ( -8.0, -0.9)  p=0.0136  ES=1.16 | -2.7 ( -6.3, 0.9)  p=0.1376  ES=0.70 |
| MMTRev: Expl Time (msec) | 0.2267 | 959.8 (-3047.9, 4967.5)  p=0.6364  ES=0.22 | 821.9 (-3185.8, 4829.6)  p=0.6855  ES=0.19 | 2240.6 (-1780.7, 6261.8)  p=0.2723  ES=0.52 | -3771.7 (-7788.5, 245.2)  p=0.0655  ES=0.88 | -731.0 (-4747.9, 3285.9)  p=0.7193  ES=0.17 | 2271.1 (-1793.9, 6336.0)  p=0.2710  ES=0.53 |
| MMTDel: Expl Error | 0.1938 | -0.3 ( -1.7, 1.2)  p=0.6900  ES=0.19 | -1.4 ( -2.8, 0.1)  p=0.0628  ES=0.89 | -0.5 ( -1.9, 1.0)  p=0.5352  ES=0.30 | -1.1 ( -2.5, 0.4)  p=0.1418  ES=0.68 | -1.1 ( -2.6, 0.3)  p=0.1132  ES=0.74 | -0.6 ( -2.1, 0.8)  p=0.3742  ES=0.42 |
| MMTDel: Total Moves | 0.1404 | -0.6 ( -3.5, 2.3)  p=0.6892  ES=0.19 | -2.7 ( -5.7, 0.2)  p=0.0675  ES=0.88 | -0.9 ( -3.8, 2.0)  p=0.5488  ES=0.29 | -2.8 ( -5.7, 0.0)  p=0.0534  ES=0.90 | -2.5 ( -5.3, 0.4)  p=0.0892  ES=0.79 | -1.6 ( -4.5, 1.3)  p=0.2729  ES=0.52 |
| MMTDel: Expl Time (msec) | 0.6223 | -246.5 (-4459.7, 3966.7)  p=0.9078  ES=0.06 | -1215.5 (-5428.7, 2997.7)  p=0.5684  ES=0.27 | 1057.1 (-3166.3, 5280.5)  p=0.6206  ES=0.24 | -4716.4 (-8876.5, -556.3)  p=0.0267  ES=1.05 | -1526.6 (-5686.8, 2633.5)  p=0.4682  ES=0.34 | 124.5 (-4075.3, 4324.4)  p=0.9532  ES=0.03 |
| Left Pupil/Iris ratio | 0.9442 | 0.02728 (-.00484, 0.05941)  p=0.0950  ES=0.80 | -.01062 (-.04270, 0.02145)  p=0.5119  ES=0.31 | -.01038 (-.04246, 0.02170)  p=0.5216  ES=0.31 | 0.01087 (-.02021, 0.04195)  p=0.4886  ES=0.32 | 0.01557 (-.01547, 0.04660)  p=0.3215  ES=0.46 | 0.00703 (-.02447, 0.03852)  p=0.6586  ES=0.21 |
| Right Pupil/Iris ratio | 0.8405 | 0.01195 (-.02198, 0.04587)  p=0.4857  ES=0.33 | -.01926 (-.05314, 0.01462)  p=0.2615  ES=0.54 | -.01117 (-.04505, 0.02271)  p=0.5137  ES=0.31 | 0.01715 (-.01555, 0.04985)  p=0.2999  ES=0.48 | 0.02013 (-.01253, 0.05279)  p=0.2237  ES=0.56 | 0.01325 (-.01986, 0.04636)  p=0.4286  ES=0.37 |
| VAS Alertness (mm) | 0.9337 | -2.06 ( -5.42, 1.30)  p=0.2261  ES=0.58 | 0.35 ( -3.02, 3.71)  p=0.8384  ES=0.10 | -1.50 ( -4.87, 1.87)  p=0.3789  ES=0.42 | 0.67 ( -2.60, 3.94)  p=0.6848  ES=0.19 | -0.36 ( -3.63, 2.91)  p=0.8269  ES=0.10 | -1.40 ( -4.68, 1.89)  p=0.4005  ES=0.39 |
| VAS Calmness (mm) | 0.6199 | -1.25 ( -5.25, 2.74)  p=0.5348  ES=0.29 | -1.59 ( -5.58, 2.41)  p=0.4323  ES=0.37 | -1.79 ( -5.79, 2.21)  p=0.3775  ES=0.42 | -0.74 ( -4.67, 3.18)  p=0.7074  ES=0.17 | -0.72 ( -4.64, 3.20)  p=0.7163  ES=0.17 | -2.41 ( -6.37, 1.54)  p=0.2287  ES=0.57 |
| VAS Mood (mm) | 0.6981 | -2.76 ( -6.48, 0.95)  p=0.1429  ES=0.70 | -1.21 ( -4.92, 2.51)  p=0.5198  ES=0.31 | -2.59 ( -6.31, 1.14)  p=0.1707  ES=0.66 | 0.92 ( -2.69, 4.53)  p=0.6130  ES=0.23 | 0.34 ( -3.27, 3.95)  p=0.8509  ES=0.09 | -1.34 ( -4.97, 2.29)  p=0.4659  ES=0.34 |
| VAS Nausea (log(mm)) | 0.3618 | 0.02 ( -0.10, 0.15)  p=0.7189  ES=0.17 | 0.04 ( -0.09, 0.17)  p=0.5249  ES=0.30 | -0.00 ( -0.13, 0.13)  p=0.9960  ES=0.00 | -0.01 ( -0.13, 0.11)  p=0.8694  ES=0.08 | -0.15 ( -0.28, -0.03)  p=0.0152  ES=1.14 | -0.10 ( -0.23, 0.02)  p=0.1027  ES=0.77 |
| EEG Alpha Fz-Cz closed (uV^2/Hz) | 0.8835 | 15.9% (-20.0%, 67.9%)  p=0.4320  ES=0.39 | 17.6% (-18.8%, 70.2%)  p=0.3867  ES=0.43 | 55.1% ( 7.3%, 124.1%)  p=0.0202  ES=1.15 | -2.4% (-31.5%, 39.2%)  p=0.8935  ES=0.06 | -14.6% (-39.7%, 21.1%)  p=0.3723  ES=0.41 | -28.2% (-49.5%, 2.1%)  p=0.0646  ES=0.87 |
| EEG Alpha Fz-Cz open (uV^2/Hz) | 0.8005 | 15.5% (-17.5%, 61.6%)  p=0.3977  ES=0.42 | 22.0% (-12.9%, 70.7%)  p=0.2442  ES=0.58 | 61.4% ( 15.7%, 125.1%)  p=0.0054  ES=1.40 | -4.2% (-30.9%, 32.7%)  p=0.7937  ES=0.13 | -8.6% (-34.0%, 26.7%)  p=0.5862  ES=0.26 | -21.3% (-43.4%, 9.2%)  p=0.1497  ES=0.70 |
| EEG Alpha Pz-O1 closed (uV^2/Hz) | 0.6978 | -2.1% (-48.6%, 86.3%)  p=0.9469  ES=0.03 | 20.4% (-36.7%, 128.8%)  p=0.5666  ES=0.28 | -2.9% (-48.8%, 84.0%)  p=0.9266  ES=0.05 | -0.9% (-46.4%, 83.3%)  p=0.9770  ES=0.01 | -19.0% (-55.8%, 48.5%)  p=0.4909  ES=0.32 | -45.2% (-70.2%, 1.0%)  p=0.0536  ES=0.92 |
| EEG Alpha Pz-O1 open (uV^2/Hz) | 0.7428 | 12.4% (-38.7%, 105.9%)  p=0.7030  ES=0.19 | 28.4% (-29.9%, 135.2%)  p=0.4142  ES=0.41 | 6.6% (-41.5%, 94.4%)  p=0.8323  ES=0.10 | -18.9% (-55.0%, 46.2%)  p=0.4815  ES=0.34 | -22.0% (-56.7%, 40.6%)  p=0.4046  ES=0.40 | -40.3% (-67.0%, 7.8%)  p=0.0864  ES=0.84 |
| EEG Alpha Pz-O2 closed (uV^2/Hz) | 0.8168 | 13.9% (-38.9%, 112.3%)  p=0.6798  ES=0.20 | 24.1% (-33.4%, 131.1%)  p=0.4918  ES=0.34 | -34.5% (-64.7%, 21.6%)  p=0.1773  ES=0.66 | -9.0% (-49.9%, 65.2%)  p=0.7547  ES=0.15 | 4.6% (-41.8%, 88.2%)  p=0.8785  ES=0.07 | -6.7% (-48.4%, 68.5%)  p=0.8151  ES=0.11 |
| EEG Alpha Pz-O2 open (uV^2/Hz) | 0.9830 | 20.0% (-34.4%, 119.4%)  p=0.5492  ES=0.30 | 33.9% (-26.8%, 144.7%)  p=0.3386  ES=0.48 | -10.2% (-50.7%, 63.4%)  p=0.7212  ES=0.18 | -15.2% (-52.7%, 52.0%)  p=0.5761  ES=0.27 | -1.5% (-45.0%, 76.4%)  p=0.9578  ES=0.03 | 0.8% (-43.9%, 81.1%)  p=0.9796  ES=0.01 |
| EEG Beta Fz-Cz closed (uV^2/Hz) | 0.8465 | 10.8% (-13.6%, 42.2%)  p=0.4139  ES=0.41 | 29.2% ( 0.8%, 65.7%)  p=0.0433  ES=1.01 | 23.5% ( -3.5%, 58.1%)  p=0.0929  ES=0.83 | -0.4% (-21.4%, 26.2%)  p=0.9739  ES=0.02 | -11.5% (-29.9%, 11.6%)  p=0.2979  ES=0.48 | -22.8% (-38.9%, -2.3%)  p=0.0314  ES=1.02 |
| EEG Beta Fz-Cz open (uV^2/Hz) | 0.7636 | -2.5% (-24.3%, 25.8%)  p=0.8462  ES=0.10 | 16.6% ( -9.6%, 50.3%)  p=0.2338  ES=0.60 | 27.1% ( -1.2%, 63.4%)  p=0.0616  ES=0.94 | 11.0% (-13.1%, 41.8%)  p=0.3998  ES=0.41 | -5.7% (-26.2%, 20.5%)  p=0.6351  ES=0.23 | -11.0% (-30.4%, 13.9%)  p=0.3517  ES=0.45 |
| EEG Beta Pz-O1 closed (uV^2/Hz) | 0.6816 | -8.9% (-39.3%, 36.7%)  p=0.6496  ES=0.23 | -2.7% (-35.0%, 45.9%)  p=0.8950  ES=0.07 | -25.6% (-50.2%, 11.3%)  p=0.1481  ES=0.72 | -5.7% (-35.9%, 38.7%)  p=0.7627  ES=0.14 | -4.9% (-34.8%, 38.8%)  p=0.7928  ES=0.12 | -20.2% (-45.5%, 16.9%)  p=0.2436  ES=0.55 |
| EEG Beta Pz-O1 open (uV^2/Hz) | 0.5726 | -13.3% (-41.2%, 27.8%)  p=0.4671  ES=0.37 | -14.4% (-41.9%, 26.3%)  p=0.4296  ES=0.40 | -30.5% (-52.6%, 1.9%)  p=0.0619  ES=0.94 | -2.0% (-32.5%, 42.4%)  p=0.9154  ES=0.05 | 2.3% (-29.6%, 48.5%)  p=0.9046  ES=0.06 | -12.4% (-39.9%, 27.7%)  p=0.4887  ES=0.34 |
| EEG Beta Pz-O2 closed (uV^2/Hz) | 0.9537 | 0.7% (-33.0%, 51.3%)  p=0.9722  ES=0.02 | 6.7% (-28.9%, 60.1%)  p=0.7525  ES=0.16 | -41.2% (-60.7%, -11.9%)  p=0.0105  ES=1.29 | -9.3% (-38.4%, 33.6%)  p=0.6183  ES=0.24 | 5.9% (-27.5%, 54.7%)  p=0.7655  ES=0.14 | 4.5% (-28.7%, 53.3%)  p=0.8191  ES=0.11 |
| EEG Beta Pz-O2 open (uV^2/Hz) | 0.9100 | -6.1% (-38.8%, 44.3%)  p=0.7727  ES=0.15 | -10.5% (-41.7%, 37.4%)  p=0.6075  ES=0.26 | -32.1% (-55.6%, 4.0%)  p=0.0743  ES=0.91 | -1.6% (-34.5%, 47.8%)  p=0.9383  ES=0.04 | 4.5% (-30.4%, 56.9%)  p=0.8316  ES=0.10 | 15.7% (-23.2%, 74.2%)  p=0.4819  ES=0.34 |
| EEG Delta Fz-Cz closed (uV^2/Hz) | 0.9730 | 7.1% (-24.2%, 51.3%)  p=0.6952  ES=0.20 | 7.0% (-24.1%, 51.0%)  p=0.6962  ES=0.20 | 2.0% (-27.4%, 43.4%)  p=0.9076  ES=0.06 | -6.9% (-33.3%, 30.1%)  p=0.6750  ES=0.20 | 3.1% (-25.4%, 42.5%)  p=0.8509  ES=0.09 | -20.9% (-43.1%, 9.9%)  p=0.1611  ES=0.68 |
| EEG Delta Fz-Cz open (uV^2/Hz) | 0.8000 | 0.1% (-27.0%, 37.1%)  p=0.9962  ES=0.00 | 5.5% (-23.0%, 44.6%)  p=0.7365  ES=0.17 | 6.7% (-21.8%, 45.4%)  p=0.6809  ES=0.21 | 13.8% (-15.8%, 53.7%)  p=0.3976  ES=0.41 | 19.7% (-11.4%, 61.6%)  p=0.2389  ES=0.58 | 10.3% (-18.6%, 49.5%)  p=0.5225  ES=0.32 |
| EEG Delta Pz-O1 closed (uV^2/Hz) | 0.2120 | -17.1% (-44.0%, 22.5%)  p=0.3430  ES=0.48 | -6.6% (-36.7%, 37.9%)  p=0.7307  ES=0.17 | 2.8% (-30.1%, 51.2%)  p=0.8874  ES=0.07 | 7.9% (-25.7%, 56.5%)  p=0.6881  ES=0.19 | -5.2% (-33.8%, 36.0%)  p=0.7713  ES=0.14 | -22.7% (-46.4%, 11.5%)  p=0.1670  ES=0.66 |
| EEG Delta Pz-O1 open (uV^2/Hz) | 0.2881 | -12.0% (-37.7%, 24.3%)  p=0.4650  ES=0.37 | -22.4% (-45.1%, 9.5%)  p=0.1476  ES=0.74 | -16.4% (-40.4%, 17.1%)  p=0.2954  ES=0.53 | 13.7% (-18.4%, 58.4%)  p=0.4466  ES=0.38 | -4.5% (-31.5%, 33.0%)  p=0.7837  ES=0.14 | -16.5% (-40.3%, 16.8%)  p=0.2906  ES=0.53 |
| EEG Delta Pz-O2 closed (uV^2/Hz) | 0.3351 | -7.1% (-37.0%, 36.9%)  p=0.7058  ES=0.19 | 10.7% (-24.7%, 62.8%)  p=0.6025  ES=0.26 | -3.4% (-34.1%, 41.6%)  p=0.8575  ES=0.09 | -6.1% (-34.9%, 35.5%)  p=0.7363  ES=0.16 | 6.4% (-25.4%, 51.7%)  p=0.7294  ES=0.16 | -11.3% (-38.2%, 27.2%)  p=0.5109  ES=0.31 |
| EEG Delta Pz-O2 open (uV^2/Hz) | 0.6586 | -7.7% (-35.0%, 31.3%)  p=0.6551  ES=0.23 | -11.7% (-37.9%, 25.4%)  p=0.4834  ES=0.36 | -13.3% (-38.6%, 22.5%)  p=0.4154  ES=0.41 | 14.6% (-17.9%, 60.0%)  p=0.4211  ES=0.39 | 3.7% (-25.7%, 44.8%)  p=0.8276  ES=0.11 | -2.8% (-30.6%, 36.2%)  p=0.8678  ES=0.08 |
| EEG Gamma Fz-Cz closed (uV^2/Hz) | 0.7953 | 4.4% (-15.1%, 28.5%)  p=0.6782  ES=0.20 | 22.7% ( -0.3%, 50.9%)  p=0.0530  ES=0.96 | 12.0% ( -8.9%, 37.6%)  p=0.2789  ES=0.53 | -3.6% (-21.2%, 17.9%)  p=0.7153  ES=0.17 | -2.9% (-20.4%, 18.3%)  p=0.7664  ES=0.14 | -9.6% (-26.0%, 10.4%)  p=0.3197  ES=0.47 |
| EEG Gamma Fz-Cz open (uV^2/Hz) | 0.2899 | -3.2% (-23.7%, 22.8%)  p=0.7851  ES=0.14 | 17.6% ( -7.3%, 49.2%)  p=0.1803  ES=0.67 | 17.6% ( -7.0%, 48.8%)  p=0.1742  ES=0.68 | 23.8% ( -1.8%, 56.0%)  p=0.0700  ES=0.89 | 6.1% (-15.8%, 33.6%)  p=0.6134  ES=0.25 | 7.9% (-14.5%, 36.2%)  p=0.5184  ES=0.32 |
| EEG Gamma Pz-O1 closed (uV^2/Hz) | 0.9511 | 6.5% (-36.3%, 77.8%)  p=0.8094  ES=0.12 | -3.2% (-41.9%, 61.5%)  p=0.9012  ES=0.06 | -21.5% (-52.7%, 30.5%)  p=0.3467  ES=0.46 | -24.4% (-53.8%, 23.5%)  p=0.2609  ES=0.54 | 23.6% (-23.5%, 99.7%)  p=0.3832  ES=0.41 | -5.0% (-41.6%, 54.4%)  p=0.8329  ES=0.10 |
| EEG Gamma Pz-O1 open (uV^2/Hz) | 0.8633 | -12.4% (-45.2%, 39.9%)  p=0.5763  ES=0.28 | -14.5% (-46.5%, 36.6%)  p=0.5097  ES=0.33 | -28.3% (-54.8%, 13.6%)  p=0.1548  ES=0.71 | -12.4% (-44.4%, 38.2%)  p=0.5672  ES=0.28 | 40.5% (-10.9%, 121.5%)  p=0.1416  ES=0.72 | -9.1% (-42.6%, 43.9%)  p=0.6807  ES=0.20 |
| EEG Gamma Pz-O2 closed (uV^2/Hz) | 0.7989 | -0.8% (-38.5%, 59.8%)  p=0.9720  ES=0.02 | -2.8% (-39.6%, 56.4%)  p=0.9054  ES=0.06 | -41.7% (-63.7%, -6.4%)  p=0.0258  ES=1.11 | -26.5% (-53.6%, 16.4%)  p=0.1873  ES=0.63 | 14.3% (-27.2%, 79.3%)  p=0.5579  ES=0.27 | 15.2% (-26.9%, 81.7%)  p=0.5382  ES=0.29 |
| EEG Gamma Pz-O2 open (uV^2/Hz) | 0.3343 | -12.8% (-47.2%, 44.1%)  p=0.5899  ES=0.27 | -27.4% (-56.0%, 19.8%)  p=0.2073  ES=0.63 | -24.6% (-54.1%, 23.9%)  p=0.2609  ES=0.56 | -12.2% (-46.1%, 43.0%)  p=0.5967  ES=0.26 | 22.7% (-24.6%, 99.9%)  p=0.4061  ES=0.41 | 10.5% (-32.4%, 80.6%)  p=0.6867  ES=0.20 |
| EEG Theta Fz-Cz closed (uV^2/Hz) | 0.7182 | 16.2% (-10.2%, 50.4%)  p=0.2503  ES=0.57 | 15.7% (-10.5%, 49.5%)  p=0.2639  ES=0.56 | 16.0% (-10.0%, 49.7%)  p=0.2493  ES=0.57 | -3.7% (-24.9%, 23.3%)  p=0.7623  ES=0.14 | -2.2% (-23.1%, 24.4%)  p=0.8535  ES=0.09 | -21.3% (-38.3%, 0.5%)  p=0.0551  ES=0.91 |
| EEG Theta Fz-Cz open (uV^2/Hz) | 0.1814 | 3.3% (-20.5%, 34.2%)  p=0.8060  ES=0.12 | 16.4% (-10.3%, 51.2%)  p=0.2506  ES=0.58 | 17.9% ( -8.9%, 52.6%)  p=0.2072  ES=0.63 | 2.5% (-20.3%, 31.9%)  p=0.8456  ES=0.09 | 22.6% ( -4.7%, 57.7%)  p=0.1118  ES=0.78 | -0.5% (-22.8%, 28.3%)  p=0.9702  ES=0.02 |
| EEG Theta Pz-O1 closed (uV^2/Hz) | 0.5322 | -0.4% (-39.2%, 63.2%)  p=0.9869  ES=0.01 | 18.5% (-27.6%, 93.9%)  p=0.4962  ES=0.34 | 21.6% (-25.5%, 98.4%)  p=0.4303  ES=0.39 | -20.0% (-50.0%, 28.0%)  p=0.3482  ES=0.45 | -26.8% (-53.8%, 16.0%)  p=0.1813  ES=0.62 | -39.1% (-61.7%, -3.0%)  p=0.0369  ES=0.99 |
| EEG Theta Pz-O1 open (uV^2/Hz) | 0.8667 | 3.0% (-31.1%, 53.9%)  p=0.8850  ES=0.07 | 6.3% (-28.8%, 58.9%)  p=0.7628  ES=0.15 | -4.1% (-35.5%, 42.4%)  p=0.8325  ES=0.10 | -0.3% (-32.4%, 47.1%)  p=0.9882  ES=0.01 | -8.2% (-37.7%, 35.5%)  p=0.6649  ES=0.21 | -15.9% (-43.2%, 24.5%)  p=0.3830  ES=0.43 |
| EEG Theta Pz-O2 closed (uV^2/Hz) | 0.4044 | 13.5% (-28.5%, 80.3%)  p=0.5869  ES=0.27 | 33.8% (-15.6%, 112.2%)  p=0.2121  ES=0.62 | 0.4% (-36.5%, 58.6%)  p=0.9876  ES=0.01 | -22.3% (-50.0%, 20.8%)  p=0.2595  ES=0.54 | -8.0% (-40.2%, 41.5%)  p=0.7014  ES=0.18 | -19.1% (-47.7%, 25.0%)  p=0.3358  ES=0.45 |
| EEG Theta Pz-O2 open (uV^2/Hz) | 0.9005 | 13.1% (-26.1%, 73.3%)  p=0.5668  ES=0.29 | 19.1% (-22.2%, 82.5%)  p=0.4165  ES=0.41 | -4.3% (-37.2%, 45.9%)  p=0.8381  ES=0.10 | 1.1% (-32.8%, 52.2%)  p=0.9567  ES=0.03 | 3.8% (-31.0%, 56.2%)  p=0.8571  ES=0.09 | 4.6% (-30.8%, 57.9%)  p=0.8306  ES=0.10 |

|  | | **Contrasts** | | | | | |
| --- | --- | --- | --- | --- | --- | --- | --- |
|  | | **Adults** | | | **Elderly** | | |
| **Parameter** | **Treatment P-value** | **Day 1 HTL0018318 (25mg) Placebo** | **Day 5 HTL0018318 (25mg) Placebo** | **Day 10 HTL0018318 (25mg) Placebo** | **Day 1 HTL0018318 (25mg) Placebo** | **Day 5 HTL0018318 (25mg) Placebo** | **Day 10 HTL0018318 (25mg) Placebo** |
| Saliva (g) | 0.2181 | -0.456 ( -1.547, 0.635)  p=0.4073  ES=0.40 | 0.095 ( -1.175, 1.366)  p=0.8824  ES=0.08 | 0.875 ( -0.222, 1.973)  p=0.1162  ES=0.76 | -0.145 ( -1.200, 0.909)  p=0.7843  ES=0.13 | 0.087 ( -1.145, 1.319)  p=0.8895  ES=0.08 | 0.217 ( -0.856, 1.289)  p=0.6888  ES=0.19 |
| LSEQ: Getting to sleep (mm) | 0.4138 | -0.98 ( -3.65, 1.70)  p=0.4731  ES=0.49 | -0.38 ( -3.12, 2.36)  p=0.7843  ES=0.19 | -0.76 ( -3.50, 1.98)  p=0.5875  ES=0.38 | 4.56 ( 1.93, 7.19)  p=0.0007  ES=2.30 | 1.16 ( -1.55, 3.87)  p=0.4006  ES=0.58 | -3.21 ( -6.04, -0.37)  p=0.0267  ES=1.62 |
| LSEQ: Quality of sleep (mm) | 0.6614 | -0.82 ( -6.97, 5.33)  p=0.7934  ES=0.17 | 6.29 ( -0.01, 12.58)  p=0.0502  ES=1.31 | 2.50 ( -3.79, 8.80)  p=0.4344  ES=0.52 | -0.74 ( -6.74, 5.25)  p=0.8069  ES=0.16 | 0.81 ( -5.34, 6.97)  p=0.7951  ES=0.17 | -0.54 ( -6.97, 5.90)  p=0.8696  ES=0.11 |
| LSEQ: Awake following sleep (mm) | 0.6424 | -0.89 ( -6.49, 4.71)  p=0.7546  ES=0.18 | -3.28 ( -8.97, 2.42)  p=0.2574  ES=0.67 | -4.92 ( -10.62, 0.77)  p=0.0897  ES=1.00 | -0.27 ( -5.79, 5.25)  p=0.9219  ES=0.06 | 0.67 ( -4.96, 6.30)  p=0.8140  ES=0.14 | -0.33 ( -6.14, 5.47)  p=0.9097  ES=0.07 |
| LSEQ: Behaviour after wake (mm) | 0.2364 | -0.71 ( -6.68, 5.26)  p=0.8149  ES=0.14 | -5.17 ( -11.26, 0.92)  p=0.0956  ES=1.03 | -5.02 ( -11.11, 1.07)  p=0.1057  ES=1.00 | 0.83 ( -5.05, 6.70)  p=0.7816  ES=0.16 | 1.33 ( -4.68, 7.35)  p=0.6627  ES=0.26 | -5.66 ( -11.89, 0.57)  p=0.0750  ES=1.13 |
| Systolic BP supine (mmHg) | 0.3170 | 8.7 ( 1.6, 15.8)  p=0.0166  ES=1.10 | 5.9 ( -1.3, 13.0)  p=0.1061  ES=0.74 | 5.0 ( -2.1, 12.1)  p=0.1676  ES=0.63 | 4.6 ( -2.4, 11.7)  p=0.1936  ES=0.59 | -0.3 ( -7.4, 6.8)  p=0.9426  ES=0.03 | 0.2 ( -7.0, 7.4)  p=0.9554  ES=0.03 |
| Diastolic BP supine (mmHg) | 0.0135 | 4.6 ( -0.3, 9.5)  p=0.0632  ES=0.86 | -4.4 ( -9.3, 0.5)  p=0.0752  ES=0.83 | -5.3 ( -10.2, -0.4)  p=0.0342  ES=0.99 | 0.7 ( -4.0, 5.4)  p=0.7728  ES=0.13 | -1.1 ( -5.8, 3.7)  p=0.6559  ES=0.20 | 0.2 ( -4.6, 5.0)  p=0.9425  ES=0.03 |
| Pulse Rate supine (bpm) | 0.0112 | 3.2 ( -1.5, 8.0)  p=0.1785  ES=0.62 | 7.5 ( 2.7, 12.3)  p=0.0024  ES=1.45 | 3.3 ( -1.5, 8.1)  p=0.1733  ES=0.64 | 6.5 ( 2.0, 11.0)  p=0.0055  ES=1.25 | 7.9 ( 3.3, 12.5)  p=0.0009  ES=1.52 | 5.3 ( 0.7, 10.0)  p=0.0254  ES=1.02 |
| Systolic BP standing (mmHg) | 0.6207 | 6.6 ( -1.1, 14.3)  p=0.0941  ES=0.76 | 0.8 ( -7.0, 8.6)  p=0.8366  ES=0.09 | 3.8 ( -4.0, 11.6)  p=0.3326  ES=0.44 | 0.1 ( -7.8, 7.9)  p=0.9886  ES=0.01 | 1.5 ( -6.4, 9.4)  p=0.7005  ES=0.18 | 0.4 ( -7.6, 8.4)  p=0.9269  ES=0.04 |
| Diastolic BP standing (mmHg) | 0.1009 | 5.0 ( -0.6, 10.6)  p=0.0779  ES=0.81 | -5.8 ( -11.4, -0.2)  p=0.0434  ES=0.94 | -2.5 ( -8.1, 3.2)  p=0.3841  ES=0.40 | 3.7 ( -1.8, 9.2)  p=0.1857  ES=0.60 | -1.0 ( -6.5, 4.6)  p=0.7281  ES=0.16 | -0.8 ( -6.4, 4.8)  p=0.7776  ES=0.13 |
| Pulse Rate standing (bpm) | 0.2866 | 0.3 ( -5.3, 5.9)  p=0.9225  ES=0.04 | 3.7 ( -2.0, 9.3)  p=0.2014  ES=0.59 | -0.1 ( -5.7, 5.6)  p=0.9818  ES=0.01 | 5.1 ( -0.3, 10.5)  p=0.0652  ES=0.82 | 6.4 ( 1.0, 11.9)  p=0.0213  ES=1.03 | 4.5 ( -1.1, 10.1)  p=0.1129  ES=0.72 |
| Systolic BP sup-sta (mmHg) | 0.7415 | 1.7 ( -3.7, 7.1)  p=0.5380  ES=0.27 | 4.6 ( -1.0, 10.1)  p=0.1041  ES=0.72 | 0.7 ( -4.8, 6.2)  p=0.8010  ES=0.11 | 5.5 ( 0.1, 10.9)  p=0.0457  ES=0.87 | -0.7 ( -6.2, 4.8)  p=0.8063  ES=0.11 | 1.0 ( -4.6, 6.7)  p=0.7198  ES=0.16 |
| Diastolic BP sup-sta (mmHg) | 0.9738 | 0.6 ( -2.9, 4.0)  p=0.7441  ES=0.14 | 2.4 ( -1.1, 5.9)  p=0.1850  ES=0.58 | -1.8 ( -5.3, 1.7)  p=0.3042  ES=0.45 | -1.8 ( -5.2, 1.6)  p=0.2883  ES=0.45 | 1.4 ( -2.1, 4.8)  p=0.4337  ES=0.34 | 2.5 ( -1.1, 6.0)  p=0.1716  ES=0.61 |
| Pulse Rate sup-sta (bpm) | 0.5454 | 2.0 ( -1.9, 5.8)  p=0.3160  ES=0.44 | 2.6 ( -1.3, 6.6)  p=0.1877  ES=0.58 | 2.3 ( -1.6, 6.2)  p=0.2529  ES=0.51 | -0.5 ( -4.4, 3.4)  p=0.7969  ES=0.11 | -0.2 ( -4.1, 3.7)  p=0.9095  ES=0.05 | -0.9 ( -4.9, 3.1)  p=0.6670  ES=0.19 |
| Track Performance (%) | 0.3407 | -1.018 ( -3.861, 1.824)  p=0.4779  ES=0.34 | -0.310 ( -3.176, 2.556)  p=0.8300  ES=0.10 | -1.483 ( -4.348, 1.383)  p=0.3064  ES=0.49 | 1.730 ( -1.107, 4.566)  p=0.2284  ES=0.57 | 1.412 ( -1.449, 4.272)  p=0.3290  ES=0.47 | 1.394 ( -1.503, 4.291)  p=0.3414  ES=0.46 |
| N-back corr-incorr/total 0 | 0.1986 | -0.001 ( -0.035, 0.032)  p=0.9476  ES=0.03 | 0.040 ( 0.005, 0.074)  p=0.0245  ES=1.15 | 0.002 ( -0.032, 0.037)  p=0.8942  ES=0.07 | 0.023 ( -0.008, 0.055)  p=0.1485  ES=0.67 | 0.038 ( 0.006, 0.070)  p=0.0212  ES=1.10 | 0.024 ( -0.010, 0.058)  p=0.1667  ES=0.69 |
| N-back corr-incorr/total 1 | 0.0605 | 0.040 ( -0.013, 0.093)  p=0.1386  ES=0.71 | 0.043 ( -0.011, 0.097)  p=0.1172  ES=0.77 | 0.023 ( -0.032, 0.078)  p=0.4174  ES=0.41 | 0.041 ( -0.011, 0.093)  p=0.1223  ES=0.73 | 0.016 ( -0.038, 0.069)  p=0.5614  ES=0.28 | 0.040 ( -0.016, 0.095)  p=0.1609  ES=0.71 |
| N-back corr-incorr/total 2 | 0.0024 | 0.027 ( -0.042, 0.097)  p=0.4337  ES=0.37 | 0.033 ( -0.038, 0.103)  p=0.3640  ES=0.44 | 0.073 ( 0.002, 0.144)  p=0.0436  ES=0.99 | 0.109 ( 0.041, 0.177)  p=0.0020  ES=1.47 | 0.029 ( -0.042, 0.099)  p=0.4243  ES=0.38 | 0.027 ( -0.047, 0.101)  p=0.4690  ES=0.37 |
| N-back mean RT 0 back (msec) | 0.7917 | -13.8 ( -57.5, 29.8)  p=0.5302  ES=0.30 | -18.6 ( -62.8, 25.6)  p=0.4057  ES=0.40 | -40.8 ( -85.2, 3.6)  p=0.0711  ES=0.88 | 1.6 ( -40.9, 44.1)  p=0.9402  ES=0.03 | -1.0 ( -44.1, 42.0)  p=0.9617  ES=0.02 | -0.3 ( -44.3, 43.7)  p=0.9885  ES=0.01 |
| N-back mean RT 1 back (msec) | 0.9521 | 23.5 ( -28.9, 75.9)  p=0.3753  ES=0.42 | 10.6 ( -42.4, 63.5)  p=0.6933  ES=0.19 | -18.8 ( -72.1, 34.5)  p=0.4845  ES=0.34 | 25.8 ( -25.6, 77.3)  p=0.3208  ES=0.46 | 19.2 ( -32.8, 71.2)  p=0.4656  ES=0.34 | 14.0 ( -39.0, 67.0)  p=0.6015  ES=0.25 |
| N-back mean RT 2 back (msec) | 0.3380 | -40.8 ( -114.8, 33.2)  p=0.2763  ES=0.52 | 13.8 ( -61.1, 88.7)  p=0.7158  ES=0.17 | 34.4 ( -40.6, 109.4)  p=0.3646  ES=0.44 | 7.8 ( -64.7, 80.3)  p=0.8314  ES=0.10 | 1.5 ( -72.1, 75.2)  p=0.9669  ES=0.02 | 4.5 ( -70.9, 80.0)  p=0.9051  ES=0.06 |
| MMTImm: Expl Error | 0.0082 | 2.6 ( -2.4, 7.5)  p=0.3047  ES=0.49 | 3.6 ( -1.4, 8.6)  p=0.1565  ES=0.69 | 1.4 ( -3.6, 6.4)  p=0.5720  ES=0.27 | -4.4 ( -9.3, 0.4)  p=0.0718  ES=0.84 | -7.7 ( -12.6, -2.7)  p=0.0025  ES=1.46 | -4.8 ( -9.9, 0.3)  p=0.0632  ES=0.92 |
| MMTImm: Total Moves | 0.0192 | 6.9 ( -3.1, 16.9)  p=0.1743  ES=0.65 | 9.1 ( -1.1, 19.3)  p=0.0799  ES=0.85 | 4.1 ( -6.1, 14.3)  p=0.4261  ES=0.39 | -9.5 ( -19.4, 0.3)  p=0.0584  ES=0.89 | -15.8 ( -25.8, -5.8)  p=0.0023  ES=1.48 | -9.7 ( -20.1, 0.7)  p=0.0662  ES=0.91 |
| MMTImm: Expl Time (msec) | 0.9288 | 4542.0 ( -12226, 21310.3)  p=0.5916  ES=0.25 | 5477.5 ( -11467, 22422.2)  p=0.5223  ES=0.31 | 6090.1 ( -10855, 23034.9)  p=0.4770  ES=0.34 | -6555.0 ( -23252, 10141.6)  p=0.4372  ES=0.37 | -7251.2 ( -24104, 9601.9)  p=0.3948  ES=0.41 | 1252.3 ( -15864, 18368.2)  p=0.8848  ES=0.07 |
| MMTRev: Expl Error | 0.0366 | -2.2 ( -3.9, -0.4)  p=0.0174  ES=1.14 | -0.1 ( -1.9, 1.7)  p=0.9334  ES=0.04 | -1.4 ( -3.2, 0.4)  p=0.1219  ES=0.75 | -2.0 ( -3.8, -0.3)  p=0.0217  ES=1.08 | -1.7 ( -3.5, 0.1)  p=0.0577  ES=0.90 | -1.9 ( -3.7, -0.1)  p=0.0431  ES=0.99 |
| MMTRev: Total Moves | 0.0251 | -4.2 ( -7.8, -0.6)  p=0.0228  ES=1.09 | -0.1 ( -3.8, 3.6)  p=0.9614  ES=0.02 | -2.8 ( -6.4, 0.9)  p=0.1380  ES=0.72 | -4.4 ( -8.0, -0.9)  p=0.0142  ES=1.15 | -4.2 ( -7.8, -0.6)  p=0.0218  ES=1.09 | -4.3 ( -8.0, -0.6)  p=0.0230  ES=1.12 |
| MMTRev: Expl Time (msec) | 0.2267 | -838.2 (-4825.9, 3149.4)  p=0.6781  ES=0.20 | -279.5 (-4346.8, 3787.8)  p=0.8921  ES=0.07 | 64.3 (-4003.0, 4131.5)  p=0.9751  ES=0.02 | -3952.4 (-7911.8, 7.0)  p=0.0504  ES=0.93 | -1405.9 (-5437.7, 2626.0)  p=0.4915  ES=0.33 | 1356.8 (-2807.9, 5521.4)  p=0.5207  ES=0.32 |
| MMTDel: Expl Error | 0.1938 | 0.3 ( -1.1, 1.8)  p=0.6592  ES=0.21 | -0.7 ( -2.1, 0.8)  p=0.3802  ES=0.42 | -0.9 ( -2.4, 0.5)  p=0.2103  ES=0.61 | -1.1 ( -2.5, 0.3)  p=0.1293  ES=0.72 | -0.4 ( -1.9, 1.0)  p=0.5786  ES=0.27 | 0.1 ( -1.4, 1.6)  p=0.9034  ES=0.06 |
| MMTDel: Total Moves | 0.1404 | 0.7 ( -2.2, 3.6)  p=0.6478  ES=0.22 | -1.2 ( -4.2, 1.7)  p=0.4176  ES=0.39 | -1.9 ( -4.9, 1.1)  p=0.2069  ES=0.61 | -2.5 ( -5.4, 0.4)  p=0.0930  ES=0.79 | -0.7 ( -3.7, 2.3)  p=0.6420  ES=0.22 | 0.3 ( -2.7, 3.4)  p=0.8224  ES=0.11 |
| MMTDel: Expl Time (msec) | 0.6223 | 1203.9 (-2991.9, 5399.7)  p=0.5705  ES=0.27 | -2850.2 (-7101.0, 1400.6)  p=0.1866  ES=0.64 | -705.1 (-4955.9, 3545.8)  p=0.7429  ES=0.16 | -1805.4 (-6033.9, 2423.2)  p=0.3989  ES=0.40 | 278.3 (-4007.5, 4564.0)  p=0.8978  ES=0.06 | 1572.8 (-2812.6, 5958.1)  p=0.4788  ES=0.35 |
| Left Pupil/Iris ratio | 0.9442 | 0.01086 (-.02119, 0.04291)  p=0.5024  ES=0.32 | -.01804 (-.05040, 0.01432)  p=0.2709  ES=0.53 | -.01762 (-.04997, 0.01474)  p=0.2822  ES=0.52 | 0.02161 (-.00946, 0.05268)  p=0.1704  ES=0.64 | 0.00636 (-.02501, 0.03773)  p=0.6879  ES=0.19 | 0.00179 (-.03019, 0.03376)  p=0.9118  ES=0.05 |
| Right Pupil/Iris ratio | 0.8405 | 0.01333 (-.02059, 0.04724)  p=0.4366  ES=0.37 | -.01895 (-.05317, 0.01527)  p=0.2739  ES=0.53 | -.01943 (-.05365, 0.01478)  p=0.2620  ES=0.54 | 0.01130 (-.02143, 0.04403)  p=0.4942  ES=0.32 | -.00113 (-.03415, 0.03190)  p=0.9462  ES=0.03 | 0.00168 (-.03194, 0.03530)  p=0.9211  ES=0.05 |
| VAS Alertness (mm) | 0.9337 | -1.75 ( -5.11, 1.60)  p=0.3018  ES=0.49 | -0.41 ( -3.80, 2.98)  p=0.8111  ES=0.11 | -0.69 ( -4.08, 2.70)  p=0.6848  ES=0.19 | 0.57 ( -2.73, 3.86)  p=0.7335  ES=0.16 | 0.81 ( -2.52, 4.13)  p=0.6314  ES=0.23 | -1.41 ( -4.79, 1.97)  p=0.4086  ES=0.40 |
| VAS Calmness (mm) | 0.6199 | -1.80 ( -5.79, 2.19)  p=0.3724  ES=0.42 | 0.83 ( -3.21, 4.87)  p=0.6845  ES=0.19 | -1.77 ( -5.80, 2.27)  p=0.3875  ES=0.42 | 0.10 ( -3.81, 4.01)  p=0.9601  ES=0.02 | -1.20 ( -5.16, 2.77)  p=0.5503  ES=0.28 | -3.01 ( -7.06, 1.04)  p=0.1431  ES=0.71 |
| VAS Mood (mm) | 0.6981 | -2.39 ( -6.08, 1.30)  p=0.2015  ES=0.61 | -0.55 ( -4.27, 3.18)  p=0.7715  ES=0.14 | -2.30 ( -6.03, 1.42)  p=0.2224  ES=0.59 | 1.61 ( -2.00, 5.22)  p=0.3781  ES=0.41 | 1.55 ( -2.10, 5.19)  p=0.4022  ES=0.39 | -0.59 ( -4.29, 3.12)  p=0.7545  ES=0.15 |
| VAS Nausea (log(mm)) | 0.3618 | 0.19 ( 0.06, 0.31)  p=0.0043  ES=1.37 | 0.03 ( -0.10, 0.16)  p=0.6100  ES=0.25 | 0.05 ( -0.08, 0.18)  p=0.4905  ES=0.34 | 0.14 ( 0.01, 0.26)  p=0.0304  ES=1.01 | -0.12 ( -0.25, 0.01)  p=0.0635  ES=0.88 | -0.06 ( -0.20, 0.07)  p=0.3320  ES=0.48 |
| EEG Alpha Fz-Cz closed (uV^2/Hz) | 0.8835 | 5.6% (-28.7%, 56.4%)  p=0.7829  ES=0.14 | 44.2% ( -2.3%, 112.8%)  p=0.0654  ES=0.96 | 63.0% ( 10.1%, 141.4%)  p=0.0153  ES=1.29 | -4.2% (-32.7%, 36.3%)  p=0.8085  ES=0.11 | -12.3% (-38.6%, 25.3%)  p=0.4663  ES=0.35 | -17.9% (-42.7%, 17.7%)  p=0.2804  ES=0.52 |
| EEG Alpha Fz-Cz open (uV^2/Hz) | 0.8005 | -8.4% (-35.9%, 31.0%)  p=0.6291  ES=0.26 | 14.5% (-19.9%, 63.7%)  p=0.4531  ES=0.40 | 47.9% ( 3.3%, 111.7%)  p=0.0327  ES=1.15 | -2.8% (-29.4%, 33.9%)  p=0.8608  ES=0.08 | -2.3% (-29.8%, 35.8%)  p=0.8875  ES=0.07 | -1.6% (-29.3%, 36.9%)  p=0.9222  ES=0.05 |
| EEG Alpha Pz-O1 closed (uV^2/Hz) | 0.6978 | -25.5% (-62.4%, 47.6%)  p=0.3949  ES=0.45 | 1.2% (-48.7%, 99.6%)  p=0.9721  ES=0.02 | 38.9% (-29.9%, 174.9%)  p=0.3418  ES=0.50 | -17.2% (-55.1%, 52.7%)  p=0.5408  ES=0.29 | -47.8% (-71.8%, -3.1%)  p=0.0397  ES=0.99 | -27.7% (-61.2%, 34.8%)  p=0.3037  ES=0.49 |
| EEG Alpha Pz-O1 open (uV^2/Hz) | 0.7428 | -40.7% (-68.9%, 13.4%)  p=0.1130  ES=0.85 | 0.8% (-47.3%, 92.5%)  p=0.9816  ES=0.01 | 27.5% (-33.3%, 143.9%)  p=0.4582  ES=0.40 | -27.9% (-59.4%, 27.9%)  p=0.2595  ES=0.53 | -39.4% (-66.4%, 9.3%)  p=0.0951  ES=0.82 | -30.2% (-61.3%, 25.9%)  p=0.2291  ES=0.59 |
| EEG Alpha Pz-O2 closed (uV^2/Hz) | 0.8168 | -37.7% (-68.0%, 21.4%)  p=0.1621  ES=0.74 | 35.9% (-29.9%, 163.6%)  p=0.3591  ES=0.48 | 52.7% (-21.5%, 197.3%)  p=0.2097  ES=0.67 | -13.8% (-52.3%, 55.9%)  p=0.6205  ES=0.23 | -28.4% (-60.7%, 30.2%)  p=0.2696  ES=0.53 | -0.8% (-45.8%, 81.5%)  p=0.9796  ES=0.01 |
| EEG Alpha Pz-O2 open (uV^2/Hz) | 0.9830 | -39.0% (-68.4%, 17.5%)  p=0.1374  ES=0.81 | 47.1% (-23.7%, 183.4%)  p=0.2456  ES=0.63 | 78.0% ( -7.7%, 243.4%)  p=0.0844  ES=0.94 | -25.4% (-57.8%, 31.8%)  p=0.3089  ES=0.48 | -29.5% (-60.7%, 26.5%)  p=0.2383  ES=0.57 | -16.8% (-53.6%, 49.2%)  p=0.5327  ES=0.30 |
| EEG Beta Fz-Cz closed (uV^2/Hz) | 0.8465 | -4.8% (-27.0%, 24.2%)  p=0.7134  ES=0.19 | 20.9% ( -7.1%, 57.3%)  p=0.1564  ES=0.75 | 8.9% (-16.5%, 42.1%)  p=0.5248  ES=0.34 | 7.1% (-15.4%, 35.5%)  p=0.5667  ES=0.27 | 3.7% (-18.3%, 31.7%)  p=0.7620  ES=0.14 | -1.9% (-22.9%, 24.8%)  p=0.8731  ES=0.08 |
| EEG Beta Fz-Cz open (uV^2/Hz) | 0.7636 | -15.4% (-35.5%, 10.9%)  p=0.2221  ES=0.65 | -1.3% (-24.6%, 29.4%)  p=0.9255  ES=0.05 | -5.2% (-27.7%, 24.4%)  p=0.6991  ES=0.21 | 6.7% (-16.1%, 35.8%)  p=0.5928  ES=0.25 | -1.1% (-22.9%, 26.8%)  p=0.9279  ES=0.04 | 9.3% (-14.8%, 40.3%)  p=0.4792  ES=0.35 |
| EEG Beta Pz-O1 closed (uV^2/Hz) | 0.6816 | -9.1% (-41.0%, 39.9%)  p=0.6608  ES=0.23 | -9.9% (-41.1%, 38.0%)  p=0.6288  ES=0.25 | 3.5% (-32.7%, 59.0%)  p=0.8744  ES=0.08 | -1.1% (-32.5%, 45.0%)  p=0.9549  ES=0.03 | -32.1% (-54.0%, 0.1%)  p=0.0505  ES=0.94 | -17.6% (-44.4%, 22.1%)  p=0.3306  ES=0.47 |
| EEG Beta Pz-O1 open (uV^2/Hz) | 0.5726 | -27.7% (-52.2%, 9.3%)  p=0.1228  ES=0.84 | -22.7% (-48.8%, 16.6%)  p=0.2178  ES=0.66 | -11.7% (-41.5%, 33.3%)  p=0.5511  ES=0.32 | 2.9% (-28.6%, 48.4%)  p=0.8758  ES=0.07 | -24.6% (-48.5%, 10.6%)  p=0.1474  ES=0.73 | -1.1% (-32.6%, 45.0%)  p=0.9535  ES=0.03 |
| EEG Beta Pz-O2 closed (uV^2/Hz) | 0.9537 | -23.4% (-50.2%, 18.0%)  p=0.2240  ES=0.65 | 33.0% (-13.2%, 103.8%)  p=0.1872  ES=0.69 | 25.4% (-18.4%, 92.6%)  p=0.2990  ES=0.55 | -5.8% (-35.8%, 38.3%)  p=0.7593  ES=0.14 | -18.3% (-44.6%, 20.5%)  p=0.3051  ES=0.49 | -1.9% (-33.8%, 45.4%)  p=0.9242  ES=0.05 |
| EEG Beta Pz-O2 open (uV^2/Hz) | 0.9100 | -20.7% (-49.7%, 25.0%)  p=0.3137  ES=0.55 | 21.2% (-23.1%, 90.9%)  p=0.4039  ES=0.45 | 48.0% ( -6.1%, 133.2%)  p=0.0901  ES=0.92 | -3.9% (-35.4%, 43.1%)  p=0.8440  ES=0.09 | -13.9% (-42.9%, 29.8%)  p=0.4720  ES=0.35 | 8.2% (-28.2%, 63.2%)  p=0.7034  ES=0.19 |
| EEG Delta Fz-Cz closed (uV^2/Hz) | 0.9730 | -23.2% (-46.9%, 11.1%)  p=0.1603  ES=0.76 | 16.5% (-18.8%, 67.1%)  p=0.4045  ES=0.44 | -23.0% (-46.6%, 11.1%)  p=0.1608  ES=0.75 | 9.3% (-21.1%, 51.6%)  p=0.5895  ES=0.26 | 32.3% ( -5.3%, 84.9%)  p=0.1003  ES=0.81 | 10.5% (-21.5%, 55.4%)  p=0.5650  ES=0.29 |
| EEG Delta Fz-Cz open (uV^2/Hz) | 0.8000 | 12.8% (-19.3%, 57.5%)  p=0.4778  ES=0.39 | 1.9% (-26.9%, 42.1%)  p=0.9107  ES=0.06 | -14.3% (-38.6%, 19.6%)  p=0.3611  ES=0.49 | -0.7% (-26.1%, 33.6%)  p=0.9639  ES=0.02 | -0.2% (-26.8%, 36.2%)  p=0.9918  ES=0.01 | 30.4% ( -4.4%, 77.9%)  p=0.0932  ES=0.85 |
| EEG Delta Pz-O1 closed (uV^2/Hz) | 0.2120 | -26.2% (-51.7%, 12.8%)  p=0.1595  ES=0.78 | 49.8% ( -1.1%, 126.9%)  p=0.0564  ES=1.03 | 50.5% ( -1.1%, 129.0%)  p=0.0564  ES=1.04 | 18.0% (-18.4%, 70.5%)  p=0.3759  ES=0.42 | -3.5% (-33.8%, 40.6%)  p=0.8499  ES=0.09 | -3.2% (-34.0%, 42.0%)  p=0.8683  ES=0.08 |
| EEG Delta Pz-O1 open (uV^2/Hz) | 0.2881 | -27.0% (-49.8%, 6.1%)  p=0.0987  ES=0.92 | 3.7% (-28.4%, 50.1%)  p=0.8476  ES=0.11 | 21.5% (-16.1%, 76.2%)  p=0.3008  ES=0.57 | 5.2% (-24.2%, 46.2%)  p=0.7594  ES=0.15 | -5.3% (-33.3%, 34.4%)  p=0.7594  ES=0.16 | 12.1% (-21.0%, 59.0%)  p=0.5208  ES=0.33 |
| EEG Delta Pz-O2 closed (uV^2/Hz) | 0.3351 | -36.9% (-58.5%, -4.1%)  p=0.0314  ES=1.19 | 60.0% ( 6.3%, 140.9%)  p=0.0246  ES=1.22 | 47.7% ( -2.3%, 123.3%)  p=0.0640  ES=1.01 | 0.0% (-30.3%, 43.7%)  p=0.9981  ES=0.00 | 19.7% (-17.3%, 73.3%)  p=0.3373  ES=0.47 | 3.3% (-29.1%, 50.5%)  p=0.8657  ES=0.08 |
| EEG Delta Pz-O2 open (uV^2/Hz) | 0.6586 | -31.8% (-53.4%, -0.0%)  p=0.0497  ES=1.11 | 15.2% (-21.0%, 68.1%)  p=0.4600  ES=0.41 | 27.5% (-12.6%, 86.1%)  p=0.2056  ES=0.70 | 2.8% (-26.1%, 43.0%)  p=0.8697  ES=0.08 | 2.4% (-27.8%, 45.1%)  p=0.8945  ES=0.07 | 20.1% (-15.3%, 70.2%)  p=0.3020  ES=0.53 |
| EEG Gamma Fz-Cz closed (uV^2/Hz) | 0.7953 | -4.5% (-23.4%, 19.0%)  p=0.6773  ES=0.22 | -10.8% (-28.2%, 11.0%)  p=0.3023  ES=0.54 | -6.3% (-24.8%, 16.7%)  p=0.5588  ES=0.31 | 3.9% (-14.7%, 26.5%)  p=0.7011  ES=0.18 | -1.0% (-18.9%, 20.9%)  p=0.9211  ES=0.05 | -6.1% (-23.3%, 14.8%)  p=0.5332  ES=0.30 |
| EEG Gamma Fz-Cz open (uV^2/Hz) | 0.2899 | 0.2% (-22.2%, 29.1%)  p=0.9860  ES=0.01 | -12.6% (-32.1%, 12.4%)  p=0.2911  ES=0.56 | -9.1% (-29.4%, 17.1%)  p=0.4567  ES=0.40 | 4.4% (-16.7%, 30.9%)  p=0.7059  ES=0.18 | -11.5% (-30.0%, 12.0%)  p=0.3061  ES=0.51 | 0.9% (-20.2%, 27.7%)  p=0.9397  ES=0.04 |
| EEG Gamma Pz-O1 closed (uV^2/Hz) | 0.9511 | 15.0% (-33.3%, 98.4%)  p=0.6113  ES=0.27 | -6.8% (-45.5%, 59.4%)  p=0.7945  ES=0.14 | -8.5% (-46.8%, 57.4%)  p=0.7459  ES=0.17 | 13.5% (-30.7%, 86.1%)  p=0.6110  ES=0.24 | -11.8% (-46.5%, 45.4%)  p=0.6192  ES=0.24 | -23.3% (-53.8%, 27.3%)  p=0.3019  ES=0.51 |
| EEG Gamma Pz-O1 open (uV^2/Hz) | 0.8633 | -24.5% (-54.2%, 24.6%)  p=0.2693  ES=0.60 | -23.0% (-53.2%, 26.8%)  p=0.3020  ES=0.56 | -22.0% (-52.7%, 28.7%)  p=0.3281  ES=0.53 | 35.3% (-13.5%, 111.6%)  p=0.1832  ES=0.64 | 5.2% (-34.1%, 67.8%)  p=0.8313  ES=0.11 | 12.8% (-29.4%, 80.1%)  p=0.6118  ES=0.26 |
| EEG Gamma Pz-O2 closed (uV^2/Hz) | 0.7989 | 1.8% (-38.6%, 68.9%)  p=0.9444  ES=0.04 | 45.4% (-11.9%, 140.0%)  p=0.1417  ES=0.77 | 4.2% (-37.2%, 72.9%)  p=0.8735  ES=0.08 | 6.8% (-32.5%, 68.9%)  p=0.7777  ES=0.13 | 1.3% (-36.3%, 61.2%)  p=0.9557  ES=0.03 | -3.6% (-39.8%, 54.3%)  p=0.8782  ES=0.07 |
| EEG Gamma Pz-O2 open (uV^2/Hz) | 0.3343 | 1.4% (-40.3%, 72.2%)  p=0.9579  ES=0.03 | 36.7% (-19.4%, 131.8%)  p=0.2428  ES=0.62 | 53.5% ( -9.6%, 160.5%)  p=0.1116  ES=0.85 | 28.4% (-20.2%, 106.7%)  p=0.2993  ES=0.49 | 19.2% (-27.0%, 94.6%)  p=0.4787  ES=0.35 | 27.7% (-21.8%, 108.7%)  p=0.3251  ES=0.48 |
| EEG Theta Fz-Cz closed (uV^2/Hz) | 0.7182 | -1.2% (-25.3%, 30.6%)  p=0.9294  ES=0.05 | 9.6% (-16.7%, 44.2%)  p=0.5087  ES=0.35 | -0.6% (-24.8%, 31.2%)  p=0.9635  ES=0.02 | 3.2% (-19.3%, 31.8%)  p=0.8019  ES=0.12 | -1.5% (-23.3%, 26.5%)  p=0.9055  ES=0.06 | -19.0% (-37.2%, 4.4%)  p=0.1024  ES=0.81 |
| EEG Theta Fz-Cz open (uV^2/Hz) | 0.1814 | -0.7% (-25.2%, 31.7%)  p=0.9589  ES=0.03 | 8.5% (-18.2%, 44.0%)  p=0.5663  ES=0.31 | 7.8% (-18.8%, 43.3%)  p=0.5997  ES=0.29 | 14.0% (-11.1%, 46.1%)  p=0.2989  ES=0.50 | 7.2% (-17.3%, 38.8%)  p=0.5970  ES=0.26 | 19.0% ( -8.2%, 54.1%)  p=0.1867  ES=0.66 |
| EEG Theta Pz-O1 closed (uV^2/Hz) | 0.5322 | -9.8% (-46.8%, 52.9%)  p=0.6992  ES=0.21 | 47.2% (-12.7%, 148.0%)  p=0.1447  ES=0.77 | 75.3% ( 3.6%, 196.6%)  p=0.0368  ES=1.12 | -9.3% (-43.2%, 44.8%)  p=0.6806  ES=0.19 | -34.0% (-58.9%, 6.1%)  p=0.0854  ES=0.83 | -16.6% (-48.4%, 34.7%)  p=0.4538  ES=0.36 |
| EEG Theta Pz-O1 open (uV^2/Hz) | 0.8667 | -26.6% (-52.4%, 13.1%)  p=0.1594  ES=0.77 | 10.2% (-28.3%, 69.5%)  p=0.6547  ES=0.24 | 38.3% (-10.2%, 113.0%)  p=0.1392  ES=0.80 | -9.4% (-38.2%, 32.7%)  p=0.6086  ES=0.25 | -23.2% (-48.5%, 14.5%)  p=0.1933  ES=0.66 | -0.3% (-33.2%, 48.6%)  p=0.9867  ES=0.01 |
| EEG Theta Pz-O2 closed (uV^2/Hz) | 0.4044 | -24.4% (-54.0%, 24.3%)  p=0.2673  ES=0.60 | 99.0% ( 21.9%, 225.0%)  p=0.0064  ES=1.47 | 75.0% ( 6.7%, 187.0%)  p=0.0270  ES=1.19 | -3.9% (-37.9%, 48.7%)  p=0.8573  ES=0.08 | -9.4% (-41.8%, 41.2%)  p=0.6612  ES=0.21 | 0.3% (-36.0%, 57.3%)  p=0.9878  ES=0.01 |
| EEG Theta Pz-O2 open (uV^2/Hz) | 0.9005 | -28.7% (-55.1%, 13.2%)  p=0.1501  ES=0.79 | 40.6% (-11.3%, 122.9%)  p=0.1459  ES=0.80 | 61.3% ( 1.6%, 155.8%)  p=0.0426  ES=1.12 | -5.3% (-36.7%, 41.8%)  p=0.7897  ES=0.13 | -13.7% (-43.2%, 31.3%)  p=0.4882  ES=0.34 | 13.4% (-25.4%, 72.5%)  p=0.5526  ES=0.30 |

## Summary PD table elderly 35 mg HTL0018318

|  | | **Contrasts** | | |
| --- | --- | --- | --- | --- |
| **Parameter** | **Treatment P-value** | **Day 1 HTL0018318 (20+35mg) Placebo** | **Day 5 HTL0018318 (20+35mg) Placebo** | **Day 10 HTL0018318 (20+35mg) Placebo** |
| Saliva (g) | 0.3152 | 0.251 ( -1.002, 1.503)  p=0.6797  ES=0.23 | 1.187 ( -0.217, 2.592)  p=0.0949  ES=0.90 | 0.710 ( -0.544, 1.964)  p=0.2506  ES=0.64 |
| LSEQ: Getting to sleep (mm) | 0.1291 | 3.30 ( -0.14, 6.74)  p=0.0599  ES=0.96 | 2.57 ( -0.87, 6.01)  p=0.1412  ES=0.75 | 1.60 ( -1.87, 5.08)  p=0.3612  ES=0.47 |
| LSEQ: Quality of sleep (mm) | 0.4935 | -2.84 ( -9.32, 3.65)  p=0.3833  ES=0.44 | -0.17 ( -6.65, 6.31)  p=0.9583  ES=0.03 | 1.58 ( -4.95, 8.10)  p=0.6290  ES=0.25 |
| LSEQ: Awake following sleep (mm) | 0.6412 | -3.58 ( -8.54, 1.38)  p=0.1542  ES=0.72 | 0.98 ( -3.98, 5.94)  p=0.6955  ES=0.20 | -0.34 ( -5.33, 4.66)  p=0.8940  ES=0.07 |
| LSEQ: Behaviour after wake (mm) | 0.9039 | -5.30 ( -11.67, 1.08)  p=0.1007  ES=0.83 | 0.83 ( -5.54, 7.21)  p=0.7935  ES=0.13 | -3.00 ( -9.41, 3.40)  p=0.3489  ES=0.47 |
| Systolic BP supine (mmHg) | 0.5316 | 7.6 ( -0.2, 15.4)  p=0.0572  ES=1.02 | -0.8 ( -8.6, 7.0)  p=0.8313  ES=0.11 | 3.5 ( -4.3, 11.3)  p=0.3691  ES=0.47 |
| Diastolic BP supine (mmHg) | 0.8764 | -0.0 ( -5.8, 5.8)  p=0.9929  ES=0.01 | -4.4 ( -10.2, 1.4)  p=0.1299  ES=0.91 | -2.7 ( -8.6, 3.1)  p=0.3385  ES=0.57 |
| Pulse Rate supine (bpm) | 0.0072 | 9.5 ( 4.2, 14.7)  p=0.0012  ES=2.01 | 10.0 ( 4.7, 15.3)  p=0.0007  ES=2.12 | 6.4 ( 1.1, 11.7)  p=0.0193  ES=1.36 |
| Systolic BP standing (mmHg) | 0.1835 | 4.9 ( -4.6, 14.3)  p=0.2982  ES=0.53 | 2.6 ( -6.8, 12.0)  p=0.5751  ES=0.28 | 7.8 ( -1.7, 17.3)  p=0.1020  ES=0.84 |
| Diastolic BP standing (mmHg) | 0.2787 | -1.7 ( -5.8, 2.3)  p=0.3889  ES=0.47 | -6.4 ( -10.5, -2.4)  p=0.0027  ES=1.73 | -6.1 ( -10.2, -2.0)  p=0.0043  ES=1.65 |
| Pulse Rate standing (bpm) | 0.0664 | 8.1 ( 1.4, 14.8)  p=0.0205  ES=1.25 | 8.0 ( 1.3, 14.7)  p=0.0221  ES=1.23 | 4.2 ( -2.5, 11.0)  p=0.2033  ES=0.66 |
| Systolic BP sup-sta (mmHg) | 0.3699 | 3.7 ( -2.8, 10.1)  p=0.2576  ES=0.58 | -2.3 ( -8.7, 4.2)  p=0.4758  ES=0.37 | -3.2 ( -9.7, 3.2)  p=0.3179  ES=0.52 |
| Diastolic BP sup-sta (mmHg) | 0.9895 | 0.3 ( -3.0, 3.6)  p=0.8712  ES=0.08 | 0.7 ( -2.6, 3.9)  p=0.6865  ES=0.21 | 2.1 ( -1.2, 5.4)  p=0.2124  ES=0.64 |
| Pulse Rate sup-sta (bpm) | 0.4673 | 1.1 ( -2.4, 4.6)  p=0.5224  ES=0.33 | 1.9 ( -1.6, 5.3)  p=0.2824  ES=0.56 | 2.0 ( -1.5, 5.4)  p=0.2588  ES=0.59 |
| Track Performance (%) | 0.8745 | 0.689 ( -2.018, 3.396)  p=0.6002  ES=0.30 | -0.824 ( -3.530, 1.883)  p=0.5316  ES=0.36 | -1.012 ( -3.722, 1.697)  p=0.4438  ES=0.44 |
| N-back corr-incorr/total 0 | 0.9894 | 0.001 ( -0.036, 0.037)  p=0.9751  ES=0.02 | 0.010 ( -0.027, 0.046)  p=0.5958  ES=0.27 | -0.007 ( -0.044, 0.029)  p=0.6853  ES=0.20 |
| N-back corr-incorr/total 1 | 0.4337 | 0.039 ( -0.048, 0.126)  p=0.3681  ES=0.45 | 0.019 ( -0.069, 0.107)  p=0.6618  ES=0.22 | 0.027 ( -0.060, 0.115)  p=0.5292  ES=0.32 |
| N-back corr-incorr/total 2 | 0.4347 | 0.061 ( -0.030, 0.152)  p=0.1846  ES=0.67 | 0.030 ( -0.062, 0.122)  p=0.5144  ES=0.33 | 0.009 ( -0.083, 0.101)  p=0.8429  ES=0.10 |
| N-back mean RT 0 back (msec) | 0.9029 | 11.7 ( -31.3, 54.7)  p=0.5790  ES=0.28 | -7.4 ( -50.7, 35.8)  p=0.7258  ES=0.18 | -0.2 ( -43.4, 42.9)  p=0.9917  ES=0.01 |
| N-back mean RT 1 back (msec) | 0.8019 | 3.6 ( -54.2, 61.3)  p=0.8984  ES=0.07 | 22.2 ( -35.7, 80.1)  p=0.4345  ES=0.41 | 4.7 ( -53.1, 62.5)  p=0.8673  ES=0.09 |
| N-back mean RT 2 back (msec) | 0.5763 | -11.0 ( -115.7, 93.7)  p=0.8289  ES=0.11 | -23.5 ( -128.5, 81.4)  p=0.6455  ES=0.23 | -45.4 ( -150.3, 59.4)  p=0.3770  ES=0.45 |
| MMTImm: Expl Error | 0.0201 | -8.9 ( -15.7, -2.1)  p=0.0118  ES=1.32 | -8.2 ( -15.0, -1.4)  p=0.0196  ES=1.22 | -1.6 ( -8.4, 5.2)  p=0.6317  ES=0.24 |
| MMTImm: Total Moves | 0.0319 | -18.4 ( -33.1, -3.6)  p=0.0167  ES=1.26 | -16.8 ( -31.6, -2.1)  p=0.0271  ES=1.15 | -2.9 ( -17.8, 11.9)  p=0.6903  ES=0.20 |
| MMTImm: Expl Time (msec) | 0.3440 | -22756 ( -48876, 3363.7)  p=0.0841  ES=1.05 | -11539 ( -37659, 14581.5)  p=0.3673  ES=0.53 | 1069.6 ( -25065, 27204.0)  p=0.9327  ES=0.05 |
| MMTRev: Expl Error | 0.0232 | -2.9 ( -5.7, -0.1)  p=0.0457  ES=1.05 | -3.3 ( -6.1, -0.5)  p=0.0230  ES=1.20 | -2.6 ( -5.4, 0.2)  p=0.0718  ES=0.93 |
| MMTRev: Total Moves | 0.0184 | -6.1 ( -11.9, -0.3)  p=0.0392  ES=1.08 | -7.3 ( -13.1, -1.5)  p=0.0156  ES=1.29 | -5.6 ( -11.4, 0.2)  p=0.0578  ES=0.99 |
| MMTRev: Expl Time (msec) | 0.0843 | -6962.2 ( -13044, -880.2)  p=0.0265  ES=1.29 | -5217.8 ( -11279, 843.0)  p=0.0883  ES=0.97 | -1334.5 (-7406.1, 4737.1)  p=0.6545  ES=0.25 |
| MMTDel: Expl Error | 0.5314 | -0.4 ( -2.4, 1.5)  p=0.6400  ES=0.24 | -0.5 ( -2.4, 1.5)  p=0.6234  ES=0.25 | 0.3 ( -1.7, 2.2)  p=0.7817  ES=0.14 |
| MMTDel: Total Moves | 0.5242 | -1.2 ( -5.2, 2.7)  p=0.5333  ES=0.32 | -0.9 ( -4.8, 3.0)  p=0.6460  ES=0.23 | 0.6 ( -3.4, 4.5)  p=0.7701  ES=0.15 |
| MMTDel: Expl Time (msec) | 0.2883 | -6004.7 ( -11942, -67.8)  p=0.0476  ES=1.13 | -2884.0 (-8820.9, 3053.0)  p=0.3257  ES=0.54 | 716.1 (-5234.9, 6667.1)  p=0.8058  ES=0.14 |
| Left Pupil/Iris ratio | 0.4717 | 0.00848 (-.02333, 0.04029)  p=0.5855  ES=0.28 | 0.00748 (-.02423, 0.03918)  p=0.6290  ES=0.25 | 0.01584 (-.01590, 0.04757)  p=0.3115  ES=0.53 |
| Right Pupil/Iris ratio | 0.8443 | -.00533 (-.03199, 0.02133)  p=0.6835  ES=0.21 | -.00921 (-.03576, 0.01735)  p=0.4807  ES=0.37 | 0.00895 (-.01764, 0.03554)  p=0.4935  ES=0.36 |
| VAS Alertness (mm) | 0.9256 | 0.20 ( -4.12, 4.52)  p=0.9240  ES=0.05 | -0.21 ( -4.53, 4.11)  p=0.9203  ES=0.05 | -1.02 ( -5.35, 3.31)  p=0.6287  ES=0.24 |
| VAS Calmness (mm) | 0.3528 | -2.12 ( -5.77, 1.52)  p=0.2423  ES=0.63 | -0.97 ( -4.62, 2.67)  p=0.5881  ES=0.29 | -1.62 ( -5.28, 2.05)  p=0.3742  ES=0.48 |
| VAS Mood (mm) | 0.8361 | -0.24 ( -3.44, 2.95)  p=0.8755  ES=0.08 | -0.37 ( -3.56, 2.83)  p=0.8137  ES=0.12 | -0.92 ( -4.13, 2.28)  p=0.5564  ES=0.30 |
| VAS Nausea (log(mm)) | 0.2920 | -0.03 ( -0.17, 0.12)  p=0.6872  ES=0.21 | -0.11 ( -0.25, 0.04)  p=0.1368  ES=0.80 | -0.09 ( -0.23, 0.06)  p=0.2386  ES=0.63 |
| EEG Alpha Fz-Cz closed (uV^2/Hz) | 0.1310 | 33.7% (-29.1%, 152.2%)  p=0.3487  ES=0.58 | 75.4% ( -7.3%, 232.0%)  p=0.0807  ES=1.12 | 63.7% (-13.3%, 208.9%)  p=0.1204  ES=0.98 |
| EEG Alpha Fz-Cz open (uV^2/Hz) | 0.3614 | 28.2% (-32.3%, 142.9%)  p=0.4226  ES=0.49 | 33.1% (-30.0%, 152.9%)  p=0.3605  ES=0.56 | 28.6% (-32.2%, 143.9%)  p=0.4179  ES=0.49 |
| EEG Alpha Pz-O1 closed (uV^2/Hz) | 0.0344 | -41.1% (-71.7%, 22.7%)  p=0.1477  ES=0.75 | -60.8% (-81.3%, -18.1%)  p=0.0154  ES=1.33 | -55.4% (-78.6%, -7.1%)  p=0.0329  ES=1.15 |
| EEG Alpha Pz-O1 open (uV^2/Hz) | 0.1400 | -24.1% (-61.6%, 50.2%)  p=0.4080  ES=0.46 | -51.3% (-75.5%, -2.9%)  p=0.0419  ES=1.20 | -43.3% (-71.4%, 12.4%)  p=0.0990  ES=0.95 |
| EEG Alpha Pz-O2 closed (uV^2/Hz) | 0.1033 | -21.3% (-49.4%, 22.6%)  p=0.2749  ES=0.57 | -33.0% (-57.2%, 4.8%)  p=0.0773  ES=0.95 | -31.1% (-55.8%, 7.4%)  p=0.0959  ES=0.88 |
| EEG Alpha Pz-O2 open (uV^2/Hz) | 0.1803 | -16.7% (-51.2%, 42.3%)  p=0.4853  ES=0.39 | -45.1% (-68.1%, -5.6%)  p=0.0317  ES=1.27 | -28.1% (-58.0%, 23.2%)  p=0.2166  ES=0.70 |
| EEG Beta Fz-Cz closed (uV^2/Hz) | 0.4197 | -2.7% (-31.5%, 38.3%)  p=0.8735  ES=0.10 | 25.7% (-11.9%, 79.3%)  p=0.1942  ES=0.81 | 15.8% (-18.6%, 64.6%)  p=0.3948  ES=0.52 |
| EEG Beta Fz-Cz open (uV^2/Hz) | 0.3626 | 10.8% (-20.7%, 55.0%)  p=0.5269  ES=0.38 | 16.3% (-17.0%, 63.0%)  p=0.3611  ES=0.56 | 10.6% (-21.0%, 54.7%)  p=0.5385  ES=0.37 |
| EEG Beta Pz-O1 closed (uV^2/Hz) | 0.0071 | -30.3% (-54.4%, 6.6%)  p=0.0918  ES=0.88 | -53.2% (-69.6%, -28.2%)  p=0.0013  ES=1.86 | -40.6% (-61.2%, -9.0%)  p=0.0188  ES=1.27 |
| EEG Beta Pz-O1 open (uV^2/Hz) | 0.0379 | -22.4% (-49.1%, 18.3%)  p=0.2261  ES=0.67 | -46.1% (-65.0%, -17.1%)  p=0.0065  ES=1.62 | -35.5% (-57.9%, -1.4%)  p=0.0435  ES=1.15 |
| EEG Beta Pz-O2 closed (uV^2/Hz) | 0.5559 | -1.8% (-28.9%, 35.6%)  p=0.9072  ES=0.06 | -11.4% (-36.2%, 23.1%)  p=0.4580  ES=0.41 | -7.7% (-33.3%, 27.7%)  p=0.6162  ES=0.27 |
| EEG Beta Pz-O2 open (uV^2/Hz) | 0.9052 | 10.4% (-23.5%, 59.3%)  p=0.5855  ES=0.30 | -23.9% (-47.7%, 10.7%)  p=0.1473  ES=0.84 | 3.4% (-28.6%, 49.8%)  p=0.8532  ES=0.10 |
| EEG Delta Fz-Cz closed (uV^2/Hz) | 0.2383 | -11.9% (-39.8%, 28.9%)  p=0.5004  ES=0.35 | -10.3% (-39.1%, 32.2%)  p=0.5722  ES=0.30 | -25.0% (-48.8%, 10.0%)  p=0.1350  ES=0.79 |
| EEG Delta Fz-Cz open (uV^2/Hz) | 0.6840 | 3.7% (-26.5%, 46.3%)  p=0.8300  ES=0.12 | -10.3% (-36.9%, 27.6%)  p=0.5333  ES=0.36 | -4.8% (-32.7%, 34.6%)  p=0.7720  ES=0.16 |
| EEG Delta Pz-O1 closed (uV^2/Hz) | 0.0324 | -31.0% (-59.4%, 17.3%)  p=0.1616  ES=0.74 | -40.9% (-65.4%, 1.0%)  p=0.0540  ES=1.05 | -47.3% (-69.1%, -10.1%)  p=0.0207  ES=1.28 |
| EEG Delta Pz-O1 open (uV^2/Hz) | 0.0527 | -15.3% (-41.8%, 23.2%)  p=0.3736  ES=0.49 | -41.6% (-60.4%, -13.9%)  p=0.0079  ES=1.58 | -27.5% (-50.4%, 6.1%)  p=0.0952  ES=0.94 |
| EEG Delta Pz-O2 closed (uV^2/Hz) | 0.0176 | -36.4% (-56.5%, -7.1%)  p=0.0208  ES=1.24 | -35.8% (-56.4%, -5.6%)  p=0.0255  ES=1.22 | -25.2% (-48.9%, 9.4%)  p=0.1293  ES=0.80 |
| EEG Delta Pz-O2 open (uV^2/Hz) | 0.0351 | -13.2% (-37.6%, 20.7%)  p=0.3907  ES=0.47 | -44.3% (-60.4%, -21.5%)  p=0.0013  ES=1.95 | -15.3% (-39.4%, 18.5%)  p=0.3239  ES=0.55 |
| EEG Gamma Fz-Cz closed (uV^2/Hz) | 0.9178 | -6.8% (-23.7%, 13.9%)  p=0.4752  ES=0.38 | 2.3% (-16.5%, 25.3%)  p=0.8164  ES=0.12 | -1.1% (-19.1%, 20.9%)  p=0.9135  ES=0.06 |
| EEG Gamma Fz-Cz open (uV^2/Hz) | 0.2762 | 17.1% ( -6.6%, 46.8%)  p=0.1624  ES=0.79 | 11.3% (-11.6%, 40.1%)  p=0.3461  ES=0.54 | 4.2% (-17.0%, 30.9%)  p=0.7080  ES=0.21 |
| EEG Gamma Pz-O1 closed (uV^2/Hz) | 0.0463 | -18.5% (-51.3%, 36.4%)  p=0.4206  ES=0.42 | -44.1% (-66.8%, -5.8%)  p=0.0304  ES=1.18 | -40.8% (-64.7%, -0.8%)  p=0.0466  ES=1.06 |
| EEG Gamma Pz-O1 open (uV^2/Hz) | 0.1004 | -21.4% (-53.2%, 32.1%)  p=0.3486  ES=0.52 | -37.5% (-63.2%, 6.3%)  p=0.0809  ES=1.00 | -40.5% (-64.8%, 0.6%)  p=0.0524  ES=1.11 |
| EEG Gamma Pz-O2 closed (uV^2/Hz) | 0.7379 | 0.1% (-36.6%, 58.1%)  p=0.9954  ES=0.00 | 2.1% (-35.7%, 62.0%)  p=0.9286  ES=0.05 | -16.5% (-47.2%, 32.0%)  p=0.4256  ES=0.41 |
| EEG Gamma Pz-O2 open (uV^2/Hz) | 0.5985 | -3.6% (-44.8%, 68.5%)  p=0.8943  ES=0.07 | -25.9% (-58.0%, 30.8%)  p=0.2877  ES=0.59 | -13.1% (-50.5%, 52.5%)  p=0.6114  ES=0.28 |
| EEG Theta Fz-Cz closed (uV^2/Hz) | 0.9164 | -7.3% (-37.4%, 37.3%)  p=0.6911  ES=0.23 | 9.8% (-26.1%, 63.1%)  p=0.6285  ES=0.28 | 4.5% (-29.5%, 54.9%)  p=0.8163  ES=0.13 |
| EEG Theta Fz-Cz open (uV^2/Hz) | 0.2192 | 27.4% ( -8.6%, 77.6%)  p=0.1436  ES=0.93 | 23.2% (-12.0%, 72.3%)  p=0.2103  ES=0.80 | 12.0% (-19.7%, 56.3%)  p=0.4850  ES=0.43 |
| EEG Theta Pz-O1 closed (uV^2/Hz) | 0.0586 | -41.1% (-70.0%, 15.8%)  p=0.1177  ES=0.82 | -53.1% (-76.2%, -7.5%)  p=0.0308  ES=1.17 | -40.9% (-69.9%, 16.3%)  p=0.1205  ES=0.81 |
| EEG Theta Pz-O1 open (uV^2/Hz) | 0.1426 | -17.1% (-51.4%, 41.4%)  p=0.4730  ES=0.39 | -46.2% (-68.7%, -7.6%)  p=0.0265  ES=1.29 | -33.8% (-61.3%, 13.3%)  p=0.1255  ES=0.86 |
| EEG Theta Pz-O2 closed (uV^2/Hz) | 0.3455 | -21.0% (-44.3%, 12.1%)  p=0.1787  ES=0.72 | -15.1% (-40.6%, 21.3%)  p=0.3565  ES=0.50 | -3.3% (-31.9%, 37.4%)  p=0.8477  ES=0.10 |
| EEG Theta Pz-O2 open (uV^2/Hz) | 0.5837 | 5.5% (-25.2%, 48.8%)  p=0.7517  ES=0.18 | -28.4% (-49.7%, 1.9%)  p=0.0631  ES=1.10 | -3.9% (-32.1%, 36.0%)  p=0.8169  ES=0.13 |

1. References

1. Borland RG, Nicholson AN. Visual motor co-ordination and dynamic visual acuity. Br J Clin Pharmacol. 1984;18 Suppl 1:69S-72S.

2. Hart EP, Alvarez-Jimenez R, Davidse E, Doll RJ, Cohen AF, Van Gerven JMA, et al. A Computerized Test Battery to Study Pharmacodynamic Effects on the Central Nervous System of Cholinergic Drugs in Early Phase Drug Development. JoVE. 2019(144):e56569.

3. Baakman AC, t Hart E, Kay DG, Stevens J, Klaassen ES, Maelicke A, et al. First in human study with a prodrug of galantamine: Improved benefit-risk ratio? Alzheimer's & Dementia: Translational Research & Clinical Interventions. 2016;2(1):13-22.

4. Milner B. Visually-guided maze learning in man: Effects of bilateral hippocampal, bilateral frontal, and unilateral cerebral lesions. Neuropsychologia. 1965;3(4):317-38.

5. Alvarez-Jimenez R, Hart EP, Prins S, de Kam M, van Gerven JMA, Cohen AF, et al. Reversal of mecamylamine-induced effects in healthy subjects by nicotine receptor agonists: Cognitive and (electro) physiological responses. Br J Clin Pharmacol. 2018;84(5):888-99.

6. Snyder PJ, Bednar MM, Cromer JR, Maruff P. Reversal of scopolamine-induced deficits with a single dose of donepezil, an acetylcholinesterase inhibitor. Alzheimers Dement. 2005;1(2):126-35.

7. Baakman AC, Alvarez-Jimenez R, Rissmann R, Klaassen ES, Stevens J, Goulooze SC, et al. An anti-nicotinic cognitive challenge model using mecamylamine in comparison with the anti-muscarinic cognitive challenge using scopolamine. Br J Clin Pharmacol. 2017;83(8):1676-87.

8. Rombouts SA, Barkhof F, Van Meel CS, Scheltens P. Alterations in brain activation during cholinergic enhancement with rivastigmine in Alzheimer's disease. Journal of neurology, neurosurgery, and psychiatry. 2002;73(6):665-71.

9. Lim HK, Juh R, Pae CU, Lee BT, Yoo SS, Ryu SH, et al. Altered verbal working memory process in patients with Alzheimer's disease: an fMRI investigation. Neuropsychobiology. 2008;57(4):181-7.

10. Twa MD, Bailey MD, Hayes J, Bullimore M. Estimation of pupil size by digital photography. Journal of cataract and refractive surgery. 2004;30(2):381-9.

11. Borghans L, Sambeth A, Prickaerts J, Ramaekers JG, Blokland A. The effects of the soluble guanylate cyclase stimulator riociguat on memory performance in healthy volunteers with a biperiden-induced memory impairment. Psychopharmacology (Berl). 2018;235(8):2407-16.

12. Babiloni C, Del Percio C, Bordet R, Bourriez JL, Bentivoglio M, Payoux P, et al. Effects of acetylcholinesterase inhibitors and memantine on resting-state electroencephalographic rhythms in Alzheimer's disease patients. Clin Neurophysiol. 2013;124(5):837-50.

13. Hedges D, Janis R, Mickelson S, Keith C, Bennett D, Brown BL. P300 Amplitude in Alzheimer's Disease: A Meta-Analysis and Meta-Regression. Clinical EEG and neuroscience. 2016;47(1):48-55.

14. Riekkinen P, Jr., Paakkonen A, Karhu J, Partanen J, Soininen H, Laakso M, et al. THA disrupts mismatch negativity in Alzheimer disease. Psychopharmacology (Berl). 1997;133(2):203-6.

15. Papadaniil CD, Kosmidou VE, Tsolaki A, Tsolaki M, Kompatsiaris IY, Hadjileontiadis LJ. Cognitive MMN and P300 in mild cognitive impairment and Alzheimer's disease: A high density EEG-3D vector field tomography approach. Brain research. 2016;1648(Pt A):425-33.

16. Bond A, Lader M. The use of analogue scales in rating subjective feelings. British Journal of Medical Psychology. 1974;47(3):211-8.

17. Parrott AC, Hindmarch I. The Leeds Sleep Evaluation Questionnaire in psychopharmacological investigations - a review. Psychopharmacology (Berl). 1980;71(2):173-9.
